# Selective methylation of kaempferol via benzylation and deacetylation of kaempferol acetates

**DOI:** 10.3762/bjoc.11.33

**Published:** 2015-02-25

**Authors:** Qinggang Mei, Chun Wang, Weicheng Yuan, Guolin Zhang

**Affiliations:** 1 Chengdu Institute of Organic Chemistry, Chinese Academy of Sciences, Chengdu 610041, China; 2 Chengdu Institute of Biology, Chinese Academy of Sciences, Chengdu 610041, China; 3 University of Chinese Academy of Sciences, Beijing 100049, China

**Keywords:** benzylation, deacetylation, kaempferol, methylation, regioselectivity

## Abstract

A strategy for selective mono-, di- and tri-*O*-methylation of kaempferol, predominantly on the basis of selective benzylation and controllable deacetylation of kaempferol acetates, was developed. From the selective deacetylation and benzylation of kaempferol tetraacetate (**1**), 3,4′,5,-tri-*O*-acetylkaempferol (**2**) and 7-*O*-benzyl-3,4′5,-tri-*O*-acetylkaempferol (**8**) were obtained, respectively. By controllable deacetylation and followed selective or direct methylation of these two intermediates, eight *O*-methylated kaempferols were prepared with 51–77% total yields from kaempferol.

## Introduction

**Figure 1:**
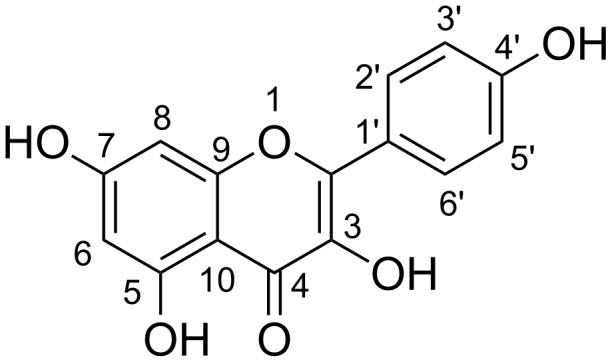
Kaempferol.

Kaempferol [2-(4-hydroxyphenyl)-3,5,7-trihydroxychromen-4-one] (Figure 1) and its derivatives are widely distributed in plants such as beans, broccoli, strawberries, teas, and propolis [1–2]. They are well known to have a wide range of pharmacological activities [3]. Recent studies have indicated that diets abundant in kaempferol-rich fruits and vegetables may have prophylactic effects against the development of coronary heart disease and certain cancers [4–5]. Among them, methylated derivatives of kaempferol have attracted attention for a long time due to their broad spectrum of biological activities, including induction of apoptosis in adipocytes, anti-inflammation and anti-atherogenic properties [6–7]. 3-*O*-Methylkaempferol evidently possesses antiviral [8], antimicrobial [9] and cytotoxic [10] activities. 7-*O*-Methylkaempferol (rhamnocitrin) shows a very strong inhibition against platelet aggregation induced by sodium arachidonate [11], and antibacterial [12], anti-atherogenic [13], anti-inflammatory and antispasmodic activities [14]. 4′-*O*-Methylkaempferol (kaempferide) is reported to reduce hypoxia-inducible factor (HIF)-1-dependent luciferase expression [15], and possess potent antiproliferative effects on the human colon cancer cell line Caco-2 [16]. 3,7-Di-*O*-methylkaempferol (kumatakenin) demonstrates high inhibitory activity against porcine pancreas α-amylase [7]. 4′,7-Di-*O*-methylkaempferol exhibits modest cytotoxicity [17]. These methylated derivatives of flavonols are found as natural products in relative low quantity, whereas kaempferol is relatively rich in plants. Therefore, it is quite worthwhile to develop procedures for the preparation of methylated derivatives of kaempferol.

On the other hand, *O*-methylation is an effective approach to protect the reactive hydroxy groups of flavonoids and a strategy to improve the physicochemical properties such as solubility and intracellular compartmentation [18]. Some of the *O*-alkylated flavonoids possess biological activities, and some have been used for the preparation of natural and therapeutic products of biological significance. To date, however, the regioselective direct *O*-alkylation of polyhydroxy favonoids is still of challenge. Until recently, selective *O*-methylation of quercetin was carried out by the protection of phenolic functions with benzyl or diphenylmethylene ketal [19]. Quercetin was converted to pentabenzylquercetin and thereby four mono-methylated isomers were synthesized through selective deprotection [20]. The regioselective *O*-methylation of kaempferol, to the best of our knowledge, has not been reported up to now.

We tried to directly methylate kaempferol using dimethyl sulfate as methylating reagent, but the methylation was uncontrollable and resulted in a mixture of multiple methylated products. The direct methylation of the hydroxy groups in kaempferol is non-selective. We turned to the selective or controllable hydrolysis of kaempferol tetraacetate (**1**) intending to get the specific hydroxy group free for methylation. The solubility of acetylated kaempferols is improved, and the conditions for the methylation possess more possibilities. As a part of our ongoing work on the synthesis and biological activity evaluation of flavonoids [21–22], we report here a convenient and efficient method for mono-, di- and tri-*O*-methylation of kaempferol, by taking the advantage of the different reactive potential (hydrolysis, benzylation and methylation) of *O*-acetoxy groups in multi-acetylated kaempferol. The major strategy depends on the selective benzylation and controllable deacetylation of kaempferol acetates.

## Results and Discussion

### Selectivity of direct methylation of kaempferol

Kaempferol is both significantly similar to and different from quercetin. Rao et al. found that the methylation of the hydroxy groups in quercetin occurs gradually following a specific sequential position order: 4′ > 7 > 3 > 3′ > 5 [23]. Recently, however, Roland ascertained that the relative reactivity order of OH in quercetin presents an invert sequence: 7 > 3 ≈ 4′ [19]. And this sequence varies with different *O*-substituted functions [24].

We first investigated the direct methylation of kaempferol in order to get an insight into the reactivity of the hydroxy groups. Dimethyl sulfate was chosen as methylating reagent. As illustrated in Table 1, with 1 equiv of dimethyl sulfate and 1.2 equiv of potassium carbonate at room temperature for 12 h or more, three mono-*O*-methylkaempferols (3-, 4′- and 7-*O*-methylkaempferol) were obtained in only 34% isolated yield. Using 2 equiv of dimethyl sulfate at the same conditions, mono-, di- and a trace of tri-*O*-methylated kaempferols were isolated using silica gel column chromatography followed by reversed phase HPLC. Tetra-*O*-methylkaempferol was obtained quantitatively when dimethyl sulfate was increased to 6 equiv at 30 °C for 24 h (Table 1).

**Table 1:** Direct methylation of kaempferol in acetone (yield, %).

	1 equiv^a^, rt	2 equiv^a^, rt	6 equiv^a^, 30 °C

kaempferol	62	4	0
3-, 4′-, 7-mono-*O*-methylkaempferol	6, 11, 17	0, 12, 19	0
3,7-, 3,4′-, 4′,7-di-*O*-methylkaempferol	0	16, 10, 29	0
3,4′,7-tri-*O*-methylkaempferol	0	4	0
3,4′,5,7-tetra-*O*-methylkaempferol	0	0	96

^a^1 equiv is defined as the stoichiometric amount of Me_2_SO_4_ in the presence of 1.2 equiv of K_2_CO_3_.

These results indicated a reactivity order of each hydroxy group of kaempferol: 7 > 4′ > 3 >> 5, different from that of quercetin: 3 ≈ 4′ [19]. The 7-OH of kaempferol should be the most highly reactive hydroxy group in nucleophilic alkylation, followed by the 4'-OH. The 5-OH resists to alkylation because of the strong intramolecular H-bond with the 4-carbonyl group.

### Synthesis of 7-*O*-methylkaempferol (**4**), 4′,7-di-*O*-methylkaempferol (**5**) and 3,7-di-*O*-methylkaempferol (**7**)

**Scheme 1:**
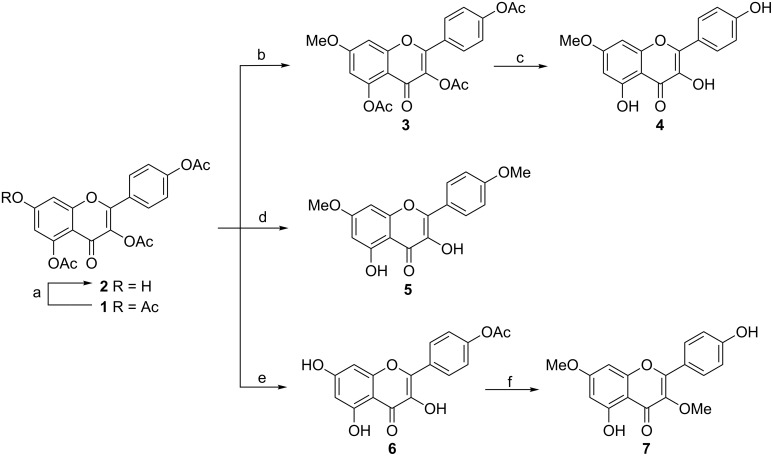
Reagents and conditions: (a) PhSH, imidazole, NMP (*N*-methylpyrrolidinone), 0 °C, 91%; (b) Me_2_SO_4_ (1.3 equiv), K_2_CO_3_, acetone, rt, 87%; (c) NH_3_ (g), MeOH, 97%; (d) Me_2_SO_4_ (3 equiv), K_2_CO_3_, acetone, MeOH, reflux, 90%; (e) i: AlCl_3_, CH_2_Cl_2_, CH_3_CN, reflux, ii: 1 M HCl (aq), 89%; (f) i: Me_2_SO_4_ (2.6 equiv), K_2_CO_3_, acetone, rt, ii: NH_3_ (g), MeOH, 84%.

Kaempferol was acetylated to furnish the peracetylated derivative **1**. Jurd found that with excess of methyl iodide and potassium carbonate in dry acetone, only the 7-*O*-acetyl group of quercetin pentaacetate is replaced [25]. For the synthesis of 7-*O*-methylkaempferol (**4**), we first adopted Jurd’s method. Under similar conditions, however, a mixture of 7-*O*-, 4′-*O*- and 4′,7-di-*O*-methylkaempferol was obtained whether methyl iodide or dimethyl sulfate was used as methylating reagent. Separation of 7- and 4′-mono isomers from the crude product involved tedious and laborious work-up. We turned to employ thiophenol with imidazole in *N*-methylpyrrolidinone (NMP) at 0 °C [26], for selective removal of the most electrophilic 7-*O*-acetyl group from the fully acetylated kaempferol. 3,4′,5-Tri-*O*-acetylkaempferol (**2**) was obtained in excellent yield (91%). Methylation of **2** with 1.3 equiv of dimethyl sulfate at room temperature generated **3** in a yield of 87%, which was further treated with methanolic ammonia (7.0 M) for deacetylation to afford compound **4** (Scheme 1).

The location of the methoxy group in **4** was unambiguously determined by the NOESY correlations of OCH_3_ at δ_H_ 3.86 to 8-H (δ_H_ 6.75) and 6-H (δ_H_ 6.35). Three characteristic singlets at δ_H_ 12.47, 10.14 and 9.51 were thus assigned separately to 5-, 4′- and 3-OH based on NMR as well as HMBC experiments (see Supporting Information File 1 for experimental and spectra data), different from the tentative assigment [27–28].

Treatment of **2** with an excess of dimethyl sulfate (3 equiv) and K_2_CO_3_ (3.6 equiv) in acetone/MeOH (3:1) under reflux, unexpectedly, leads to the formation of 4′,7-di-*O*-methylkaempferol (**5**) in 90% yield (Scheme 1). We speculated that this was due to the OH reactivity (7 > 4′ > 3 >> 5), and the hydrolysis rate of OAc in a reversed order (5 > 4′ > 3) in kaempferol. Deacetylation of 4′-OAc in **2** and methylation of thus derived 4′-OH were accomplished simultaneously in one pot. Caution should be taken to the ratio of acetone to MeOH and the amount of K_2_CO_3_, since high polarity of solvent or excess of base result in heterocycle cleavage of the flavonoid skeleton.

To obtain 3,7-di-*O*-methylkaempferol (**7**), we envisioned the possibility of methylation from the 4′-*O*-acetylkaempferol (**6**), which can be derived from **2**. We were delighted that the combination of reflux with aluminum chloride in polar solvent CH_3_CN and then hydrolysis in 1 M HCl (aq) at room temperature was an excellent method for selective removal of the 3- and 5-*O*-acetyl groups from triacetylated kaempferol (**2**). No side products were detected by TLC analysis. With other Lewis acids such as ZnCl_2_, the result was very different. The desired product **6** was obtained with reproducible yield (89%) after chromatographic separation. Compound **7** was thus prepared by methylation of **6** and subsequent hydrolysis at appropriate conditions in good yield (Scheme 1).

### Synthesis of 3-*O*-methylkaempferol (**12**), 5,4′-di-*O*-methylkaempferol (**16**) and 4′-*O*-methylkaempferol (**18**)

**Scheme 2:**
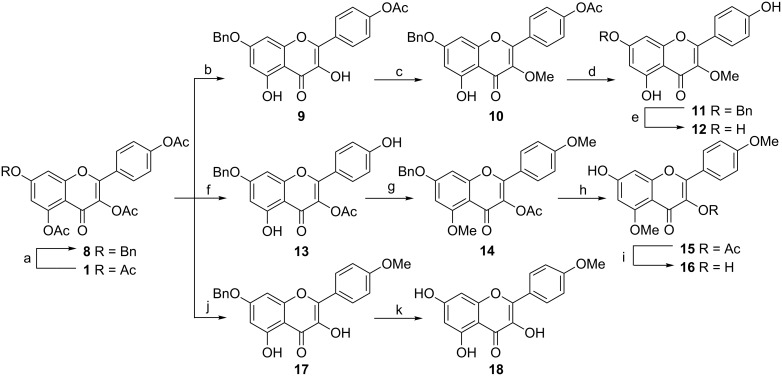
Reagents and conditions: (a) BnBr (1.9 equiv), KI, K_2_CO_3_, acetone, 85%; (b) i: AlCl_3_, CH_2_Cl_2_, CH_3_CN, reflux, ii: 1 M HCl (aq), 86%; (c) Me_2_SO_4_ (1.3 equiv), K_2_CO_3_, acetone, rt, 85%; (d) NH_3_ (g), MeOH, 96%; (e) Pd/C, H_2_, 91%; (f) MeOH, acetone, 1 M HCl (aq), reflux, 82%; (g) Me_2_SO_4_ (3.3 equiv), K_2_CO_3_, acetone, 30 °C, 92%; (h) Pd/C, H_2_, 92%; (i) NH_3_ (g), MeOH, 94%; (j) Me_2_SO_4_ (1.5 equiv), K_2_CO_3_, acetone, MeOH, reflux, 82%; (k) Pd/C, H_2_, 93%.

For the synthesis of 3-*O*-methylkaempferol (**12**), we decided to selectively protect the 7-OH with benzyl, in order to make the methylation of 3-OH proceed smoothly. Previous studies have revealed that benzylation of quercetin pentacetate in anhydrous acetone and subsequent alkaline hydrolysis gives a mixture of 7-*O*- and 4',7-di-*O*-benzylquercetin [29]. Using this strategy, peracetylated derivative **1** was treated with an excess of benzyl bromide and catalytic amounts of potassium iodide in the presence of K_2_CO_3_ under reflux for 24 h. Fortunately, only **8** was obtained by crystallization from absolute ethanol in an 85% yield (Scheme 2).

With **8** in hand, the synthesis of **12** is based on similar synthetic strategy to that of **7** from **2**. The key step was the selective release of the 3-OH and 5-OH from compound **8**, using the combination of AlCl_3_ and HCl (aq) (Scheme 2).

Methylation of **9** with 1.3 equiv of dimethyl sulfate at room temperature provided **10** with a yield of 85%. The final deprotection of **10** involving deacetylation with 7.0 M methanolic ammonia and debenzylation by hydrogenolysis with 10% palladium on carbon in EtOAc/MeOH (1:3) at room temperature afforded **12** (Scheme 2).

In order to prepare 4′,5-di-*O*-methylkaempferol (**16**), selective deprotection of 4′- and 5-*O*-acetyl groups in compound **8** was necessary. We first employed acetone/MeOH or THF/MeOH system with K_2_CO_3_ or Na_2_CO_3_ as base, but failed. We found that under acidic conditions the 4′- and 5-*O*-acetyl groups could be removed selectively. The best result was obtained using a mixture of 1 M HCl (aq)/acetone/MeOH (3:3:1) at reflux for 3.5 h. Partial deacetylated product **13** was thus acquired in 82% isolated yield. Next, in order to methylate the less reactive 5-OH, we applied an intermittent injection method with a higher concentration of dimethyl sulfate. After 24 h of total reaction time at 30 °C, **14** was obtained in 92% yield. The following step of this synthesis was the deprotection of the benzyl and acetyl groups (Scheme 2).

Two different methods are available for the preparation of 4′-*O*-methylkaempferol (**18**): methylation of **13** with 1.3 equiv of dimethyl sulfate and subsequent hydrolysis at appropriate conditions, or deacetylation of **8** followed methylation successively as described for **5**. Obviously, the latter route is more convenient. As may be expected, treatment of 7-*O*-benzyl triacetate **8** with excess of dimethyl sulfate and K_2_CO_3_ in acetone/MeOH at reflux afforded **17** in high yield (82%). Subsequent hydrogenolysis of **17** catalyzed by 10% palladium on carbon in EtOAc/MeOH (1:3) at room temperature provided **18** (Scheme 2).

### Synthesis of 3,4′-di-*O*-methylkaempferol (**22**) and 3,4′,5,-tri-*O*-methylkaempferol (**23**)

**Scheme 3:**
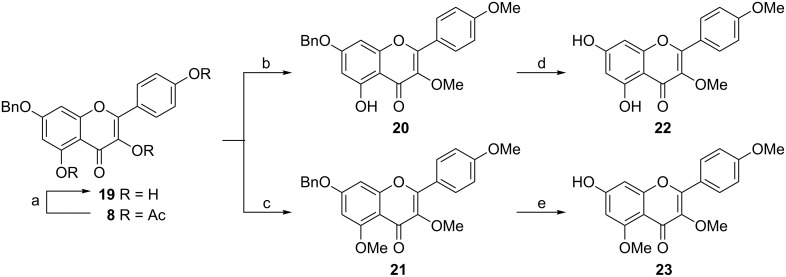
Reagents and conditions: (a) NH_3_ (g), MeOH, 95%; (b) Me_2_SO_4_ (2.6 equiv), K_2_CO_3_, acetone, rt, 87%; (c) Me_2_SO_4_ (4.6 equiv), K_2_CO_3_, acetone, 30 °C, 95%; (d) Pd/C, H_2_, 91%; (e) Pd/C, H_2_, 89%.

3,4′-Di-*O*-methylkaempferol (**22**) and 3,4′,5-tri-*O*-methylkaempferol (**23**) were prepared from **19** derived from **8**. Controlled di-*O*-methylation and tri-*O*-methylation of **19** were achieved by the quantity of Me_2_SO_4_, resulting in **20** and **21**, respectively. Hydrogenolysis of **20** and **21** afforded **22** and **23** (Scheme 3).

All the synthetic compounds including eleven unreported intermediates were characterized by the IR, ^1^H NMR, ^13^C NMR and ESI-HRMS spectral data. Among them, eight title compounds and two intermediates (**9** and **13**) with methoxy or acetoxy group(s) at the desired position(s) were further confirmed by HMBC and/or NOESY experiments, and the ^1^H and ^13^C NMR signals were explicitly assigned (see Supporting Information File 1 for experimental and spectra data).

## Conclusion

A convenient and efficient methodology for the selective *O*-methylation of kaempferol was developed. This strategy mainly relies on the selective benzylation and controllable hydrolysis of multiple *O*-acetylated kaempferols. Eight mono-, di- and tri-*O*-methylated kaempferols were thus prepared with excellent yields. The reported protocol could be extended for the selective derivatization of polyhydroxylated natural products like polyketides or phenylpropanoids.

## Supporting Information

File 1 Experimental section; NMR and ESI-HRMS spectra of **1–23**; NOESY spectra of **4** and **13**; HMBC spectra of **4**, **5**, **7**, **9**, **12**, **13**, **16**, **18**, **22**, **23**.
