# Supplementary material for: Selective methylation of kaempferol via benzylation and deacetylation of kaempferol acetates
Source: Beilstein J Org Chem. 2015 Feb 25;11:288–93. doi: 10.3762/bjoc.11.33 (PMC4361994; doi:10.3762/bjoc.11.33)

# Supporting Information

## for

# Selective methylation of kaempferol via benzylation and deacetylation of kaempferol acetates

Qinggang Mei[1,2,3], Chun Wang[2], Weicheng Yuan[1] and Guolin Zhang*[2]

Address: [1]Chengdu Institute of Organic Chemistry, Chinese Academy of Sciences, Chengdu 610041, China, [2]Chengdu Institute of Biology, Chinese Academy of Sciences, Chengdu 610041, China and [3]University of Chinese Academy of Sciences, Beijing 100049, China

Email: Guolin Zhang* - zhanggl@cib.ac.cn

* Corresponding author

Experimental section; NMR and ESI-HRMS spectra of **1–23**; NOESY spectra of **4** and **13**; HMBC spectra of **4**, **5**, **7**, **9**, **12**, **13**, **16**, **18**, **22** and **23**


| Contents | Page No. |
|---|---|
| Experimental section | S1–S16 |
| $^{1}H$ NMR Spectra of **1–23** | S17–S39 |
| $^{13}C$ NMR Spectra of **1–23** | S40–S62 |
| ESI-HRMS Spectra of **1–23** | S63–S85 |
| NOESY Spectra of **4** and **13** | S86–S87 |
| HMBC Spectra of **4**, **5**, **7**, **9**, **12**, **13**, **16**, **18**, **22** and **23** | S88–S97 |

# Experimental section

Melting points were determined with an X-6 apparatus and are uncorrected. IR spectra were recorded on a Perkin-Elmer Spectrum One spectrometer in KBr disks. NMR spectra, including NOESY and $^{1}H$-$^{13}C$ HMBC experiments, were carried out at Bruker AC-400 (400 MHz for $^{1}H$ NMR and 100 MHz for $^{13}C$ NMR) or Bruker AM-600 (600 MHz for $^{1}H$ NMR and 150 MHz for $^{13}C$ NMR) spectrometers in $CDCl_3$ or in DMSO-$d_6$ using TMS as internal reference at ambient temperature; chemical shifts (δ) and coupling constants (*J*) are given in ppm and Hz, respectively. ESI-HRMS spectra were obtained on a Bruker Bio TOF IIIQ (quadrupole time of flight) mass spectrometer. Analytical TLC was performed on plates precoated with 0.15–0.2 mm of silica gel $GF_{254}$ from QOCEC (Qingdao Ocean Chemical Engineering Company, P. R. China). HPLC was equipped with Perkin-Elmer series 200 pump, Perkin-Elmer series UV/Vis detector, 200 µL manual injector, and Phenomenex $C_{18}$ column (10 mm × 250 mm, 10 µm). Reagents were analytical reagent grade and were used without further purification unless otherwise noted. $CH_2Cl_2$ and NMP were freshly distilled over $CaH_2$.

**3,4′,5,7-Tetra-*O*-acetylkaempferol (1)** [1]

Acetic anhydride (60 mL) was added to the solution of dry kaempferol (4 g, 13.99 mmol) in pyridine (30 mL), and the mixture was allowed to stand overnight at ambient temperature. After complete conversion monitored by TLC, the solvent was removed and the residue was poured into crushed ice with vigorous stirring. The abundant resulting off-white precipitate was recovered by filtration and washed with cold water and then methanol, and dried in air. The crude product was crystallized from acetone/95% ethanol (1:3) to afford **1** (5.95 g, 94%) as colorless needles, mp: 183-184 ºC. IR ($cm^{-1}$): 1775, 1661, 1631, 1503, 1476, 1435, 1370, 1186, 1158, 1122,

1081, 1013.$^1$H NMR (600 MHz, $CDCl_3$): δ = 7.84 (d, *J* = 8.7 Hz, 2H), 7.32 (d, *J* = 2.1 Hz, 1H), 7.26 (d, *J* = 8.7 Hz, 2H), 6.87 (d, *J* = 2.1 Hz, 1H), 2.43 (s, 3H), 2.34 (s, 6H), 2.32 (s, 3H). $^{13}$C NMR (150 MHz, $CDCl_3$): δ = 170.24, 169.39, 168.99, 167.98, 167.91, 157.10, 154.91, 154.38, 153.08, 150.61, 134.08, 129.76, 127.16, 122.21, 114.97, 113.97, 109.10, 21.30, 21.18, 20.71. ESI-HRMS: 477.0807 $[M+Na]^+$ (calcd. for $C_{23}H_{18}O_{10}Na$: 477.0792).

**3,4′,5-Tri-*O*-acetylkaempferol (2)**

This was carried out according to the reported procedure [2]. To a solution of kaempferol tetraacetate (**1**) (1g, 2.2 mmol) in NMP (5 mL) was added imidazole (60 mg, 0.88 mmol) followed by thiophenol (0.31 mL, 3.08 mmol) at 0 ºC. The mixture was stirred at 0 ºC for 11 h and then diluted with EtOAc (100 mL), and washed successively with 1 M HCl (aq) (50 mL), water (40 mL) and brine (40 mL). The organic layer was dried over $MgSO_4$ and concentrated under reduced pressure. The residue was purified over silica gel column using acetone/petroleum ether (1:2) as solvents to give **2** (825 mg, 91%) as white powder, mp: 136-138 ºC. IR ($cm^{-1}$): 3348, 2935, 1765, 1728, 1636, 1501, 1453, 1371, 1249, 1201, 1179, 1077. $^1$H NMR (400 MHz, DMSO-$d_6$): δ = 11.32 (s, 1H), 7.91 (d, *J* = 8.8 Hz, 2H), 7.37 (d, *J* = 8.8 Hz, 2H), 6.93 (d, *J* = 2.3 Hz, 1H), 6.64 (d, *J* = 2.3 Hz, 1H), 2.32 (s, 3H), 2.30 (s, 6H). $^{13}$C NMR (100 MHz, DMSO-$d_6$): δ = 169.00, 168.99, 168.79, 167.99, 162.78, 157.61, 153.30, 152.61, 150.22, 132.61, 129.48, 126.64, 122.63, 109.26, 109.04, 100.96, 20.91, 20.90, 20.32. ESI-HRMS: 435.0695 $[M+Na]^+$ (calcd. for $C_{21}H_{16}O_9Na$: 435.0687).

**7-*O*-Methyl-3,4′,5-tri-*O*-acetylkaempferol (3)** [3]

A suspension of compound **2** (200 mg, 0.49 mmol), anhydrous $K_2CO_3$ (108 mg, 0.78 mmol) and $Me_2SO_4$ (0.061 mL, 0.64 mmol) in dry acetone (6 mL) was stirred at room

temperature for 12 h. The reaction mixture was diluted with water (10 mL) and extracted with EtOAc (20 mL). The organic layer was washed with brine, dried over $MgSO_4$ and concentrated under reduced pressure. The dried residue was purified over silica gel column using acetone/petroleum ether (1:4) as solvents to provide **3** (180 mg, 87%) as white powder, mp: 186-187 ºC. IR ($cm^{-1}$): 2960, 2902, 1763, 1658, 1600, 1494, 1368, 1290, 1202, 1155, 1014. $^1$H NMR (400 MHz, $CDCl_3$): δ = 7.84 (d, *J* = 8.8 Hz, 2H), 7.25 (d, *J* = 8.8 Hz, 2H), 6.84 (d, *J* = 2.4 Hz, 1H), 6.64 (d, *J* = 2.4 Hz, 1H), 3.91 (s, 3H), 2.43 (s, 3H), 2.34 (s, 3H), 2.32 (s, 3H). $^{13}$C NMR (150 MHz, $CDCl_3$): δ = 170.27, 169.72, 169.06, 168.15, 163.98, 158.36, 154.30, 152.88, 150.92, 133.87, 129.68, 127.48, 122.15, 111.27, 108.85, 99.03, 56.22, 21.31, 21.27, 20.77. ESI-HRMS: 449.0850 $[M+Na]^+$ (calcd. for $C_{22}H_{18}O_9Na$: 449.0843).

**7-*O*-Methylkaempferol (rhamnocitrin) (4)** [4]

The solution of compound **3** (150 mg, 0.35 mmol) in 7.0 M methaniloc ammonia (2 mL) was stirred at room temperature for 3 h. The solution was diluted with MeOH (4 mL) and neutralized cautiously with 0.5 M HCl (aq) and then left at room temperature for 12 h. The abundant resulting precipitate was recovered by filtration, washed with water and dried over $P_2O_5$ under vacuum to give **4** (102 mg, 97%) as yellow powder, mp: 172-173 ºC. IR ($cm^{-1}$): 3481, 3274, 2926, 2854, 1609, 1587, 1503, 1415, 1355, 1231, 1164. H NMR (400 MHz, DMSO-$d_6$): δ = 12.47 (s, 1H, OH-5), 10.14 (s, 1H, OH-4′), 9.51 (s, 1H, OH-3), 8.08 (d, *J* = 8.8 Hz, 2H, H-2′,6′), 6.93 (d, *J* = 8.8 Hz, 2H, H-3′,5′), 6.75 (d, *J* = 2.0 Hz, 1H, H-8), 6.35 (d, *J* = 2.0 Hz, 1H, H-6), 3.86 (s, 3H, $OCH_3$-7). $^{13}$C NMR (150 MHz, DMSO-$d_6$): δ = 176.04 (C-4), 164.91 (C-7), 160.38 (C-5), 159.32 (C-4′), 156.11 (C-9), 147.28 (C-2), 135.96 (C-3), 129.58 (C-2′,6′), 121.58 (C-1′), 115.46 (C-3′,5′), 104.04 (C-10), 97.46 (C-6), 92.03 (C-8), 56.02 ($OCH_3$-7). ESI-HRMS: 299.0568 $[M-H]^-$ (calcd. for $C_{16}H_{11}O_6$: 299.0561).

**4′,7,-Di-*O*-methylkaempferol (5)** [5]

A mixture of compound **2** (166 mg, 0.4 mmol), dimethyl sulfate (0.12 mL, 1.25 mmol), anhydrous $K_2CO_3$ (199 mg, 1.44 mmol) and acetone (6 mL) was refluxed for 2 h. Then methanol (2 mL) was added to the mixture and the reaction continued for 24 h. Excess of solvent was removed under reduced pressure and ice water (5 mL) was poured onto the residue with stirring. The mixture was acidified cautiously with 0.5 M HCl (aq) until pH = 5 and extracted with $CHCl_3$ (8 mL). The organic extract was washed with saturated $NaHCO_3$ solution, brine and dried over $Mg_2SO_4$, filtered and evaporated under reduced pressure. Crystallization of the dried residue from acetone/petroleum (1:4) ether afforded **5** (113 mg, 90%) as yellow needles, mp: 157-158 ºC. IR ($cm^{-1}$): 3317, 2926, 2851, 1660, 1596, 1506, 1311, 1260, 1167, 1031. $^1H$ NMR (400 MHz, DMSO-$d_6$): δ = 12.44 (s, 1H, OH-5), 9.67 (s, 1H, OH-3), 8.18 (d, *J* = 9.1 Hz, 2H, H-2′,6′), 7.13 (d, *J* = 9.1 Hz, 2H, H-3′,5′), 6.78 (d, *J* = 2.2 Hz, 1H, H-8), 6.37 (d, *J* = 2.2 Hz, 1H, H-6), 3.87 (s, 3H, $OCH_3$-7), 3.85 (s, 3H, $OCH_3$-4′). $^{13}C$ NMR (150 MHz, DMSO-$d_6$): δ = 176.13 (C-4), 164.98 (C-7), 160.59 (C-4′), 160.37 (C-5), 156.15 (C-9), 146.69 (C-2), 136.35 (C-3), 129.38 (C-2′,6′), 123.15 (C-1′), 114.04 (C-3′,5′), 104.08 (C-10), 97.49 (C-6), 92.06 (C-8), 56.02 ($OCH_3$-7), 55.37 ($OCH_3$-4′). ESI-HRMS: 315.0873 $[M+H]^+$ (calcd. for $C_{17}H_{15}O_6$: 315.0863).

**4′-*O*-Acetylkaempferol (6)**

To a solution of compound **2** (251 mg, 0.61 mmol) in $CH_2Cl_2$ (1 mL) and $CH_3CN$ (3 mL) was added anhydrous $AlCl_3$ (201 mg, 1.52 mmol). The mixture was refluxed for 3 h. After cooling down to room temperature, 1 M HCl (aq) (3 mL) was added and the mixture was stirred further for 1 h. The reaction mixture was diluted with $CHCl_3$ and the pH was adjusted to 4−5 with saturated $NaHCO_3$ solution. After evaporation of the

organic layer, the amorphous residue was purified over silica gel column using EtOAc/$CH_2Cl_2$/petroleum ether (1:3:6) as solvents to yield **5** (178 mg, 89%) as yellow needles, mp: 164-165 ºC. IR ($cm^{-1}$): 3403, 3298, 2928, 1722, 1653, 1623, 1603, 1504, 1370, 1313, 1245, 1168. $^1$H NMR (400 MHz, DMSO-$d_6$): δ = 12.36 (s, 1H), 10.87 (s, 1H), 9.76 (s, 1H), 8.20 (d, *J* = 8.9 Hz, 2H), 7.33 (d, *J* = 2.0 Hz, 2H), 6.47 (d, *J* = 2.0 Hz, 1H), 6.22 (d, *J* = 2.0 Hz, 1H), 2.31 (s, 3H). $^{13}$C NMR (100 MHz, DMSO-$d_6$): δ = 176.30, 169.09, 164.28, 160.80, 156.41, 151.43, 145.21, 137.07, 128.97, 128.53, 122.11, 103.27, 98.38, 93.64, 20.92. ESI-HRMS: 327.0510 $[M-H]^-$ (calcd. for $C_{17}H_{11}O_7$: 327.0510).

**3,7-Di-*O*-methylkaempferol (kumatakenin) (7)** [6]

Methylation of compound **6** (151 mg, 0.46 mmol) with dimethyl sulfate (0.115 mL, 1.2 mmol) and anhydrous $K_2CO_3$ (190 mg, 1.38 mmol) in dry acetone (6 mL) was carried out at room temperature for 12 h**.** The mixture was diluted with water (15 mL) and extracted with EtOAc (20 mL). The organic layer was washed with brine, dried over $MgSO_4$ and concentrated under reduced pressure. Without purification, the dried residue was stirred with 7.0 M methanolic ammonia (3 mL) for 3 h. The precipitate formed was filtered off and crystallized from acetone/petroleum ether (1:2) to provide **7** (121 mg, 84%) as yellow crystals, mp: 228-230 ºC. IR ($cm^{-1}$): 3410, 3261, 2928, 2854, 1658, 1602, 1498, 1346, 1287, 1170. $^1$H NMR (400 MHz, DMSO-$d_6$): δ = 12.67 (s, 1H, OH-5), 10.32 (s, 1H, OH-4′), 7.98 (d, *J* = 8.9 Hz, 2H, H-2′,6′), 6.95 (d, *J* = 8.9 Hz, 2H, H-3′,5′), 6.75 (d, *J* = 2.0 Hz, 1H, H-8), 6.37 (d, *J* = 2.0 Hz, 1H, H-6), 3.86 (s, 3H, $OCH_3$-7), 3.80 (s, 3H, $OCH_3$-3). $^{13}$C NMR (100 MHz, DMSO-$d_6$): δ = 178.11 (C-4), 165.16 (C-7), 160.96 (C-5), 160.32 (C-4′), 156.34 (C-9), 155.99 (C-2), 137.86 (C-3), 130.25 (C-2′,6′), 120.50 (C-1′), 115.70 (C-3′,5′), 105.22 (C-10), 97.81 (C-6), 92.39 (C-8), 59.74 ($OCH_3$-3), 56.12 ($OCH_3$-7). ESI-HRMS: 315.0862 $[M+H]^+$ (calcd. for

$C_{17}H_{15}O_6$: 315.0863).

**7-*O*-Benzyl-3,4′,5-tri-*O*-acetylkaempferol (8)**

A mixture of kaempferol tetraacetate (**1**) (2.5 g, 5.51 mmol), potassium iodide (450 mg, 2.71 mmol), benzyl bromide (1.25 mL, 10.59 mmol), anhydrous $K_2CO_3$ (1925 mg, 13.95 mmol) and dry acetone (80 mL) was refluxed for 24 h. The filtered solution was concentrated to give an amorphous solid, which was crystallized from absolute ethanol to give **8** as white crystals (2.35 g, 85%), mp: 163-164 $^oC$. IR ($cm^{-1}$): 1771, 1636, 1621, 1505, 1442, 1369, 1290, 1193, 1170, 1077, 1017. $^1H$ NMR (600 MHz, $CDCl_3$): δ = 7.83 (d, *J* = 8.8 Hz, 2H), 7.43–7.35 (m, 5H), 7.24 (d, *J* = 8.8 Hz, 2H), 6.90 (d, *J* = 2.3 Hz, 1H), 6.71 (d, *J* = 2.3 Hz, 1H), 5.16 (s, 2H), 2.43 (s, 3H), 2.34 (s, 3H), 2.31 (s, 3H). $^{13}C$ NMR (150 MHz, $CDCl_3$): δ = 170.23, 169.70, 169.05, 168.13, 163.02, 158.30, 154.33, 152.88, 150.96, 135.37, 133.88, 129.69, 128.99, 128.74, 127.70, 127.46, 122.15, 111.45, 109.41, 100.00, 71.01, 21.31, 21.28, 20.76. ESI-HRMS: 525.1152 $[M+Na]^+$ (calcd. for $C_{28}H_{22}O_9Na$: 525.1156).

**7-*O*-Benzyl-4′-*O*-acetylkaempferol (9)**

To a solution of compound **8** (300 mg, 0.6 mmol) in $CH_2Cl_2$ (1 mL) and $CH_3CN$ (3 mL) was added anhydrous $AlCl_3$ (201 mg, 1.52 mmol). The mixture was refluxed for 3 h. After cooling down to room temperature, 1 M HCl (aq) (3 mL) was added and the mixture was stirred further for 1 h. The reaction mixture was diluted with $CHCl_3$ and the pH was adjusted to 4−5 with saturated $NaHCO_3$ solution. After evaporation of the organic layer, the residue was purified over silica gel column using acetone/petroleum ether (1:4) as solvents to yield compound **9** (216 mg, 86%) as pale yellow powder, mp: 172-173 $^oC$. IR ($cm^{-1}$): 3324, 2925, 2856, 1756, 1647, 1623, 1598, 1503, 1368, 1308,1212, 1171. $^1H$ NMR (400 MHz, DMSO-$d_6$): δ = 12.35 (s, 1H, OH-5), 9.91 (s, 1H,

OH-3), 8.23 (d, *J* = 8.9 Hz, 2H, H-2′,6′), 7.48 (d, *J* = 7.3 Hz, 2H, H-2″,6″), 7.42 (t, *J* = 7.3 Hz, 2H, H-3″,5″), 7.36 (overlapped, 3H, H-4″,3′,5′), 6.89 (d, *J* = 2.1 Hz, 1H, H-8), 6.47 (d, *J* = 2.1 Hz, 1H, H-6), 5.24 (s, 2H, H-7″), 2.31 (s, 3H, $CH_3COO$-4′). $^{13}C$ NMR (100 MHz, DMSO-$d_6$): δ = 176.45 (C-4), 169.04 ($CH_3$*C*OO-4′), 164.21 (C-7), 160.48 (C-5), 156.27 (C-9), 151.53 (C-4′), 145.65 (C-2), 137.40 (C-3), 136.13 (C-1″), 128.99 (C-2′,6′), 128.56 (C-3″,5″), 128.43 (C-1′), 128.16 (C-4″), 127.88 (C-2″,6″), 122.13 (C-3′,5′), 104.37 (C-10), 98.20 (C-6), 93.06 (C-8), 70.02 (C-7″), 20.90 (*C*$H_3$COO-4′). ESI-HRMS: 419.1115 $[M+H]^+$ (calcd. for $C_{24}H_{19}O_7$: 419.1125).

**3-*O*-Methyl-7-*O*-benzyl-4′-*O*-acetylkaempferol (10)**

A suspension of compound **9** (201 mg, 0.48 mmol), anhydrous $K_2CO_3$ (105 mg, 0.76 mmol) and $Me_2SO_4$ (0.06 mL, 0.63 mmol) in dry acetone (6 mL) was stirred at room temperature for 12 h. The mixture was diluted with water (10 mL), and extracted with EtOAc (20 mL). The organic layer was washed with brine, dried over $MgSO_4$ and then concentrated. The residue was crystallized from acetone/petroleum ether (1:3) to afford **10** (176 mg, 85%) as yellowish-brown crystals, mp: 115-117 ºC. IR ($cm^{-1}$): 3444, 2925, 2853, 1744, 1661, 1609, 1503, 1461, 1384, 1342, 1230, 1171, 1005. $^1H$ NMR (400 MHz, DMSO-$d_6$): δ = 12.53 (s, 1H), 8.09 (d, *J* = 8.7 Hz, 2H), 7.47 (d, *J* = 7.4 Hz, 2H), 7.41 (t, *J* = 7.4 Hz, 2H), 7.35 (t, *J* = 8.5 Hz, 3H), 6.86 (d, *J* = 1.9 Hz, 1H), 6.48 (d, *J* = 1.9 Hz, 1H), 5.23 (s, 2H), 3.84 (s, 3H), 2.32 (s, 3H). $^{13}C$ NMR (100 MHz, DMSO-$d_6$): δ = 178.31, 169.02, 164.36, 161.02, 156.46, 154.86, 152.42, 139.01, 136.09, 129.75, 128.57, 128.18, 127.87, 127.49, 122.34, 105.61, 98.60, 93.40, 70.06, 60.08, 20.92. ESI-HRMS: 433.1261 $[M+H]^+$ (calcd. for $C_{25}H_{21}O_7$: 433.1282).

**3-*O*-Methyl-7-*O*-benzylkaempferol (11)**

A solution of compound **10** (152 mg, 0.35 mmol) in 7.0 M methanolic ammonia (3 mL)

was stirred at room temperature for 3 h. The precipitate formed was filtered off and crystallized from acetone/petroleum ether (1:3) to give **11** (132 mg, 96%) as yellow needles, mp: 155-157 ºC. IR ($cm^{-1}$): 3421, 2930, 1603, 1494, 1371, 1339, 1287, 1220, 1172. $^{1}H$ NMR (400 MHz, DMSO-$d_6$): δ = 12.67 (s, 1H), 10.30 (s, 1H), 7.97 (d, *J* = 8.9 Hz, 2H), 7.47 (d, *J* = 7.1 Hz, 2H), 7.41 (t, *J* = 7.1 Hz, 2H), 7.35 (t, *J* = 7.1 Hz, 1H), 6.96 (d, *J* = 8.9 Hz, 2H), 6.84 (d, *J* = 2.2 Hz, 1H), 6.46 (d, *J* = 2.2 Hz, 1H), 5.23 (s, 2H), 3.80 (s, 3H). $^{13}C$ NMR (100 MHz, DMSO-$d_6$): δ = 178.05, 164.09, 160.97, 160.30, 156.24, 156.00, 137.85, 136.12, 130.20, 128.54, 128.13, 127.84, 120.46, 115.68, 105.32, 98.39, 93.23, 69.98, 59.70. ESI-HRMS: 389.1036 $[M-H]^-$ (calcd. for $C_{23}H_{17}O_6$: 389.1031).

**3-*O*-Methylkaempferol (12)** [7]

A mixture of **11** (90 mg, 0.23 mmol), 10% Pd/C (10 mg), MeOH (9 mL) and EtOAc (3 mL) was stirred at ambient temperature under a hydrogen balloon for 5 h. The resulting mixture was filtered off through celite eluting with EtOAc (3 mL). The filtrate was concentrated to give the crude product, which was crystallized from acetone/petroleum ether (1:2) to afford **12** (63 mg, 91%) as pale yellow needles, mp: 237-239 ºC. IR ($cm^{-1}$): 3502, 3118, 2925, 2855, 1467, 1606, 1489, 1360, 1308, 1224, 1161. $^{1}H$ NMR (400 MHz, DMSO-$d_6$): δ = 12.69 (s, 1H, OH-5), 10.85 (s, 1H, OH-7), 10.27 (s, 1H, OH-4′), 7.94 (d, *J* = 8.9 Hz, 2H, H-2′,6′), 6.94 (d, *J* = 8.9 Hz, 2H, H-3′,5′), 6.44 (d, *J* = 2.0 Hz, 1H, H-8), 6.20 (d, *J* = 2.0 Hz, 1H, H-6), 3.78 (s, 3H, $OCH_3$-3). $^{13}C$ NMR (150 MHz, DMSO-$d_6$): δ = 177.89 (C-4), 164.10 (C-7), 161.23 (C-5), 160.14 (C-4′), 156.36 (C-9), 155.60 (C-2), 137.59 (C-3), 130.11 (C-2′,6′), 120.55 (C-1′), 115.63 (C-3′,5′), 104.20 (C-10), 98.55 (C-6), 93.69 (C-8), 59.68 ($OCH_3$-3). ESI-HRMS: 299.0563 $[M-H]^-$ (calcd. for $C_{16}H_{11}O_6$: 299.0561).

**3-*O*-Acetyl-7-*O*-benzylkaempferol (13)**

To the solution of compound **8** (300 mg, 0.6 mmol) in acetone (3 mL) and MeOH (1 mL) was added 1 M HCl (aq) (3 mL). The mixture was refluxed for 3.5 h, and then diluted with $CHCl_3$ (5 mL), neutralized with aqueous $NaHCO_3$, and extracted with $CHCl_3$. After evaporation of the organic layer, the dried residue was purified over silica gel column using acetone/petroleum ether (1:3) as solvents to provide **13** (205 mg, 82%) as yellow needles, mp: 170-171 $^oC$. IR ($cm^{-1}$): 3313, 2935, 2876, 1781, 1655, 1603, 1583, 1494, 1373, 1339, 1283, 1165. $^1H$ NMR (600 MHz, DMSO-$d_6$): δ = 12.19 (s, 1H, OH-5), 10.41 (s, 1H, OH-4′), 7.80 (d, *J* = 8.8 Hz, 2H, H-2′,6′), 7.48 (d, *J* = 7.5 Hz, 2H, H-2″,6″), 7.42 (t, *J* = 7.5 Hz, 2H, H-3″,5″), 7.36 (t, *J* = 7.5 Hz, 1H, H-4″), 6.97 (d, *J* = 8.8 Hz, 2H, H-3′,5′), 6.90 (d, *J* = 2.0 Hz, 1H, H-8), 6.53 (d, *J* = 2.0 Hz, 1H, H-6), 5.26 (s, 2H, H-7″), 2.34 (s, 3H, $CH_3COO$-3). $^{13}C$ NMR (150 MHz, DMSO-$d_6$): δ = 175.07 (C-4), 167.91 ($CH_3\textit{C}OO$-3), 164.55 (C-7), 160.85 (C-4′), 160.77 (C-5), 156.49 (C-9), 156.36 (C-2), 136.00 (C-1″), 130.10 (C-2′,6′), 129.82 (C-3), 128.52 (C-3″,5″), 128.14 (C-4″), 127.84 (C-2″,6″), 119.27 (C-1′), 115.97 (C-3′,5′), 104.57 (C-10), 98.90 (C-6), 93.76 (C-8), 70.10 (C-7″), 20.21 ($\textit{C}H_3COO$-3). ESI-HRMS: 417.0993 $[M-H]^-$ (calcd. for $C_{24}H_{17}O_7$: 417.0980).

**3-*O*-Acetyl-7-*O*-benzyl-4′,5-di-*O*-methylkaempferol (14)**

To a suspension of **13** (171 mg, 0.41 mmol), $K_2CO_3$ (206 mg, 1.49 mmol) and acetone (8 mL) was added $Me_2SO_4$ (0.11 mL, 1.15 mmol). After the mixture was stirred at 30 °C for 12 h, another batch of $Me_2SO_4$ (0.02 mL, 0.2 mmol) was added. After another 12 h at 30 °C, the reaction mixture was diluted with water (20 mL) and extracted with EtOAc (30 mL). After removal of the solvent, the residue obtained was purified over silica gel column using acetone/petroleum ether (1:3) as solvents to

afford compound **14** (168 mg, 92%) as white powder, mp: 94-95 ºC. IR ($cm^{-1}$): 2955, 2919, 2851, 1776, 1607, 1467, 1349, 1298, 1258, 1170. $^{1}H$ NMR (400 MHz, DMSO-$d_6$): δ = 7.86 (d, *J* = 9.0 Hz, 2H), 7.51 (d, *J* = 7.1 Hz, 2H), 7.43 (t, *J* = 7.1 Hz, 2H), 7.37 (t, *J* = 7.1 Hz, 1H), 7.14 (d, *J* = 9.0 Hz, 2H), 6.97 (d, *J* = 2.1 Hz, 1H), 6.64 (d, *J* = 2.1 Hz, 1H), 5.26 (s, 2H), 3.85 (s, 3H), 3.84 (s, 3H), 2.30 (s, 3H). $^{13}C$ NMR (150 MHz, DMSO-$d_6$): δ = 169.08, 168.03, 163.22, 161.46, 160.48, 158.39, 152.35, 136.07, 132.65, 129.52, 128.57, 128.23, 128.03, 121.32, 114.51, 107.76, 96.94, 94.17, 70.14, 56.28, 55.50, 20.37. ESI-HRMS: 447.1455 $[M+H]^+$ (calcd. for $C_{26}H_{23}O_7$: 447.1438).

**3-*O*-Acetyl-4′,5-di-*O*-methylkaempferol (15)**

A mixture of **14** (150 mg, 0.34 mmol), 10% Pd/C (15 mg), MeOH (15 mL) and EtOAc (5 mL) was stirred at ambient temperature under a hydrogen balloon for 5 h. The mixture was filtered off through celite eluting with EtOAc (6 mL). The filtrate was concentrated to give the crude product, which was crystallized from acetone/petroleum ether (1:2) to afford **15** (110 mg, 92%) as off-white crystals, mp: 145-147 ºC. IR ($cm^{-1}$): 3263, 2926, 2855, 1730, 1603, 1509, 1459, 1298, 1257, 1175, 1102, 1030. $^{1}H$ NMR (400 MHz, DMSO-$d_6$): δ = 10.86 (s, 1H), 7.82 (d, *J* = 9.0 Hz, 2H), 7.12 (d, *J* = 9.0 Hz, 2H), 6.53 (d, *J* = 2.0 Hz, 1H), 6.41 (d, *J* = 2.0 Hz, 1H), 3.84 (s, 3H), 3.81 (s, 3H), 2.29 (s, 3H). $^{13}C$ NMR (150 MHz, DMSO-$d_6$): δ = 169.39, 168.42, 163.29, 161.58, 161.05, 158.58, 152.39, 132.53, 129.69, 121.59, 114.69, 106.68, 96.83, 95.36, 56.21, 55.67, 20.54. ESI-HRMS: 379.0770 $[M+Na]^+$ (calcd. for $C_{19}H_{16}O_7Na$: 379.0788).

**4′,5-Di-*O*-methylkaempferol (16)** [8]

Compound **15** (100 mg, 0.28 mmol) was stirred with 7.0 M methanolic ammonia (2 mL) at room temperature for 3 h. The precipitate formed was filtered off and crystallized

from acetone/petroleum ether (1:3) to give **16** (83 mg, 94%) as yellowish crystals, mp: 280-282 ºC. IR ($cm^{-1}$): 3179, 2934, 2838, 1602, 1563, 1501, 1378, 1297, 1256, 1207, 1183, 1031. $^{1}$H NMR (400 MHz, DMSO-$d_6$): δ = 10.51 (s, 1H, OH-7), 8.75 (s, 1H, OH-3), 8.09 (d, *J* = 8.9 Hz, 2H, H-2′,6′), 7.10 (d, *J* = 8.9 Hz, 2H, H-3′,5′), 6.50 (d, *J* = 1.8 Hz, 1H, H-8), 6.36 (d, *J* = 1.8 Hz, 1H, H-6), 3.83 (s, 6H, $OCH_3$-5,4′). $^{13}$C NMR (150 MHz, DMSO-$d_6$): δ = 171.00 (C-4), 162.55 (C-7), 160.57 (C-5), 159.96 (C-4′), 158.04 (C-9), 141.38 (C-2), 137.41 (C-3), 128.61 (C-2′,6′), 123.59 (C-1′), 114.03 (C-3′,5′), 105.20 (C-10), 95.92 (C-6), 94.76 (C-8), 55.98 ($OCH_3$-5 or 4′), 55.31 ($OCH_3$-5 or 4′). ESI-HRMS: 315.0852 $[M+H]^+$ (calcd. for $C_{17}H_{15}O_6$: 315.0863).

**7-*O*-Benzyl-4′-*O*-methylkaempferol (17)**

The mixture of compound **8** (1 g, 1.99 mmol), dimethyl sulfate (0.29 mL, 3.04 mmol), anhydrous $K_2CO_3$ (994 mg, 7.2 mmol) and acetone (27 mL) was refluxed for 2 h. Methanol (9 mL) was added to the mixture and the reaction continued for 24 h. Excess of solvent was removed and ice water (30 mL) was poured onto the obtained residue with stirring. The mixture was neutralized with 0.5 M HCl (aq), and extracted with $CHCl_3$ (40 mL). After evaporation of solvents, the yellow powder was crystallized from $CH_2Cl_2$/MeOH (1:4) to provide compound **17** (637 mg, 82%) as yellow needles, mp: 168-170 ºC. IR ($cm^{-1}$): 3293, 2927, 1652, 1617, 1589, 1501, 1351, 1310, 1260, 1225, 1162, 1091, 1036. $^{1}$H NMR (400 MHz, DMSO-$d_6$): δ = 12.44 (s, 1H), 9.64 (s, 1H), 8.17 (d, *J* = 9.1 Hz, 2H), 7.48 (d, *J* = 7.1 Hz, 2H), 7.42 (t, *J* = 7.1 Hz, 2H), 7.36 (t, *J* = 7.1 Hz, 1H), 7.13 (d, *J* = 9.1 Hz, 2H), 6.86 (d, *J* = 2.1 Hz, 1H), 6.45 (d, *J* = 2.1 Hz, 1H), 5.24 (s, 2H), 3.85 (s, 3H).$^{13}$C NMR (150 MHz, DMSO-$d_6$): δ = 176.13, 163.98, 160.62, 160.43, 156.08, 146.76, 136.39, 136.16, 129.39, 128.52, 128.11, 127.83, 123.15, 114.08, 104.22, 98.06, 92.98, 69.97, 55.39. ESI-HRMS: 389.1018 $[M-H]^-$ (calcd. for $C_{23}H_{17}O_6$: 389.1031).

**4′-*O*-Methylkaempferol (kaempferide) (18)** [9]

A mixture of **17** (410 mg, 1.05 mmol), 10% Pd/C (45 mg), MeOH (27 mL) and EtOAc (9 mL) was stirred at room temperature under a hydrogen balloon for 5 h. The mixture was filtered off through celite eluting with EtOAc (10 mL). The filtrate was concentrated to give the crude product, which was crystallized from acetone/petroleum ether (1:2) to yield **18** (293 mg, 93%) as yellow crystals, mp: 177-178 ºC. IR ($cm^{-1}$): 3445, 3291, 2925, 2854, 1615, 1510, 1374, 1310, 1253, 1172. $^{1}H$ NMR (400 MHz, DMSO-$d_6$): δ = 12.44 (s, 1H, OH-5), 10.83 (s, 1H, OH-7), 9.53 (s, 1H, OH-3), 8.14 (d, *J* = 9.1 Hz, 2H, H-2′,6′), 7.11 (d, *J* = 9.1 Hz, 2H, H-3′,5′), 6.46 (d, *J* = 2.0 Hz, 1H, H-8), 6.20 (d, *J* = 2.0 Hz, 1H, H-6), 3.84 (s, 3H, $OCH_3$-4′). $^{13}C$ NMR (150 MHz, DMSO-$d_6$): δ = 176.01 (C-4), 164.00 (C-7), 160.73 (C-5), 160.50 (C-4′), 156.25 (C-9), 146.28 (C-2), 136.06 (C-3), 129.34 (C-2′,6′), 123.27 (C-1′), 114.06 (C-3′,5′), 103.10 (C-10), 98.25 (C-6), 93.53 (C-8), 55.37 ($OCH_3$-4′). ESI-HRMS: 299.0560 $[M-H]^-$ (calcd. for $C_{16}H_{11}O_6$: 299.0561).

**7-*O*-Benzylkaempferol (19)**

The solution of **8** (400 mg, 0.8 mmol) in 7.0 M methanolic ammonia (9 mL) was stirred at room temperature for 3 h. The precipitate formed was filtered off and crystallized from acetone/MeOH (1:4) to give **19** (284 mg, 95%) as yellow crystals, mp: 183-184 ºC. IR ($cm^{-1}$): 3436, 2925, 1655, 1605, 1586, 1498, 1351, 1309, 1227, 1172. $^{1}H$ NMR (400 MHz, DMSO-$d_6$): δ = 12.48 (s, 1H), 10.14 (s, 1H), 9.53 (s, 1H), 8.08 (d, *J* = 8.9 Hz, 2H), 7.48 (d, *J* = 7.1 Hz, 2H), 7.42 (t, *J* = 7.1 Hz, 2H), 7.36 (t, *J* = 7.1 Hz, 1H), 6.94 (d, *J* = 8.9 Hz, 2H), 6.84 (d, *J* = 2.1 Hz, 1H), 6.44 (d, *J* = 2.1 Hz, 1H), 5.24 (s, 2H). $^{13}C$ NMR (100 MHz, DMSO-$d_6$): δ = 176.02, 163.89, 160.42, 159.34, 156.02, 147.31, 136.19, 136.01, 129.58, 128.54, 128.13, 127.86, 121.56, 115.47, 104.17, 98.03,

92.91, 69.95. ESI-HRMS: 375.0883 $[M-H]^-$ (calcd. for $C_{22}H_{15}O_6$: 375.0874).

**7-*O*-Benzyl-3,4′-di-*O*-methylkaempferol (20)** [10]

A suspension of **19** (120 mg, 0.32 mmol), anhydrous $K_2CO_3$ (138 mg, 1 mmol) and $Me_2SO_4$ (0.08 mL, 0.84 mmol) in dry acetone (5 mL) was stirred for 12 h at room temperature. The mixture was diluted with water (8 mL), neutralized with 0.5 M HCl (aq) and extracted with EtOAc. After evaporation of solvents, the yellow powder was purified over silica gel column using acetone/petroleum ether (1:5) as solvents to provide **20** (112 mg, 87%) as pale yellow granular crystals, mp: 113-114 ºC. IR ($cm^{-1}$): 3461, 2928, 1658, 1603, 1497, 1378, 1259, 1204, 1177. $^1H$ NMR (400 MHz, DMSO-$d_6$): δ = 12.63 (s, 1H), 8.05 (d, *J* = 9.0 Hz, 2H), 7.47 (d, *J* = 7.1 Hz, 2H), 7.42 (t, *J* = 7.1 Hz, 2H), 7.36 (t, *J* = 7.1 Hz, 1H), 7.15 (d, *J* = 9.0 Hz, 2H), 6.87 (d, *J* = 2.1 Hz, 1H), 6.47 (d, *J* = 2.1 Hz, 1H), 5.24 (s, 2H), 3.87 (s, 3H), 3.81 (s, 3H). $^{13}C$ NMR (150 MHz, DMSO-$d_6$): δ = 178.10, 164.16, 161.46, 160.97, 156.29, 155.60, 138.19, 136.09, 130.04, 128.51, 128.10, 127.81, 122.07, 114.28, 105.39, 98.43, 93.30, 69.99, 59.77, 55.46. ESI-HRMS: 427.1153 $[M+Na]^+$ (calcd. for $C_{24}H_{20}O_6Na$: 427.1152).

**7-*O*-Benzyl-3,4′,5-tri-*O*-methylkaempferol (21)** [11]

The mixture of **19** (120 mg, 0.32 mmol), $Me_2SO_4$ (0.11 mL, 1.15 mmol) and $K_2CO_3$ (177 mg, 1.28 mmol) in acetone (6 mL) was stirred for 12 h at 30 °C. Another batch of $Me_2SO_4$ (0.03 mL, 0.32 mmol) was then added. After another 12 h at 30 °C, the mixture was diluted with water (10 mL) and extracted with EtOAc (20 mL). After evaporation of solvents, the residue was purified over silica gel column using acetone/petroleum ether (1:3) as solvents to provide **21** (127 mg, 95%) as white powder, mp: 133-135 ºC. IR ($cm^{-1}$): 2925, 2853, 1628, 1604, 1455, 1348, 1295, 1256, 1211, 1176, 1013. $^1H$ NMR (400 MHz, $CDCl_3$): δ = 8.06 (d, *J* = 9.1 Hz, 2H), 7.48–7.37

(m, 5H), 7.01 (d, $J$ = 9.1 Hz, 2H), 6.59 (d, $J$ = 2.0 Hz, 1H), 6.43 (d, $J$ = 2.0 Hz, 1H), 5.14 (s, 2H), 3.96 (s, 3H), 3.89 (s, 3H), 3.88 (s, 3H). $^{13}$C NMR (150 MHz, $CDCl_3$): δ = 174.20, 163.06, 161.32, 161.24, 158.91, 152.88, 141.27, 135.90, 129.98, 128.95, 128.62, 127.77, 123.42, 114.07, 109.85, 96.45, 93.55, 70.67, 60.05, 56.57, 55.54. ESI-HRMS: 419.1482 $[M+H]^+$ (calcd. for $C_{25}H_{23}O_6$: 419.1489).

**3,4′-Di-*O*-methylkaempferol (22)** [7]

A mixture of **20** (100 mg, 0.25 mmol), 10% Pd/C (10 mg), MeOH (9 mL) and EtOAc (3 mL) was stirred at room temperature under a hydrogen balloon for 5 h. The mixture was filtered off through celite eluting with EtOAc (4 mL). After concentration of the filtrate, the yellow powder was crystallized from acetone/petroleum ether (1:4) to afford **22** (71 mg, 91%) as pale yellow needles, mp: 216-218 ºC. IR (cm$^{-1}$): 3428, 3111, 2933, 1651, 1607, 1574, 1497, 1365, 1305, 1263, 1223, 1164, 1019. $^{1}$H NMR (400 MHz, DMSO-$d_6$): δ = 12.64 (s, 1H, OH-5), 10.88 (s, 1H, OH-7), 8.02 (d, $J$ = 9.0 Hz, 2H, H-2′,6′), 7.13 (d, $J$ = 9.0 Hz, 2H, H-3′,5′), 6.46 (d, $J$ = 2.0 Hz, 1H, H-8), 6.21 (d, $J$ = 2.0 Hz, 1H, H-6), 3.86 (s, 3H, $OCH_3$-4′), 3.79 (s, 3H, $OCH_3$-3). $^{13}$C NMR (100 MHz, DMSO-$d_6$): δ = 177.96 (C-4), 164.22 (C-7), 161.36 (C-4′), 161.27 (C-5), 156.44 (C-9), 155.24 (C-2), 137.94 (C-3), 130.02 (C-2′,6′), 122.20 (C-1′), 114.27 (C-3′,5′), 104.29 (C-10), 98.63 (C-6), 93.79 (C-8), 59.79 ($OCH_3$-3), 55.46 ($OCH_3$-4′). ESI-HRMS: 315.0858 $[M+H]^+$ (calcd. for $C_{17}H_{15}O_6$: 315.0863).

**3,4′,5-Tri-*O*-methylkaempferol (23)** [5]

A mixture of **21** (100 mg, 0.24 mmol), 10% Pd/C (10 mg), MeOH (12 mL) and EtOAc (4 mL) was stirred at room temperature under a hydrogen balloon for 5 h. The mixture was filtered off through celite eluting with EtOAc (6 mL). After concentration of the filtrate, the pale green powder was crystallized from acetone/MeOH (1:3) to afford **23**

(70 mg, 89%) as off-white crystals, mp: 151-152 ºC. IR ($cm^{-1}$): 3134, 2923, 2852, 1605, 1575, 1470, 1356, 1297, 1256, 1189. $^{1}$H NMR (400 MHz, DMSO-$d_6$): δ = 10.73 (s, 1H, OH-7), 7.97 (d, $J$ = 9.0 Hz, 2H, H-2′,6′), 7.11 (d, $J$ = 9.0 Hz, 2H, H-3′,5′), 6.48 (d, $J$ = 2.0 Hz, 1H, H-8), 6.36 (d, $J$ = 2.0 Hz, 1H, H-6), 3.84 (s, 3H, $OCH_3$-5), 3.81 (s, 3H, $OCH_3$-4′), 3.71 (s, 3H, $OCH_3$-3). $^{13}$C NMR (100 MHz, DMSO-$d_6$): δ = 172.09 (C-4), 162.50 (C-7), 160.80 (C-5), 160.74 (C-4′), 158.11 (C-9), 151.51 (C-2), 139.99 (C-3), 129.52 (C-2′,6′), 122.63 (C-1′), 114.15 (C-3′,5′), 107.42 (C-10), 96.15 (C-6), 94.88 (C-8), 59.28 ($OCH_3$-3), 55.90 ($OCH_3$-4′), 55.39 ($OCH_3$-5). ESI-HRMS: 329.1016 $[M+H]^+$ (calcd. for $C_{18}H_{17}O_6$: 329.1020).

# References


1. Prawat, H.; Mahidol, C.; Ruchirawat, S.; Prawat, U.; Tuntiwachwuttikul, P.; Tooptakong, U.; Taylor, W. C.; Pakawatchal, C.; Skelton, B. W.; White, A. H. *Phytochemistry* **1995**, *40*, 1167–1173.
2. Kawamura, T.; Hayashi, M.; Mukai, R.; Terao, J.; Nemoto, H. *Synthesis* **2014**, *46*, 170–174.
3. Kimura, Y.; Takido, M.; Takahashi, S.; Kimishima, M. *Yakugaku Zasshi* **1967**, *87*, 440–443.
4. Zhang, X.-F.; Hung, T. M.; Phuong, P. T.; Ngoc, T. M.; Min, B.-S.; Song, K.-S.; Seong, Y. H.; Bae, K.-H. *Arch. Pharm. Res.* **2006**, *29*, 1102–1108.
5. Rossi, M. H.; Yoshida, M.; Maia, J. G. S. *Phytochemistry* **1997**, *45*, 1263–1269.
6. Wang, Y.; Hamburger, M.; Gueho, J.; Hostettmann, K. *Phytochemistry* **1989**, *28*, 2323–2327.
7. Nakatani, N.; Jitoe, A.; Masuda, T.; Yonemori, S. *Agric. Biol. Chem.* **1991**, *55*,

455–460.

8. Kanao, M.; Shimokoriyama, M. *Acta Phytochimica* **1949**, *15*, 229–231.

9. Wadher, S. J.; Tapas, A. R.; Yeole, P. G. *Int. J. Chem. Sci*. **2006**, *4*, 761–766.

10. Bhrara, S. C.; Jain, A. C.; Seshadri, T. R. *Indian J. Chem.* **1965**, *3*, 68–70.

11. Rajagopalan, S.; Rao, P. R.; Rao, K. V.; Seshadri, T. R. *Proc. Indian AS*, *Sect. A* **1949**, *29*A, 9–15.

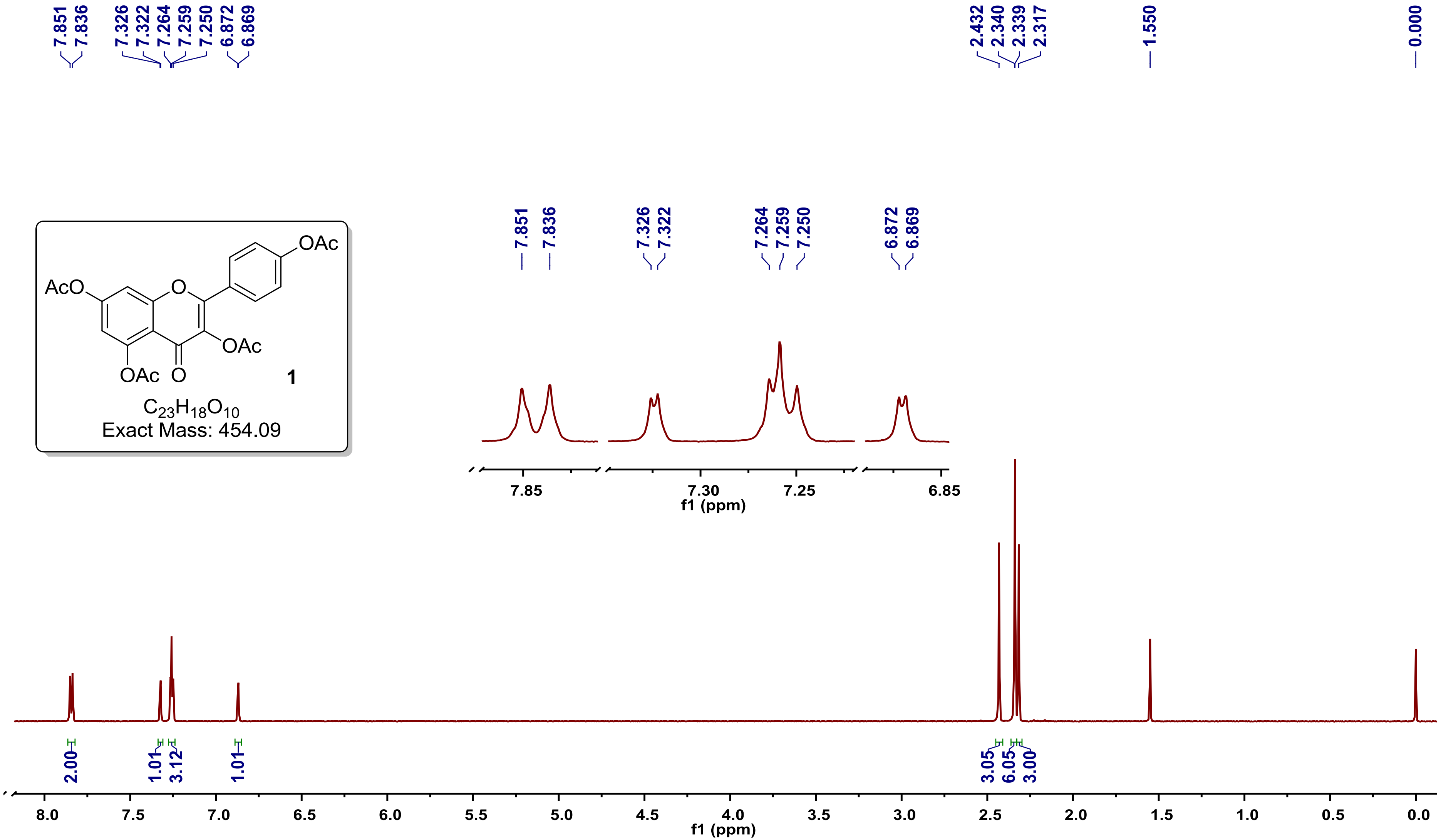
7.851
7.836
7.326
7.322
7.264
7.259
7.250
6.872
6.869
2.432
2.340
2.339
2.317
1.550
0.000
AcO
O
OAc
OAc
OAc
O
1
C23H18O10
Exact Mass: 454.09
7.85
7.30
7.25
6.85
f1 (ppm)
2.00
1.01
3.12
1.01
3.05
6.05
3.00
8.0
7.5
7.0
6.5
6.0
5.5
5.0
4.5
4.0
3.5
3.0
2.5
2.0
1.5
1.0
0.5
0.0
f1 (ppm)

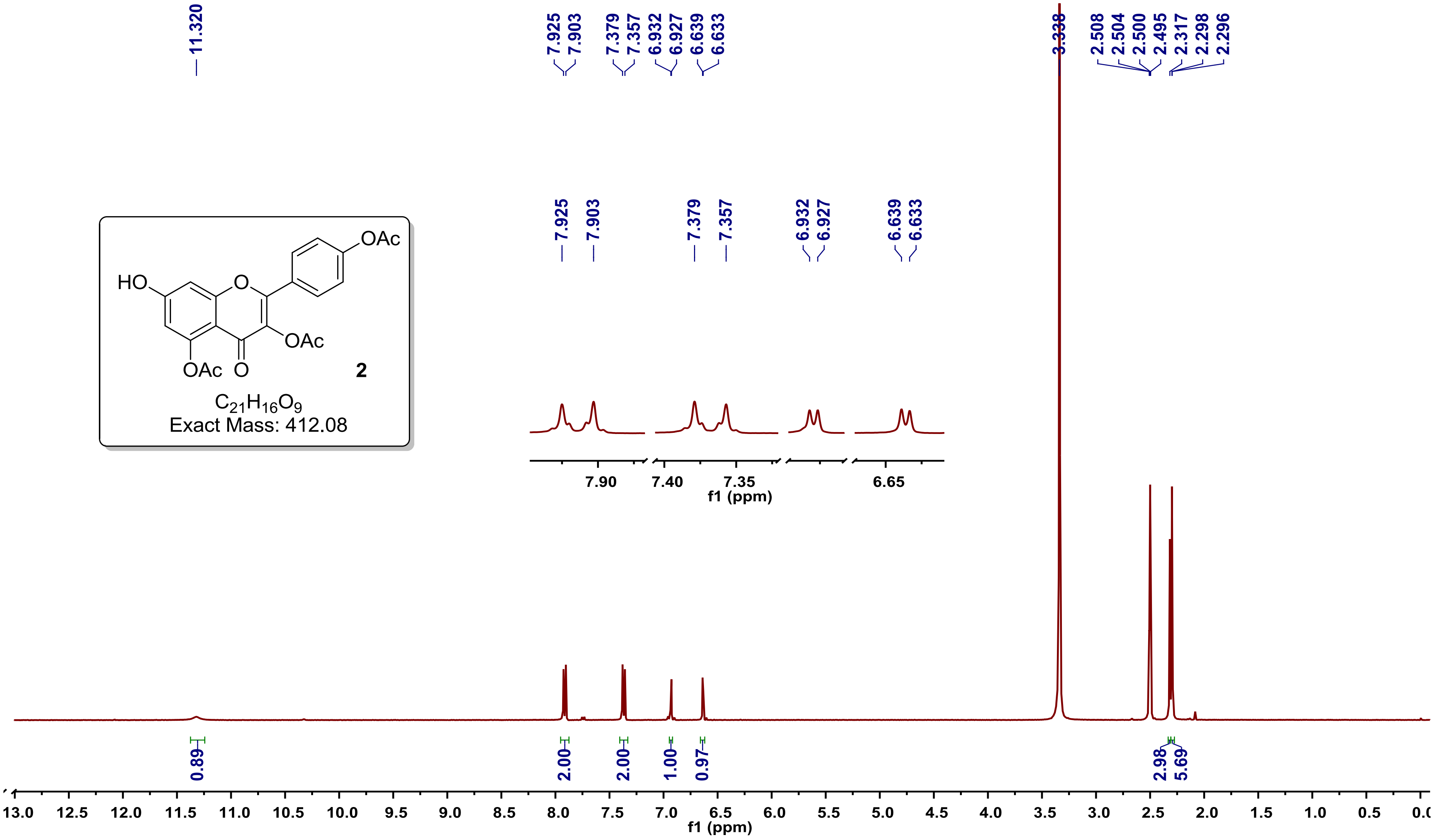
11.320
7.925
7.903
7.379
7.357
6.932
6.927
6.639
6.633
3.338
2.508
2.504
2.500
2.495
2.317
2.298
2.296
HO
O
OAc
OAc
OAc O
2
$C_{21}H_{16}O_9$
Exact Mass: 412.08
7.90 7.40 7.35 6.65
f1 (ppm)
0.89
2.00
2.00
1.00
0.97
2.98
5.69
13.0 12.5 12.0 11.5 11.0 10.5 10.0 9.5 9.0 8.5 8.0 7.5 7.0 6.5 6.0 5.5 5.0 4.5 4.0 3.5 3.0 2.5 2.0 1.5 1.0 0.5
f1 (ppm)

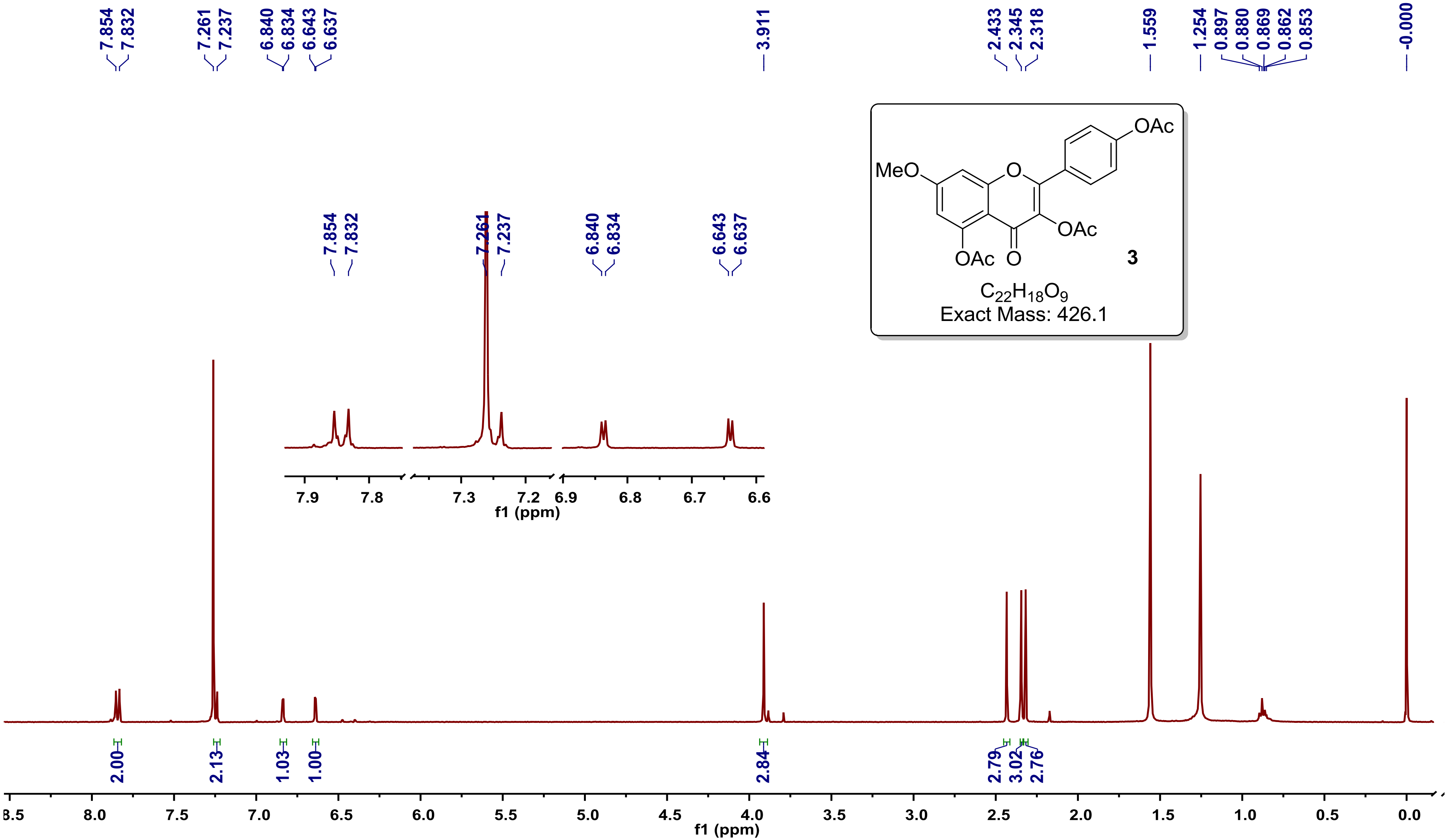
7.854
7.832
7.261
7.237
6.840
6.834
6.643
6.637
3.911
2.433
2.345
2.318
1.559
1.254
0.897
0.880
0.869
0.862
0.853
-0.000
MeO
O
OAc
OAc
OAc
O
3
$C_{22}H_{18}O_9$
Exact Mass: 426.1
7.9
7.8
7.3
7.2
6.9
6.8
6.7
6.6
f1 (ppm)
2.00
2.13
1.03
1.00
2.84
2.79
3.02
2.76
8.5
8.0
7.5
7.0
6.5
6.0
5.5
5.0
4.5
4.0
3.5
3.0
2.5
2.0
1.5
1.0
0.5
0.0
f1 (ppm)

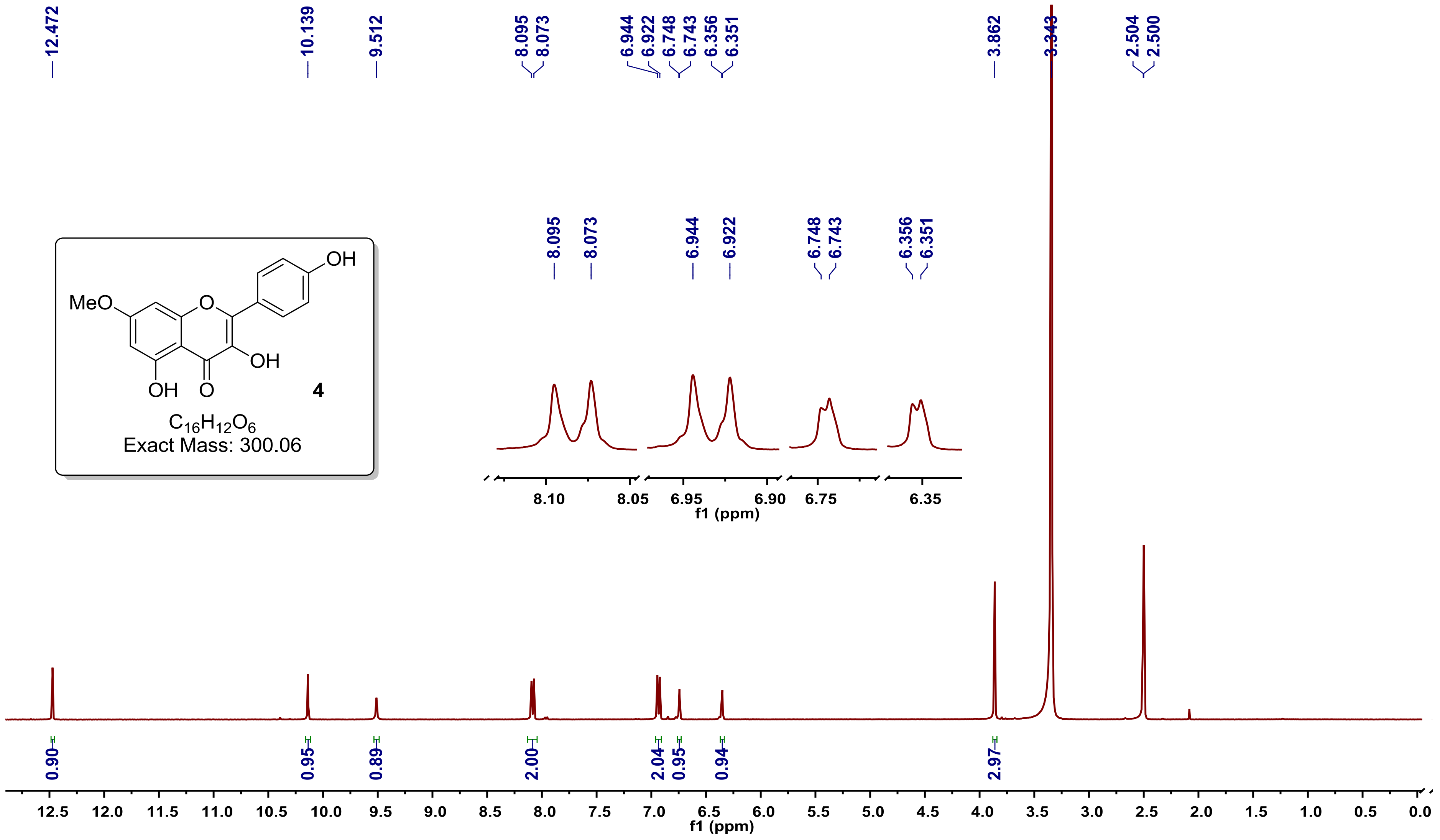
12.472
10.139
9.512
8.095
8.073
6.944
6.922
6.748
6.743
6.356
6.351
3.862
3.343
2.504
2.500
MeO
O
OH
OH
OH
O
4
$C_{16}H_{12}O_6$
Exact Mass: 300.06
8.10
8.05
6.95
6.90
6.75
6.35
f1 (ppm)
0.90
0.95
0.89
2.00
2.04
0.95
0.94
2.97
12.5
12.0
11.5
11.0
10.5
10.0
9.5
9.0
8.5
8.0
7.5
7.0
6.5
6.0
5.5
5.0
4.5
4.0
3.5
3.0
2.5
2.0
1.5
1.0
0.5
0.0
f1 (ppm)

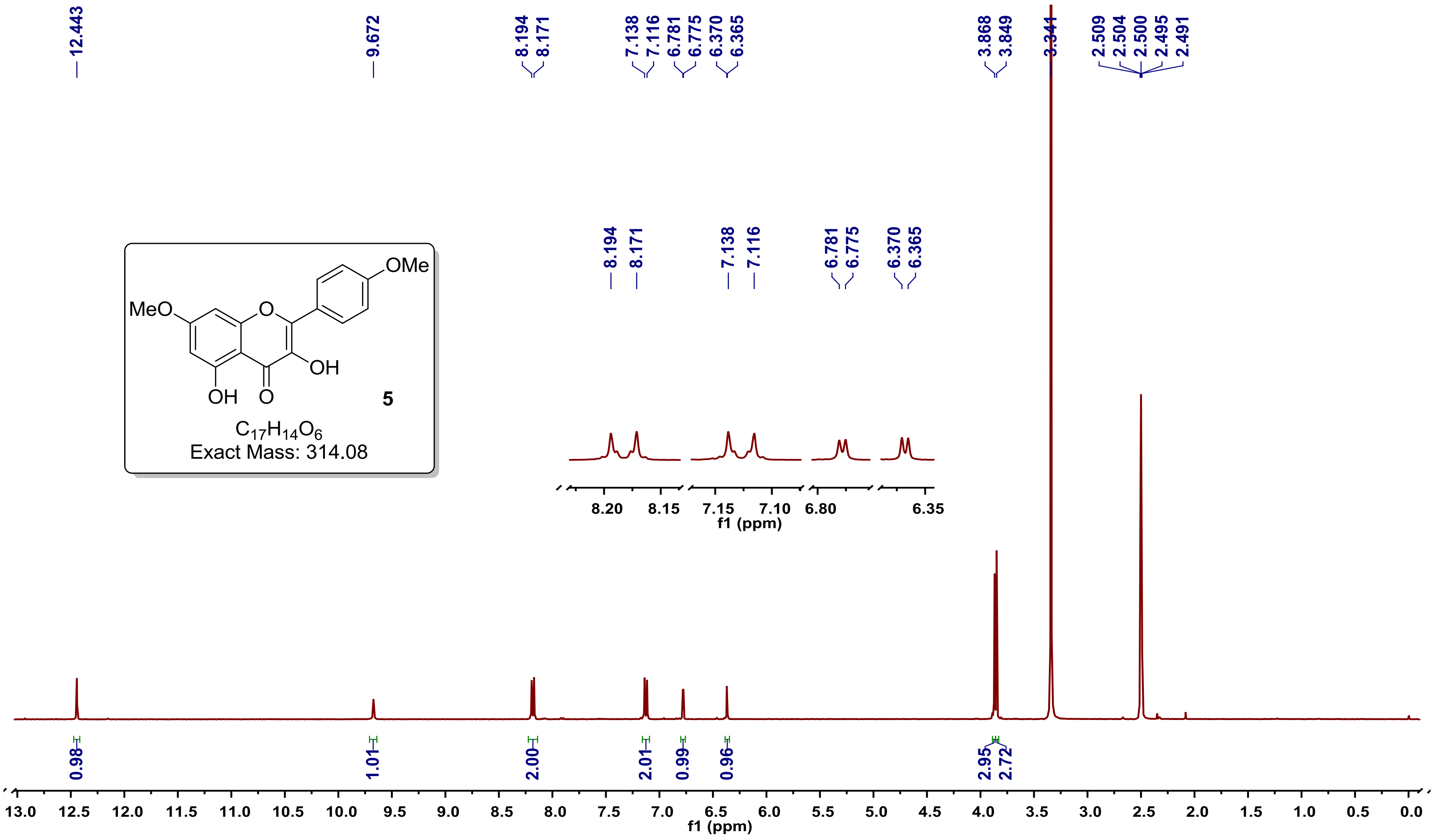

MeO
OMe
OH
OH
O
O
5
$C_{17}H_{14}O_6$
Exact Mass: 314.08
12.443
9.672
8.194
8.171
7.138
7.116
6.781
6.775
6.370
6.365
3.868
3.849
3.341
2.509
2.504
2.500
2.495
2.491
0.98
1.01
2.00
2.01
0.99
0.96
2.95
2.72
f1 (ppm)

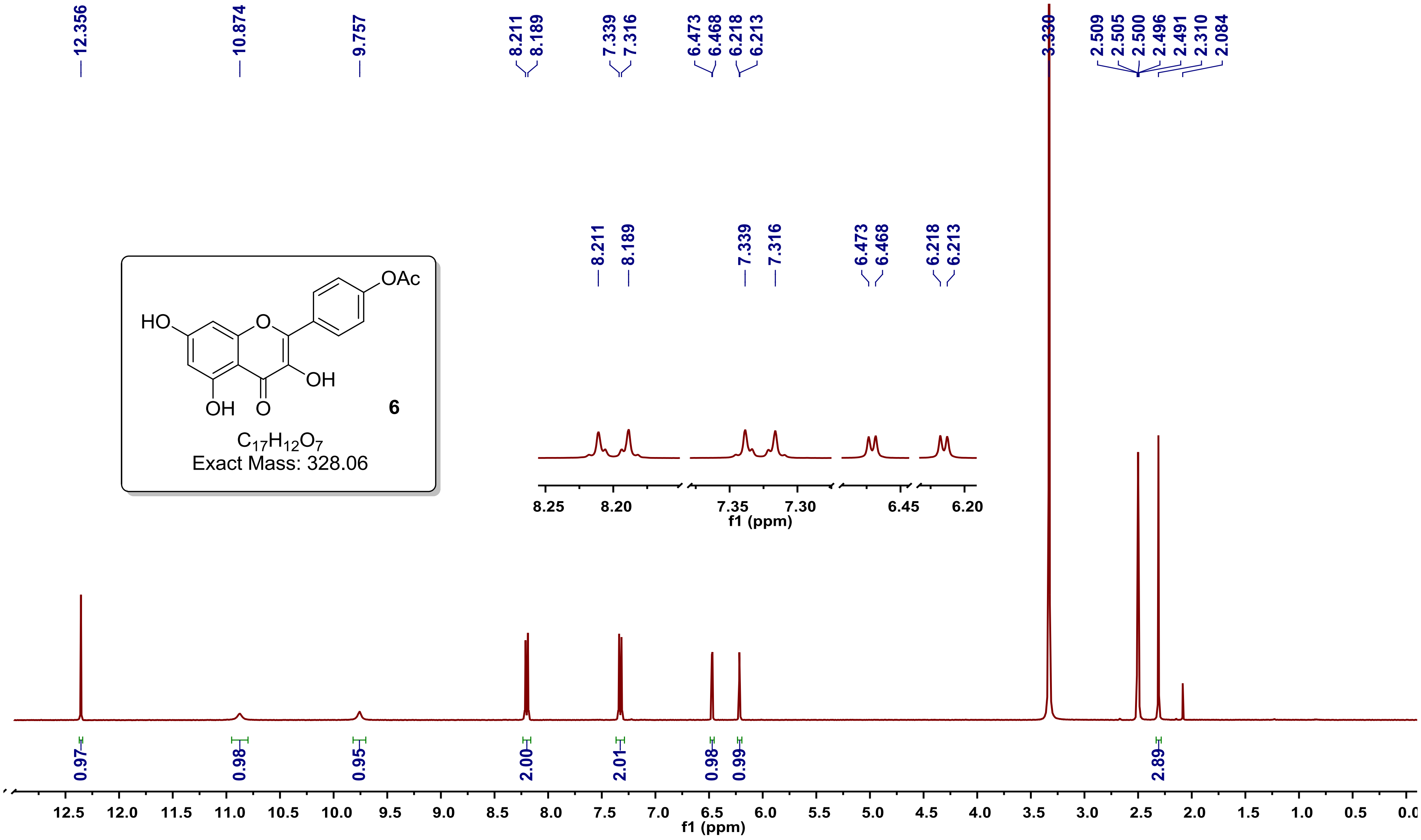
12.356
10.874
9.757
8.211
8.189
7.339
7.316
6.473
6.468
6.218
6.213
3.330
2.509
2.505
2.500
2.496
2.491
2.310
2.084
HO
O
OAc
OH
OH
O
6
$C_{17}H_{12}O_7$
Exact Mass: 328.06
8.25
8.20
7.35
7.30
6.45
6.20
f1 (ppm)
0.97
0.98
0.95
2.00
2.01
0.98
0.99
2.89
12.5
12.0
11.5
11.0
10.5
10.0
9.5
9.0
8.5
8.0
7.5
7.0
6.5
6.0
5.5
5.0
4.5
4.0
3.5
3.0
2.5
2.0
1.5
1.0
0.5
0.0
f1 (ppm)

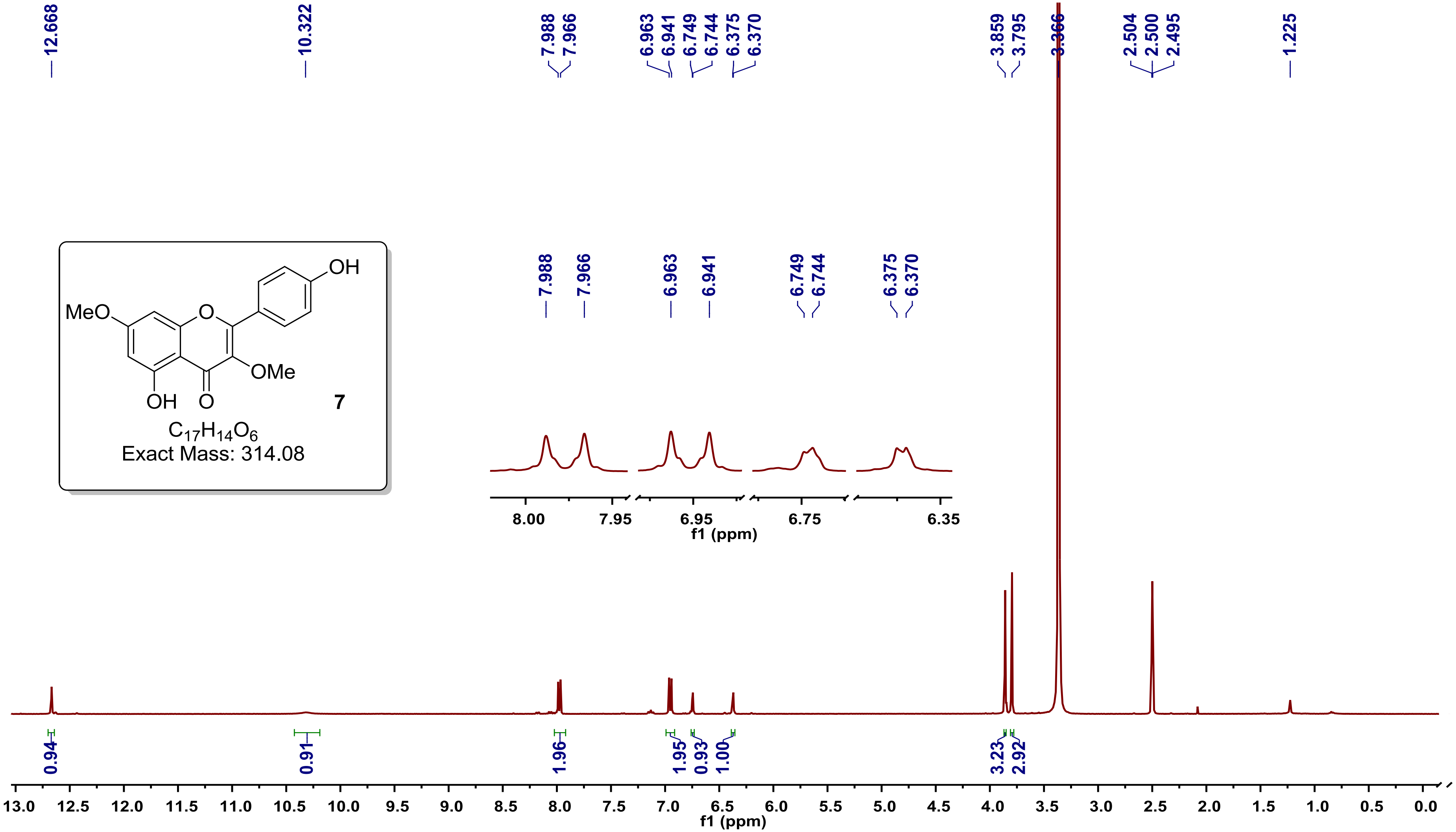

12.668
10.322
7.988
7.966
6.963
6.941
6.749
6.744
6.375
6.370
3.859
3.795
3.366
2.504
2.500
2.495
1.225
MeO
O
OH
OMe
OH
O
7
$C_{17}H_{14}O_6$
Exact Mass: 314.08
8.00
7.95
6.95
6.75
6.35
f1 (ppm)
0.94
0.91
1.96
1.95
0.93
1.00
3.23
2.92
13.0 12.5 12.0 11.5 11.0 10.5 10.0 9.5 9.0 8.5 8.0 7.5 7.0 6.5 6.0 5.5 5.0 4.5 4.0 3.5 3.0 2.5 2.0 1.5 1.0 0.5 0.0
f1 (ppm)

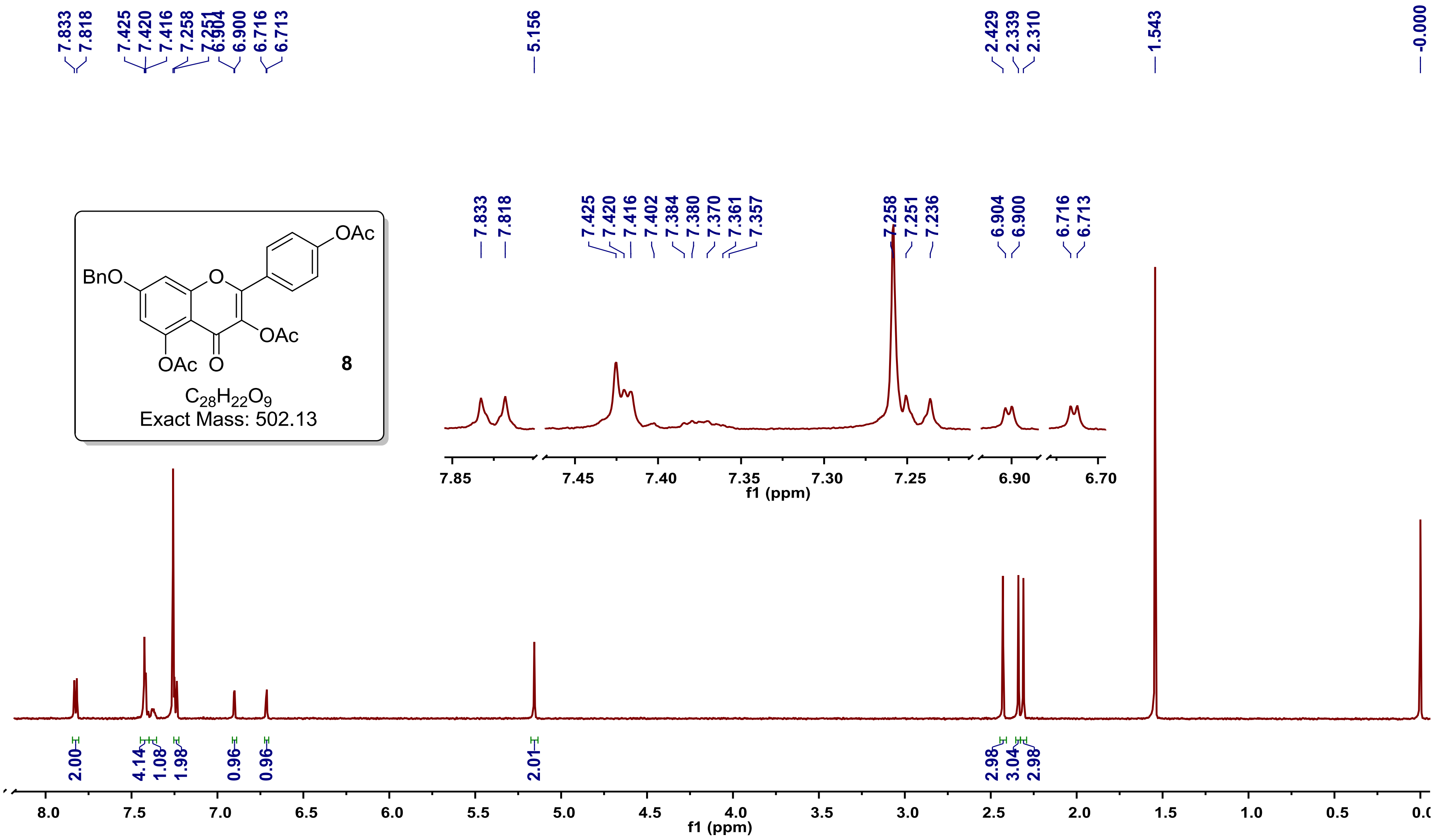

7.833
7.818
7.425
7.420
7.416
7.258
7.251
6.904
6.900
6.716
6.713
5.156
2.429
2.339
2.310
1.543
-0.000
BnO
OAc
OAc
OAc
O
O
8
$C_{28}H_{22}O_9$
Exact Mass: 502.13
7.402
7.384
7.380
7.370
7.361
7.357
7.236
f1 (ppm)
2.00
4.14
1.08
1.98
0.96
0.96
2.01
2.98
3.04
2.98

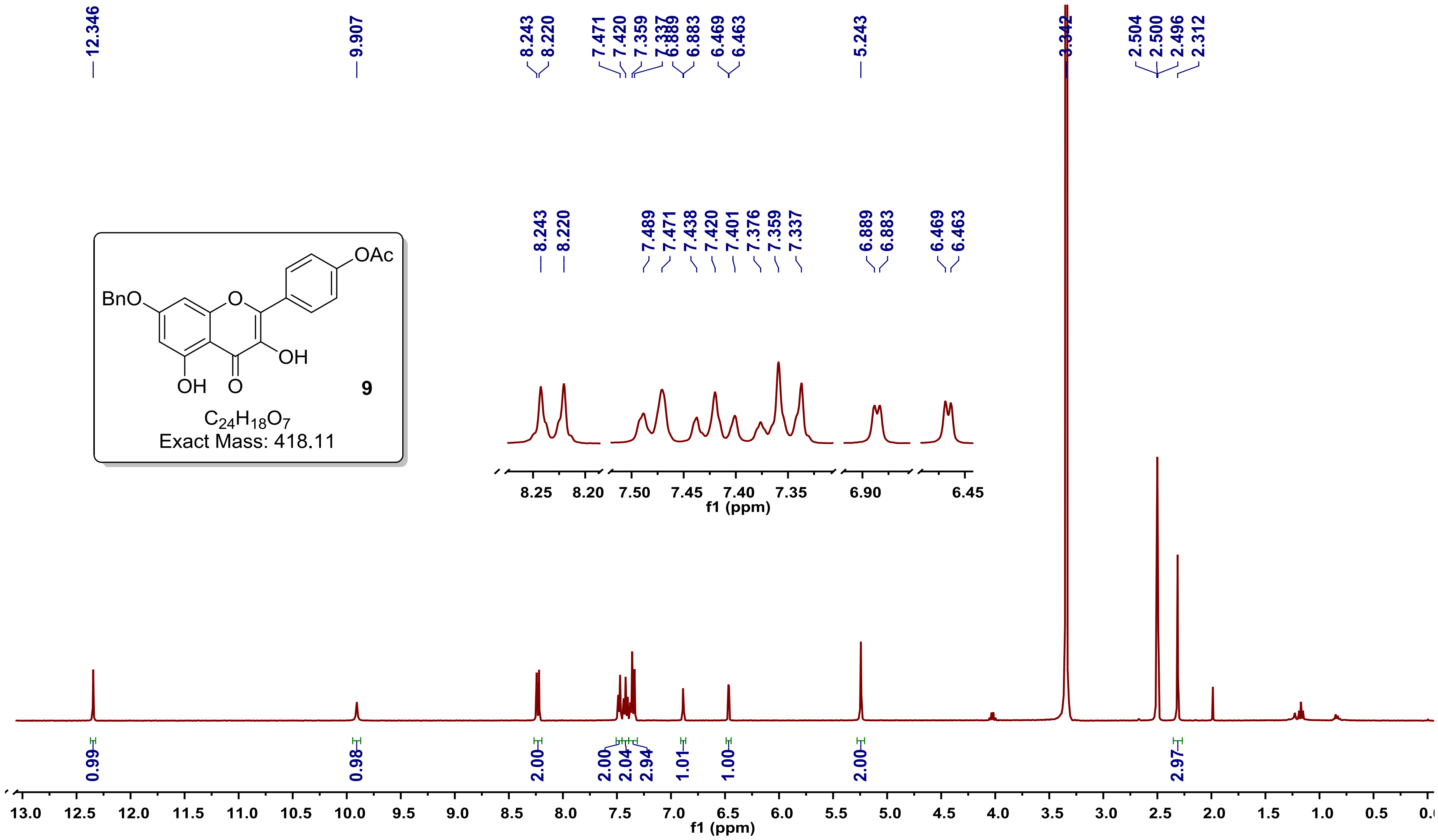

BnO
OAc
OH
OH O
9
$C_{24}H_{18}O_7$
Exact Mass: 418.11
12.346
9.907
8.243
8.220
7.471
7.420
7.359
7.337
6.889
6.883
6.469
6.463
5.243
3.342
2.504
2.500
2.496
2.312
7.489
7.471
7.438
7.420
7.401
7.376
7.359
7.337
0.99
0.98
2.00
2.00
2.04
2.94
1.01
1.00
2.00
2.97
f1 (ppm)

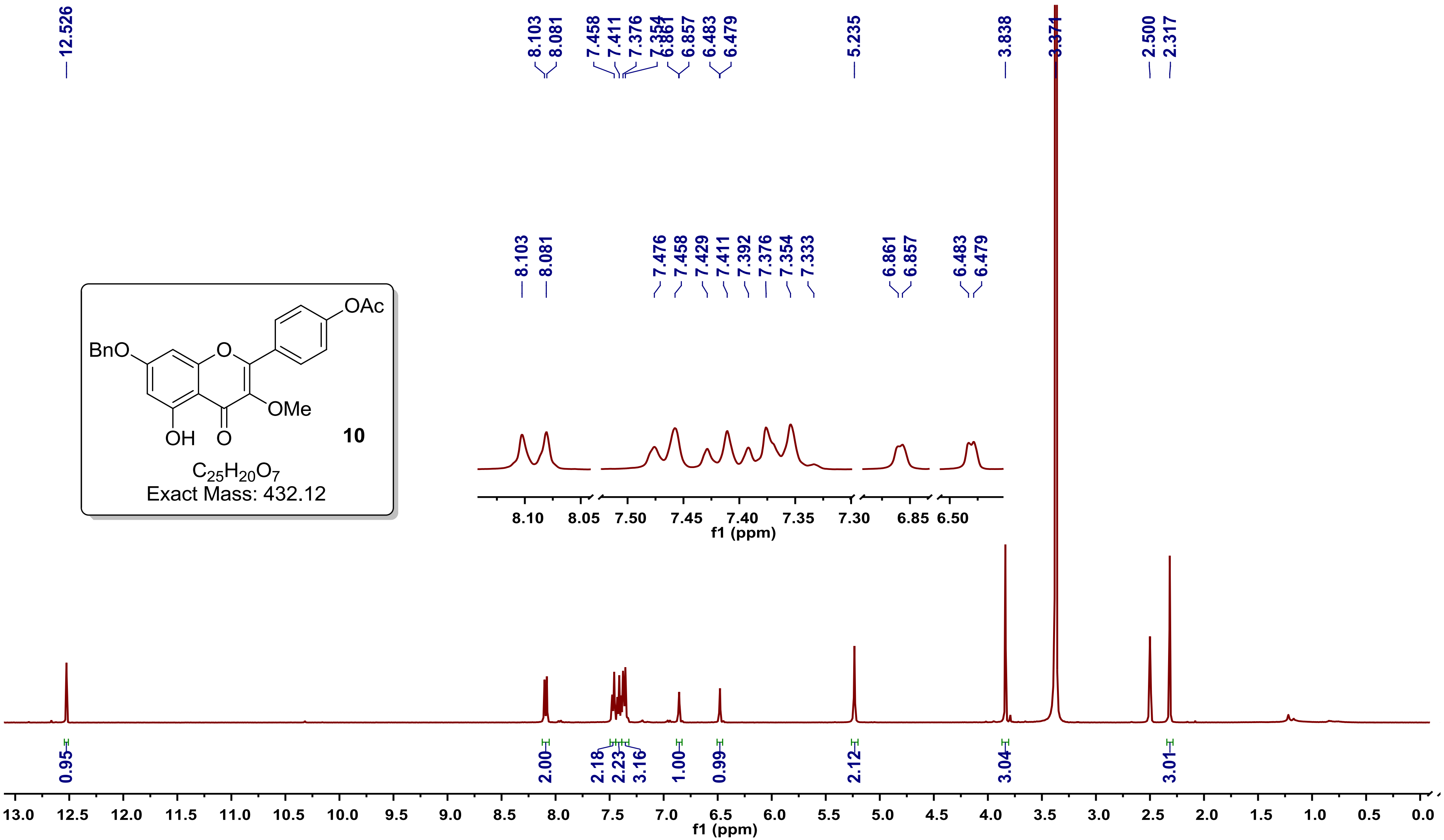
12.526
8.103
8.081
7.458
7.411
7.376
7.354
6.861
6.857
6.483
6.479
5.235
3.838
3.374
2.500
2.317
7.476
7.429
7.392
7.333
BnO
OAc
OMe
OH
O
10
$C_{25}H_{20}O_7$
Exact Mass: 432.12
8.10 8.05 7.50 7.45 7.40 7.35 7.30 6.85 6.50
f1 (ppm)
0.95
2.00
2.18
2.23
3.16
1.00
0.99
2.12
3.04
3.01
13.0 12.5 12.0 11.5 11.0 10.5 10.0 9.5 9.0 8.5 8.0 7.5 7.0 6.5 6.0 5.5 5.0 4.5 4.0 3.5 3.0 2.5 2.0 1.5 1.0 0.5 0.0
f1 (ppm)

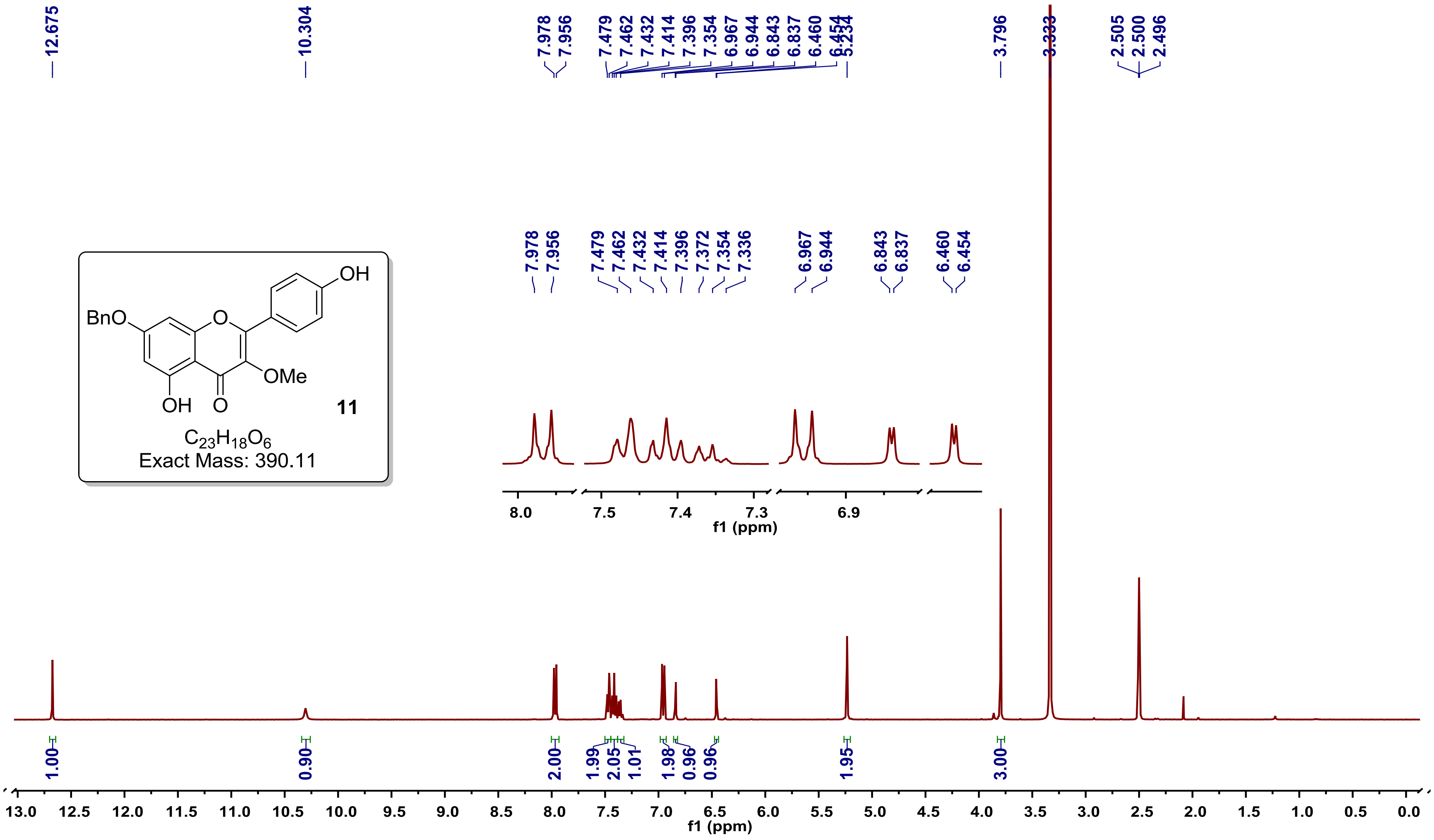
BnO
O
OH
OMe
OH
O
11
$C_{23}H_{18}O_6$
Exact Mass: 390.11
12.675
10.304
7.978
7.956
7.479
7.462
7.432
7.414
7.396
7.372
7.354
7.336
6.967
6.944
6.843
6.837
6.460
6.454
5.234
3.796
3.333
2.505
2.500
2.496
1.00
0.90
2.00
1.99
2.05
1.01
1.98
0.96
0.96
1.95
3.00
f1 (ppm)

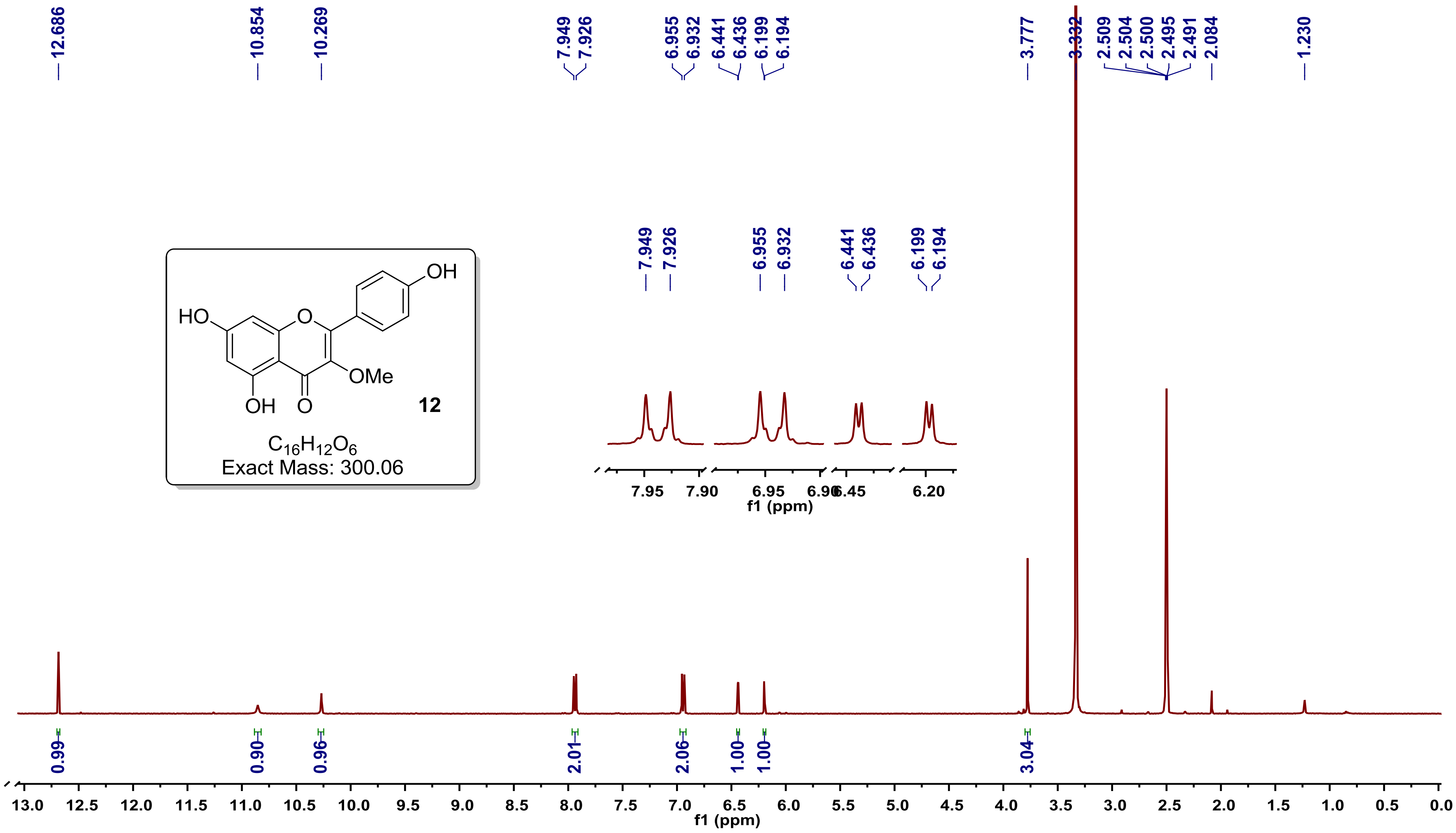
12.686
10.854
10.269
7.949
7.926
6.955
6.932
6.441
6.436
6.199
6.194
3.777
3.332
2.509
2.504
2.500
2.495
2.491
2.084
1.230
HO
O
OH
OMe
OH
O
12
$C_{16}H_{12}O_6$
Exact Mass: 300.06
7.95
7.90
6.95
6.90
6.45
6.20
f1 (ppm)
0.99
0.90
0.96
2.01
2.06
1.00
1.00
3.04
13.0
12.5
12.0
11.5
11.0
10.5
10.0
9.5
9.0
8.5
8.0
7.5
7.0
6.5
6.0
5.5
5.0
4.5
4.0
3.5
3.0
2.5
2.0
1.5
1.0
0.5
0.0
f1 (ppm)

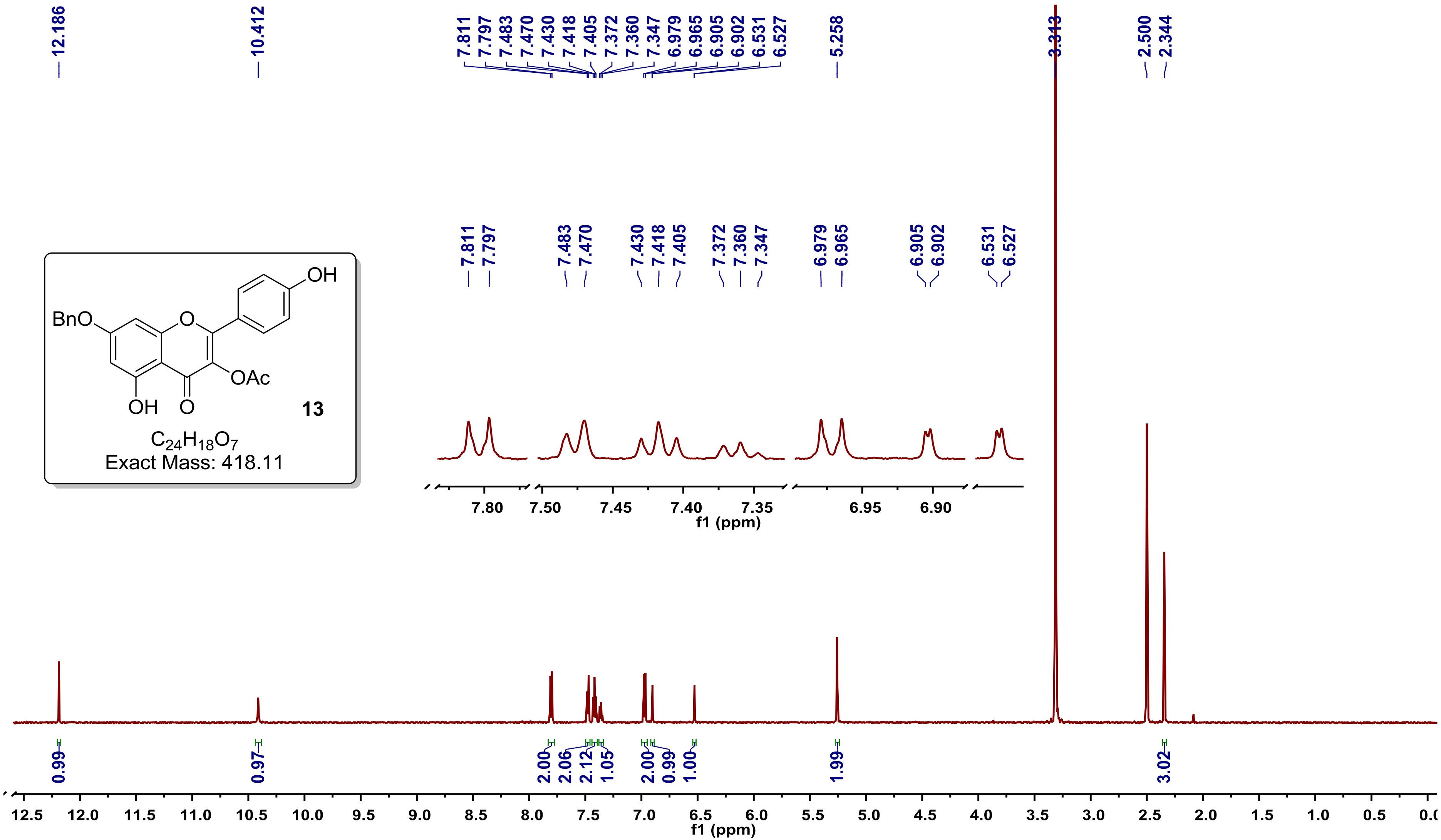
12.186
10.412
7.811 7.797 7.483 7.470 7.430 7.418 7.405 7.372 7.360 7.347 6.979 6.965 6.905 6.902 6.531 6.527
5.258
3.343
2.500 2.344
BnO
OH
OH O
OAc
13
C24H18O7
Exact Mass: 418.11
f1 (ppm)
0.99 0.97 2.00 2.06 2.12 1.05 2.00 0.99 1.00 1.99 3.02

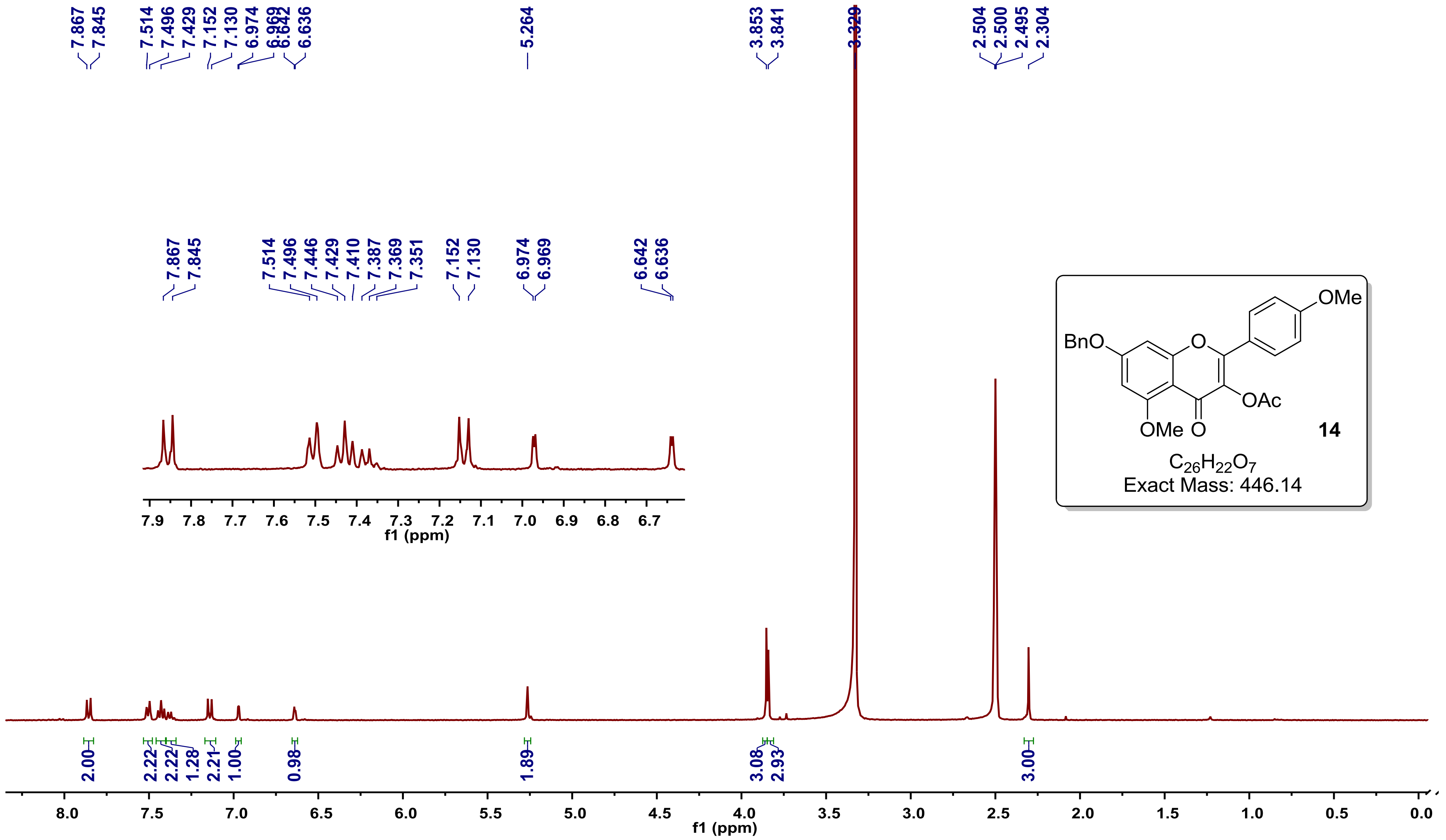
7.867
7.845
7.514
7.496
7.429
7.152
7.130
6.974
6.969
6.642
6.636
5.264
3.853
3.841
3.329
2.504
2.500
2.495
2.304
7.867
7.845
7.514
7.496
7.446
7.429
7.410
7.387
7.369
7.351
7.152
7.130
6.974
6.969
6.642
6.636
7.9 7.8 7.7 7.6 7.5 7.4 7.3 7.2 7.1 7.0 6.9 6.8 6.7
f1 (ppm)
OMe
BnO
O
OAc
OMe O
14
C26H22O7
Exact Mass: 446.14
2.00
2.22
2.22
1.28
2.21
1.00
0.98
1.89
3.08
2.93
3.00
8.0 7.5 7.0 6.5 6.0 5.5 5.0 4.5 4.0 3.5 3.0 2.5 2.0 1.5 1.0 0.5 0.0
f1 (ppm)

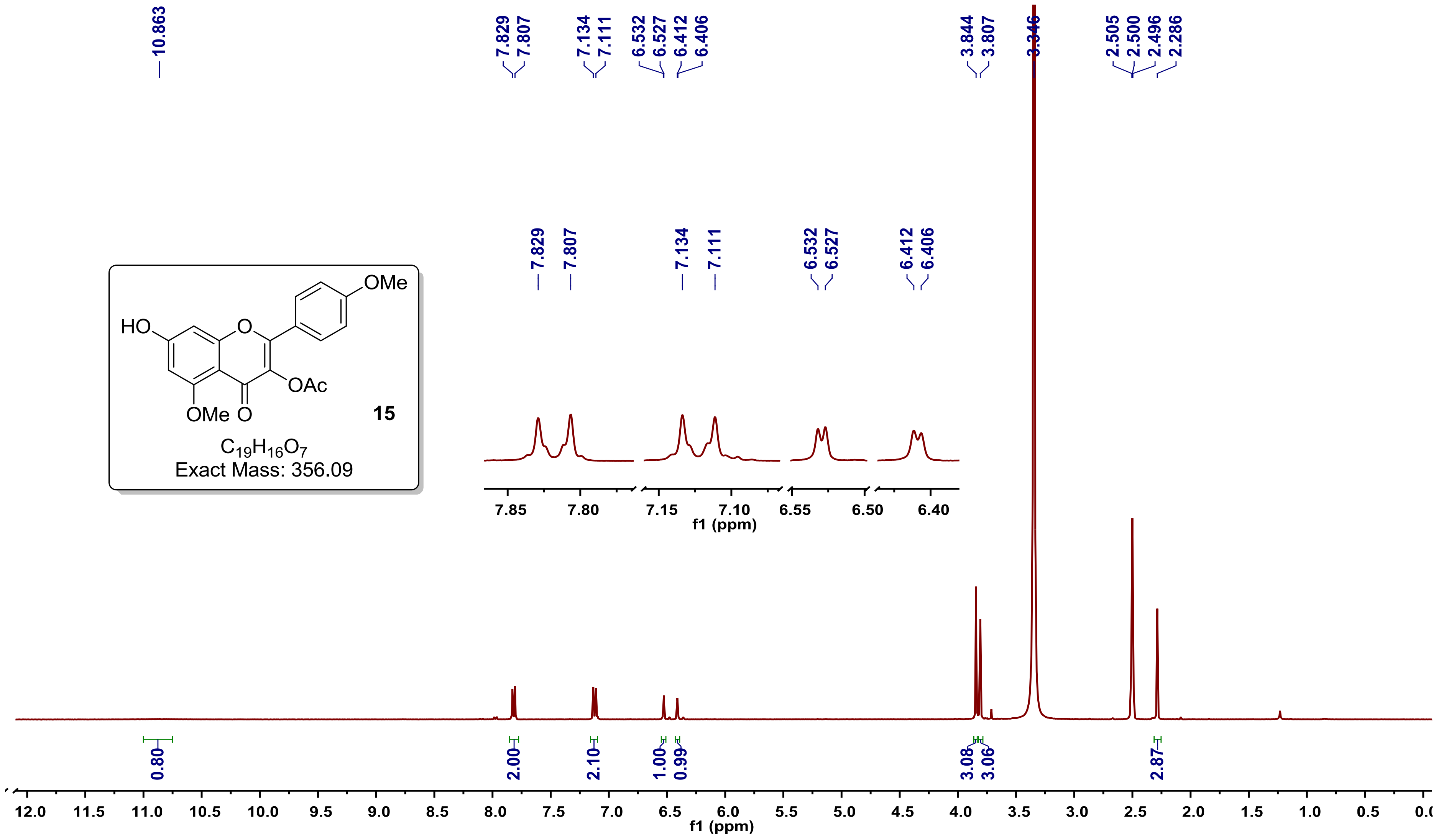
10.863
7.829
7.807
7.134
7.111
6.532
6.527
6.412
6.406
3.844
3.807
3.346
2.505
2.500
2.496
2.286
HO
O
OMe
OAc
OMe O
15
$C_{19}H_{16}O_7$
Exact Mass: 356.09
0.80
2.00
2.10
1.00
0.99
3.08
3.06
2.87
f1 (ppm)

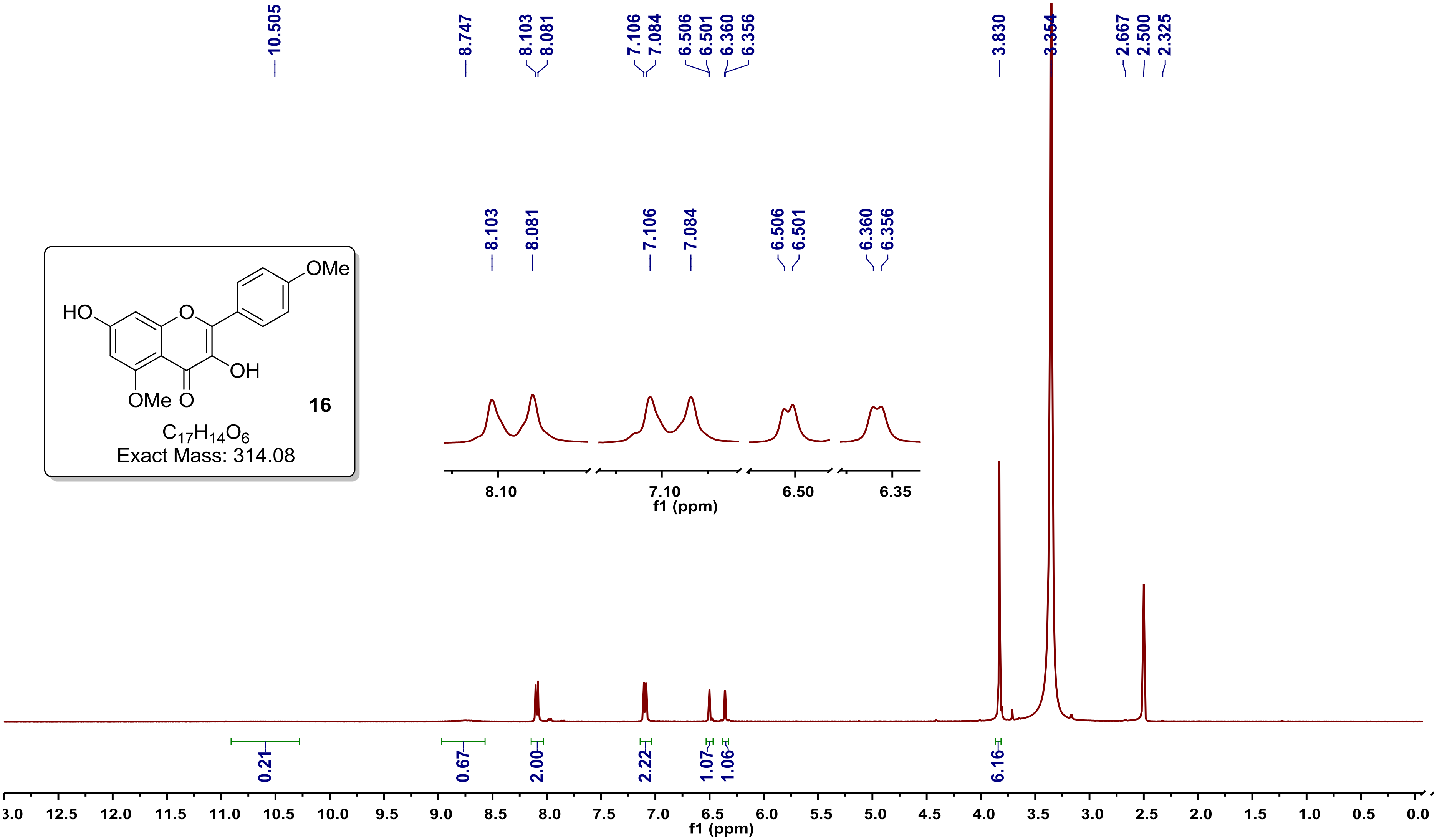
10.505
8.747
8.103
8.081
7.106
7.084
6.506
6.501
6.360
6.356
3.830
3.354
2.667
2.500
2.325
OMe
HO
O
OH
OMe O
16
$C_{17}H_{14}O_6$
Exact Mass: 314.08
8.10
7.10
6.50
6.35
f1 (ppm)
0.21
0.67
2.00
2.22
1.07
1.06
6.16
f1 (ppm)

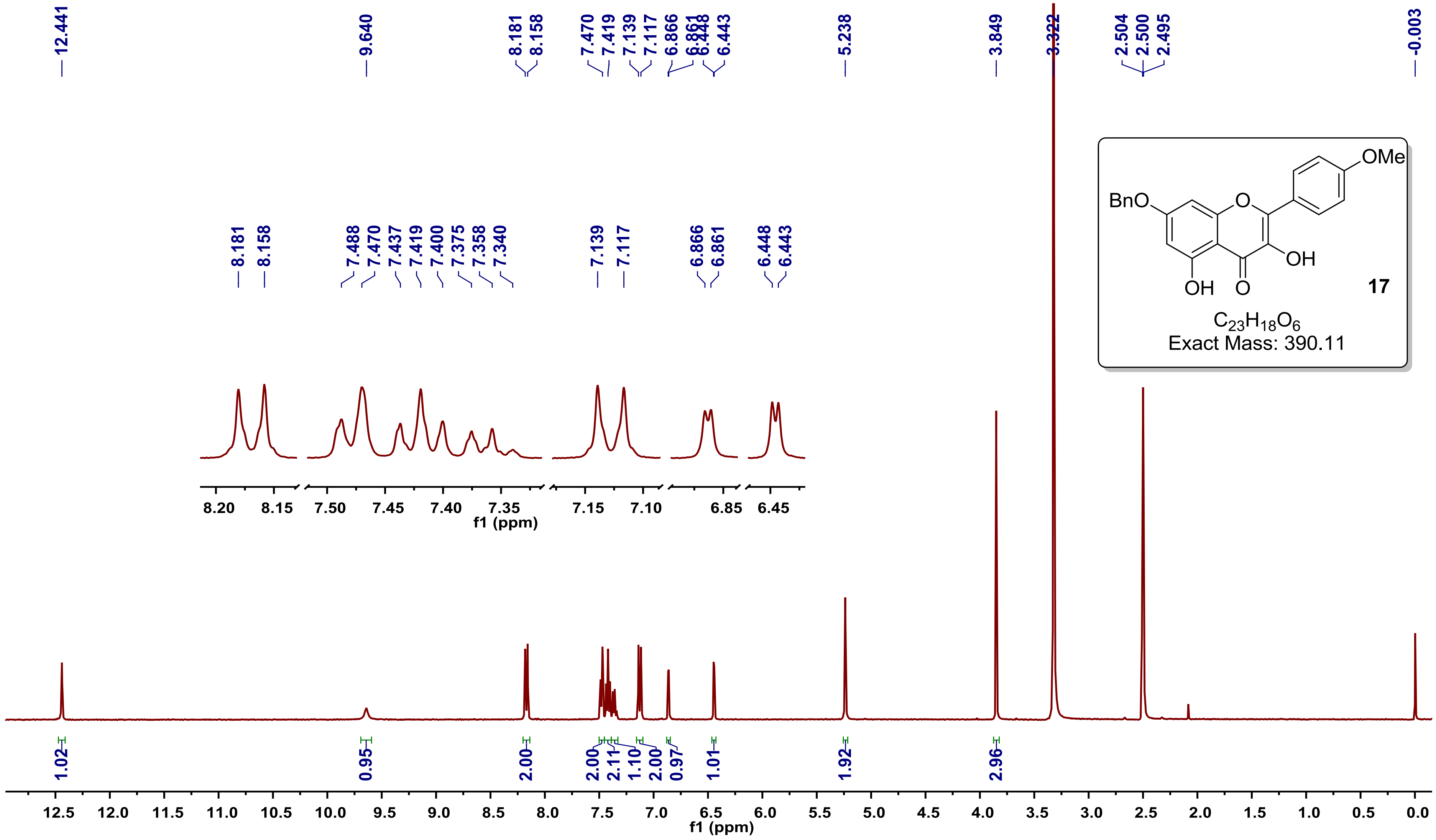
12.441
9.640
8.181
8.158
7.470
7.419
7.139
7.117
6.866
6.861
6.448
6.443
5.238
3.849
3.322
2.504
2.500
2.495
-0.003
8.181
8.158
7.488
7.470
7.437
7.419
7.400
7.375
7.358
7.340
7.139
7.117
6.866
6.861
6.448
6.443
8.20 8.15 7.50 7.45 7.40 7.35 7.15 7.10 6.85 6.45
f1 (ppm)
BnO
O
OMe
OH
OH
O
17
$C_{23}H_{18}O_6$
Exact Mass: 390.11
1.02
0.95
2.00
2.00
2.11
1.10
2.00
0.97
1.01
1.92
2.96
12.5 12.0 11.5 11.0 10.5 10.0 9.5 9.0 8.5 8.0 7.5 7.0 6.5 6.0 5.5 5.0 4.5 4.0 3.5 3.0 2.5 2.0 1.5 1.0 0.5 0.0
f1 (ppm)

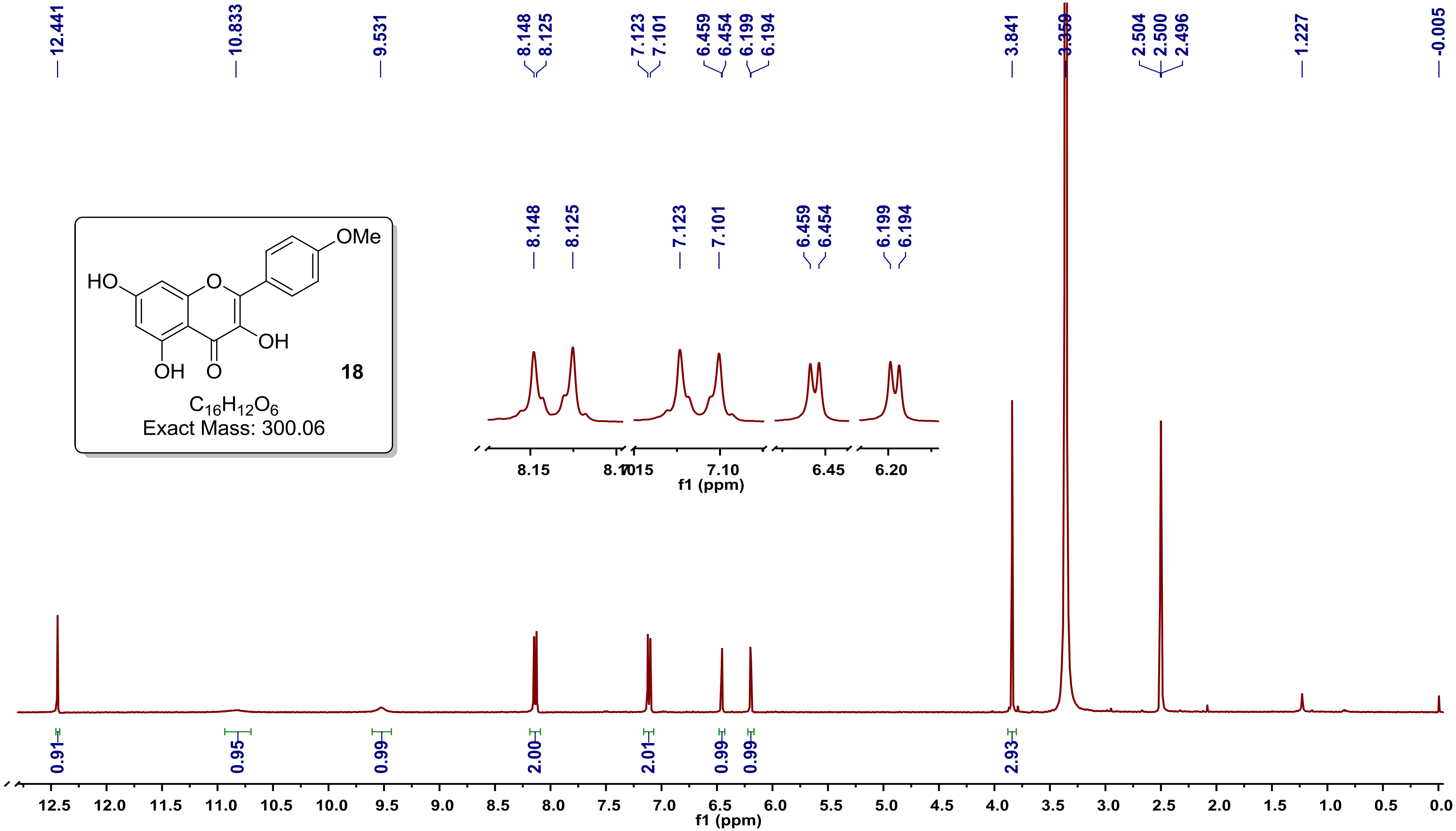
12.441
10.833
9.531
8.148
8.125
7.123
7.101
6.459
6.454
6.199
6.194
3.841
3.359
2.504
2.500
2.496
1.227
-0.005
HO
O
OMe
OH
OH
O
18
$C_{16}H_{12}O_6$
Exact Mass: 300.06
8.15
7.10
6.45
6.20
f1 (ppm)
0.91
0.95
0.99
2.00
2.01
0.99
0.99
2.93
12.5
12.0
11.5
11.0
10.5
10.0
9.5
9.0
8.5
8.0
7.5
7.0
6.5
6.0
5.5
5.0
4.5
4.0
3.5
3.0
2.5
2.0
1.5
1.0
0.5
0.0
f1 (ppm)

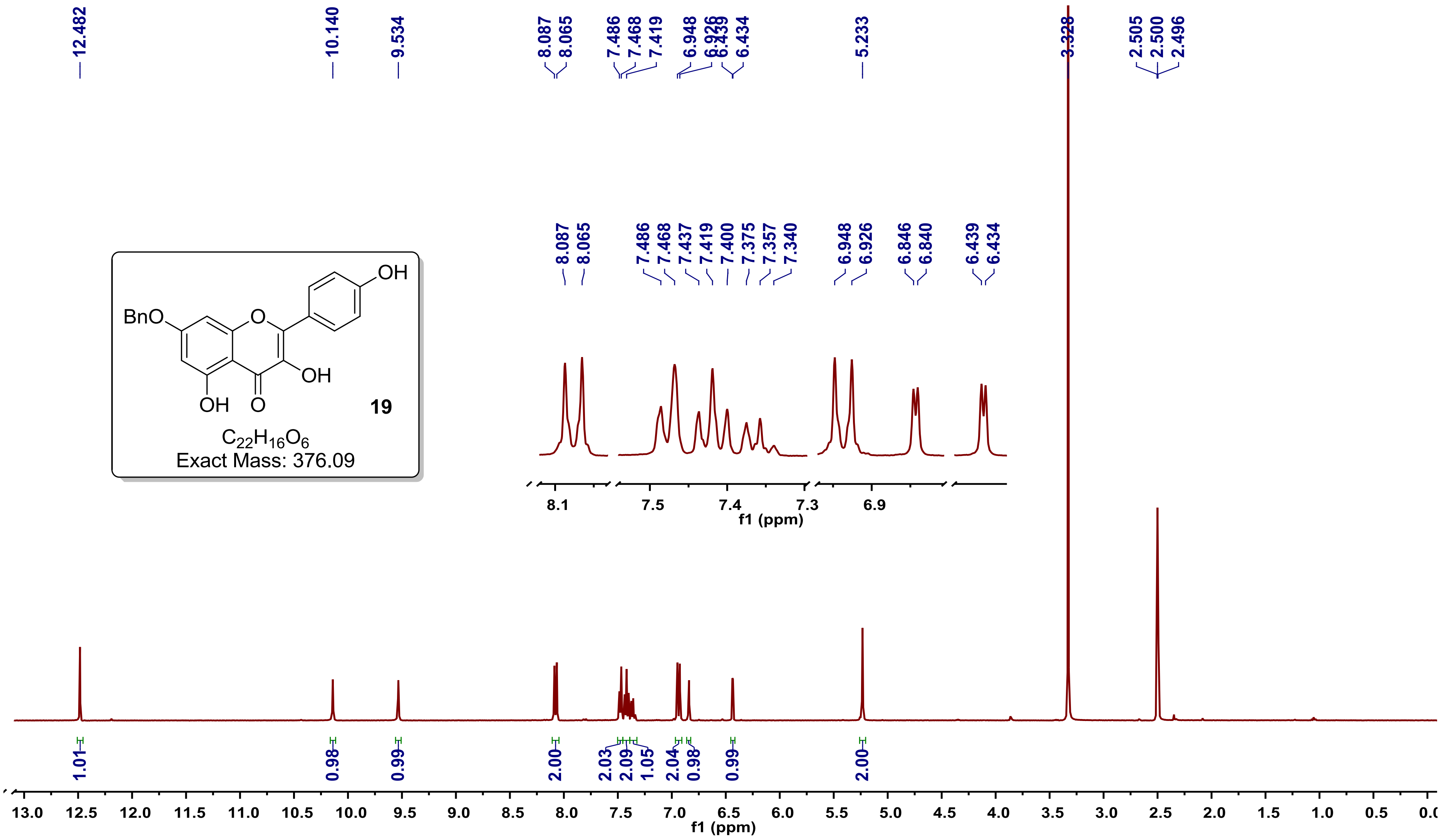

12.482
10.140
9.534
8.087
8.065
7.486
7.468
7.437
7.419
7.400
7.375
7.357
7.340
6.948
6.926
6.846
6.840
6.439
6.434
5.233
3.328
2.505
2.500
2.496
BnO
O
OH
OH
OH
O
19
$C_{22}H_{16}O_6$
Exact Mass: 376.09
1.01
0.98
0.99
2.00
2.03
2.09
1.05
2.04
0.98
0.99
2.00
f1 (ppm)

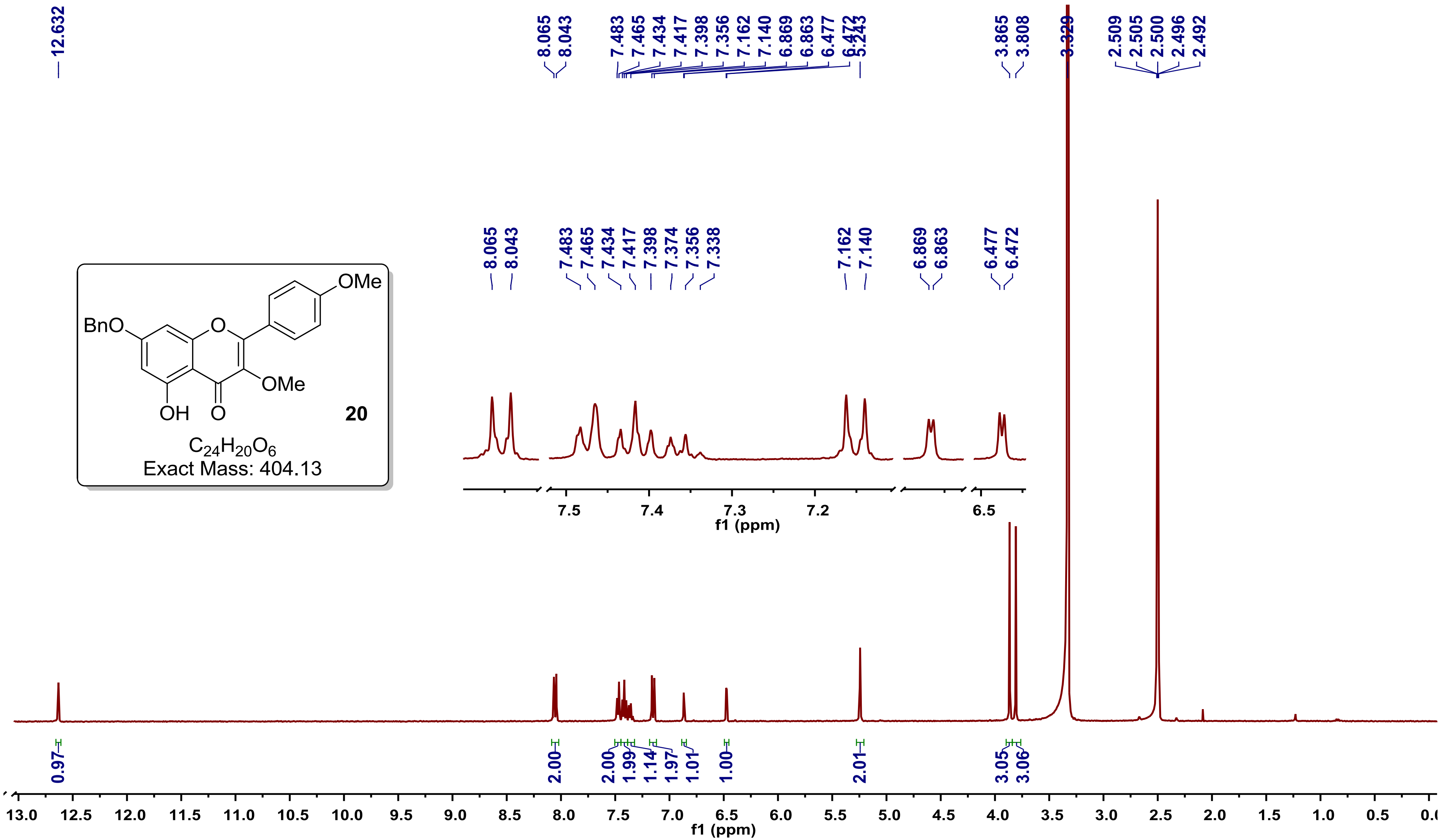

12.632
8.065
8.043
7.483
7.465
7.434
7.417
7.398
7.356
7.162
7.140
6.869
6.863
6.477
3.865
3.808
2.509
2.505
2.500
2.496
2.492
7.374
7.338
6.472
BnO
O
OMe
OMe
OH
O
20
$C_{24}H_{20}O_6$
Exact Mass: 404.13
0.97
2.00
2.00
1.99
1.14
1.97
1.01
1.00
2.01
3.05
3.06
f1 (ppm)

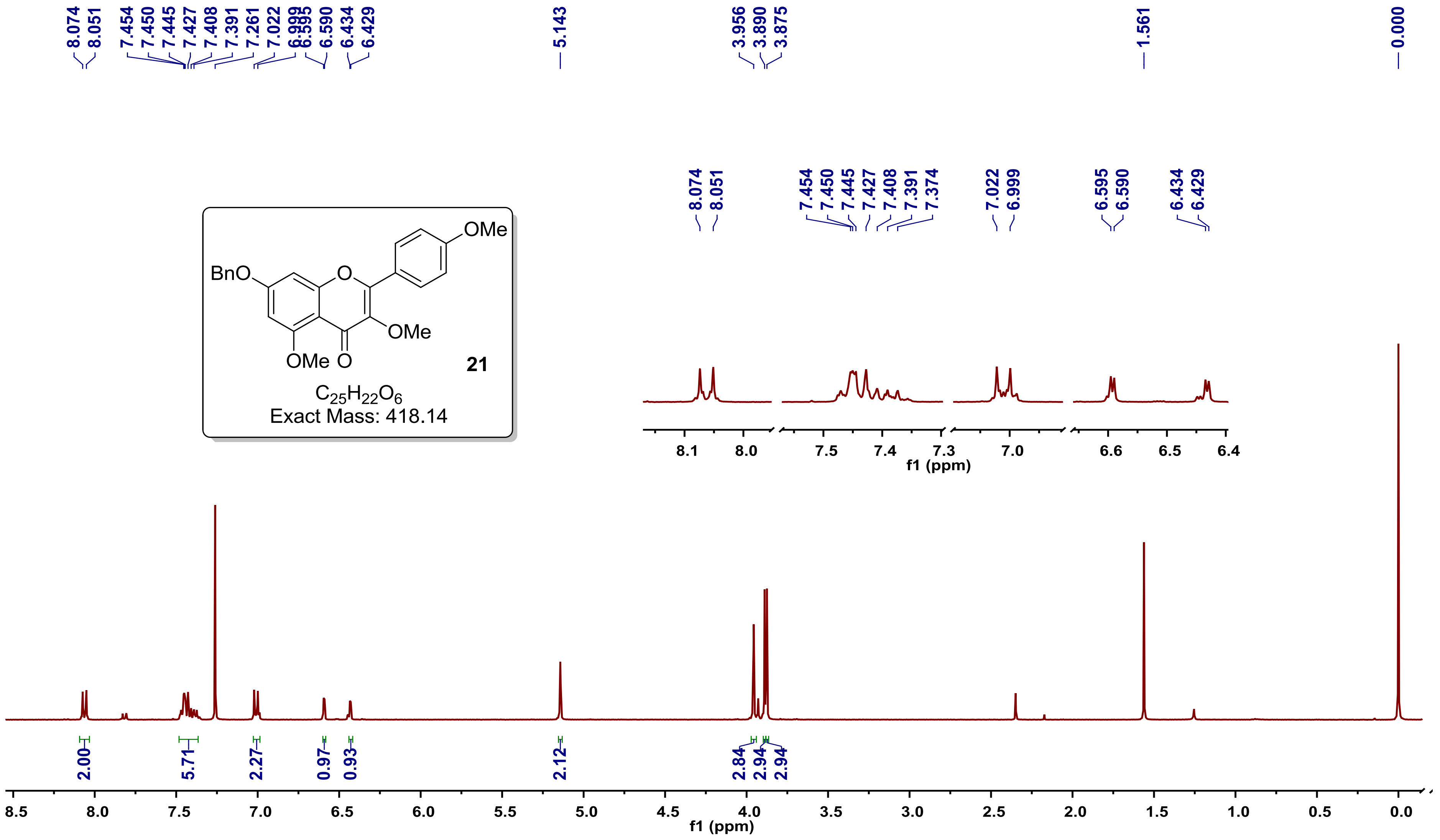
8.074
8.051
7.454
7.450
7.445
7.427
7.408
7.391
7.261
7.022
6.999
6.595
6.590
6.434
6.429
5.143
3.956
3.890
3.875
1.561
0.000
BnO
O
OMe
OMe
OMe O
21
$C_{25}H_{22}O_6$
Exact Mass: 418.14
7.374
2.00
5.71
2.27
0.97
0.93
2.12
2.84
2.94
2.94
f1 (ppm)

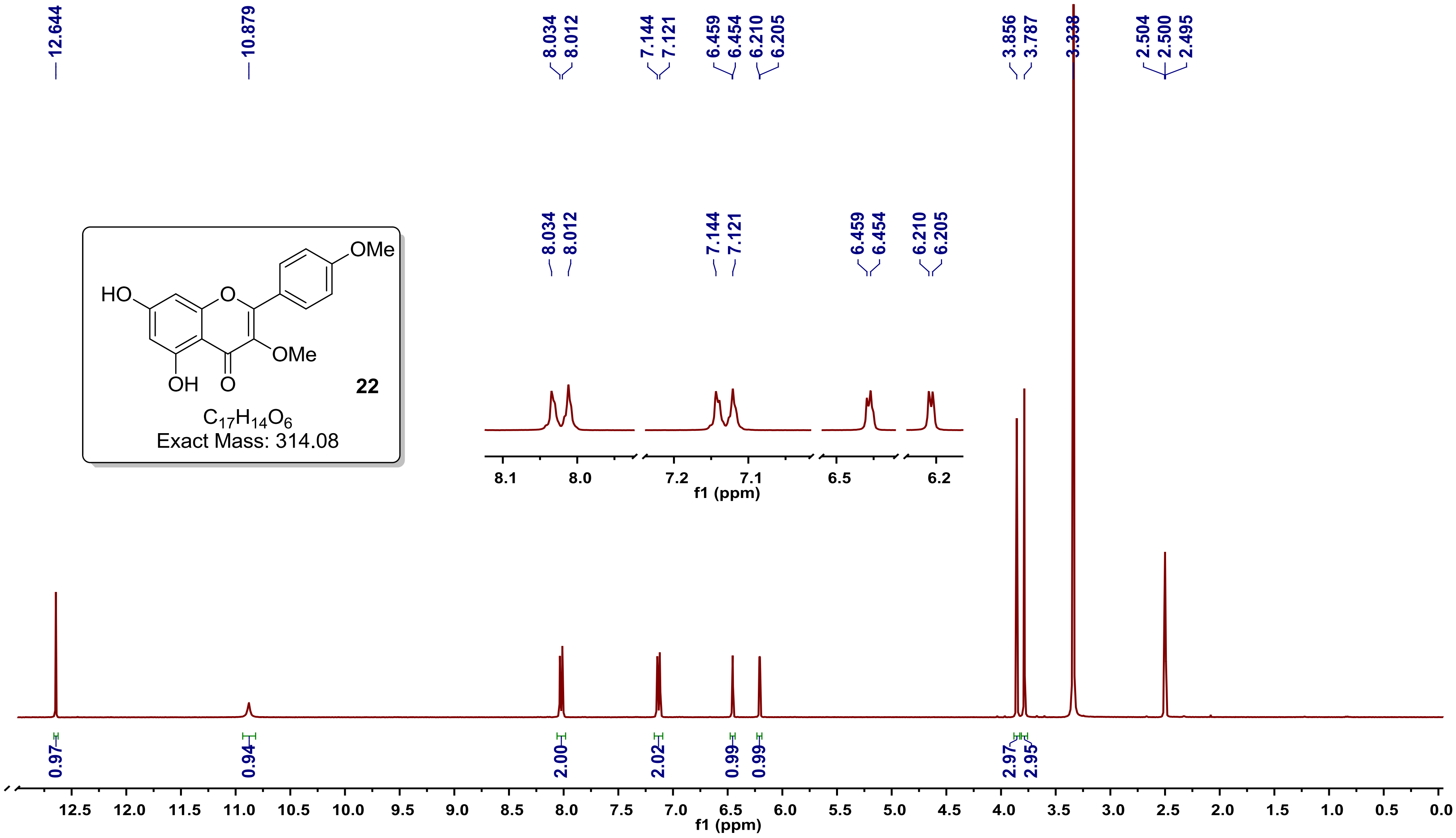
12.644
10.879
8.034
8.012
7.144
7.121
6.459
6.454
6.210
6.205
3.856
3.787
3.338
2.504
2.500
2.495
HO
O
OMe
OMe
OH
O
22
$C_{17}H_{14}O_6$
Exact Mass: 314.08
0.97
0.94
2.00
2.02
0.99
0.99
2.97
2.95
f1 (ppm)

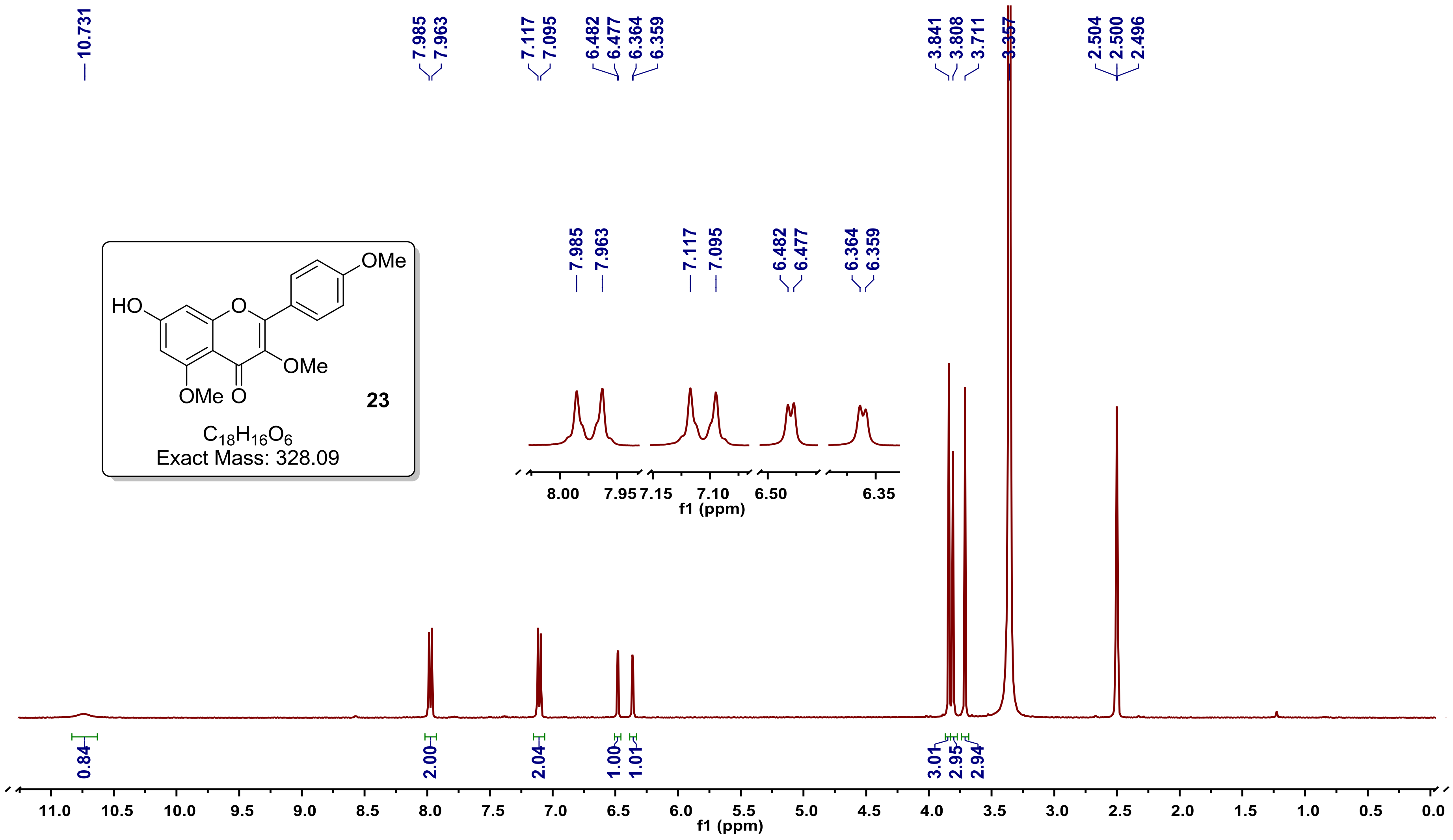
10.731
7.985
7.963
7.117
7.095
6.482
6.477
6.364
6.359
3.841
3.808
3.711
3.357
2.504
2.500
2.496
HO
O
OMe
OMe
OMe O
23
$C_{18}H_{16}O_6$
Exact Mass: 328.09
8.00
7.95
7.15
7.10
6.50
6.35
f1 (ppm)
0.84
2.00
2.04
1.00
1.01
3.01
2.95
2.94
11.0
10.5
10.0
9.5
9.0
8.5
8.0
7.5
7.0
6.5
6.0
5.5
5.0
4.5
4.0
3.5
3.0
2.5
2.0
1.5
1.0
0.5
0.0
f1 (ppm)

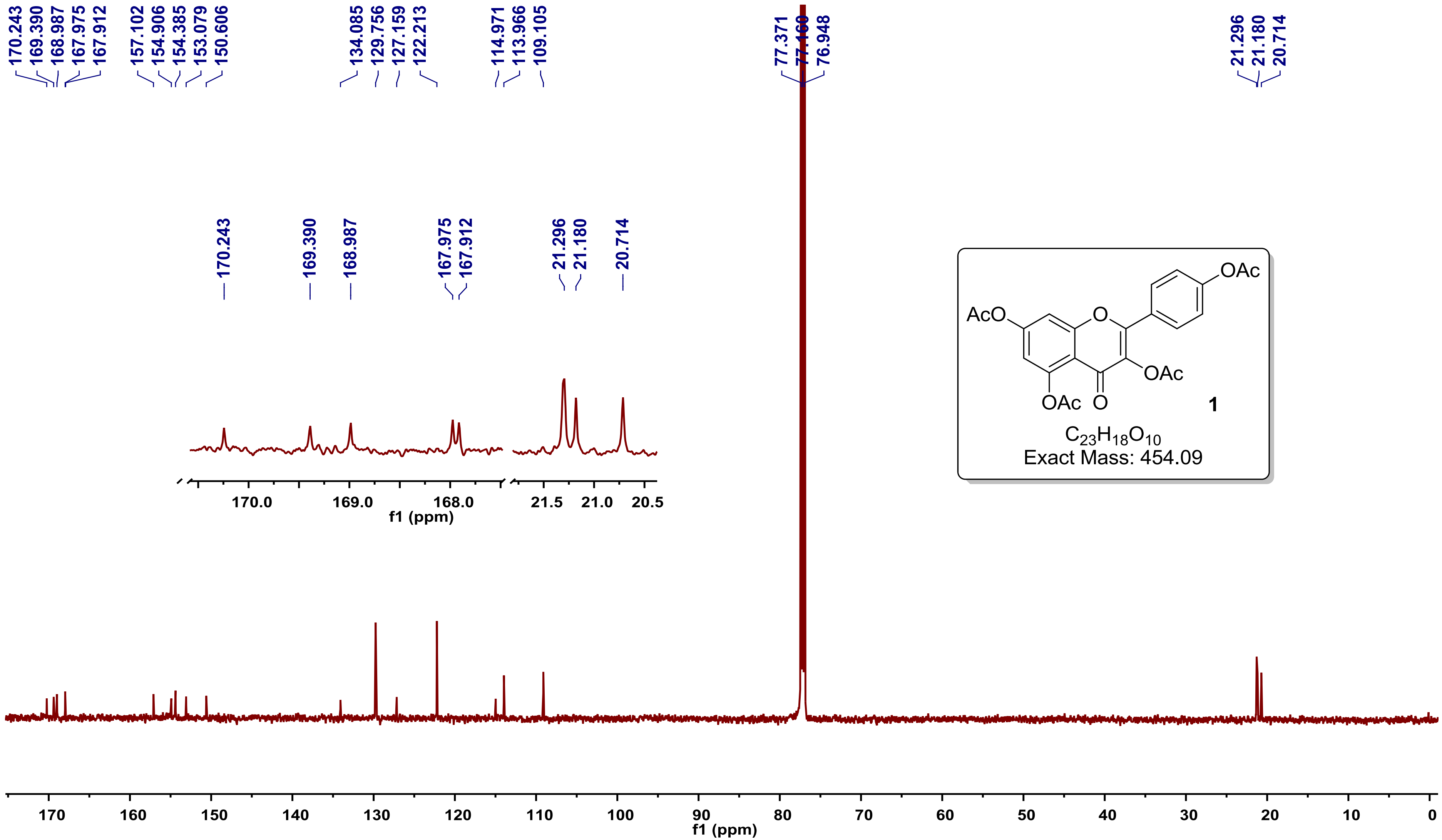
170.243
169.390
168.987
167.975
167.912
157.102
154.906
154.385
153.079
150.606
134.085
129.756
127.159
122.213
114.971
113.966
109.105
77.371
77.160
76.948
21.296
21.180
20.714
170.0
169.0
168.0
21.5
21.0
20.5
f1 (ppm)
AcO
O
OAc
OAc
OAc
O
1
$C_{23}H_{18}O_{10}$
Exact Mass: 454.09
170
160
150
140
130
120
110
100
90
80
70
60
50
40
30
20
10
0
f1 (ppm)

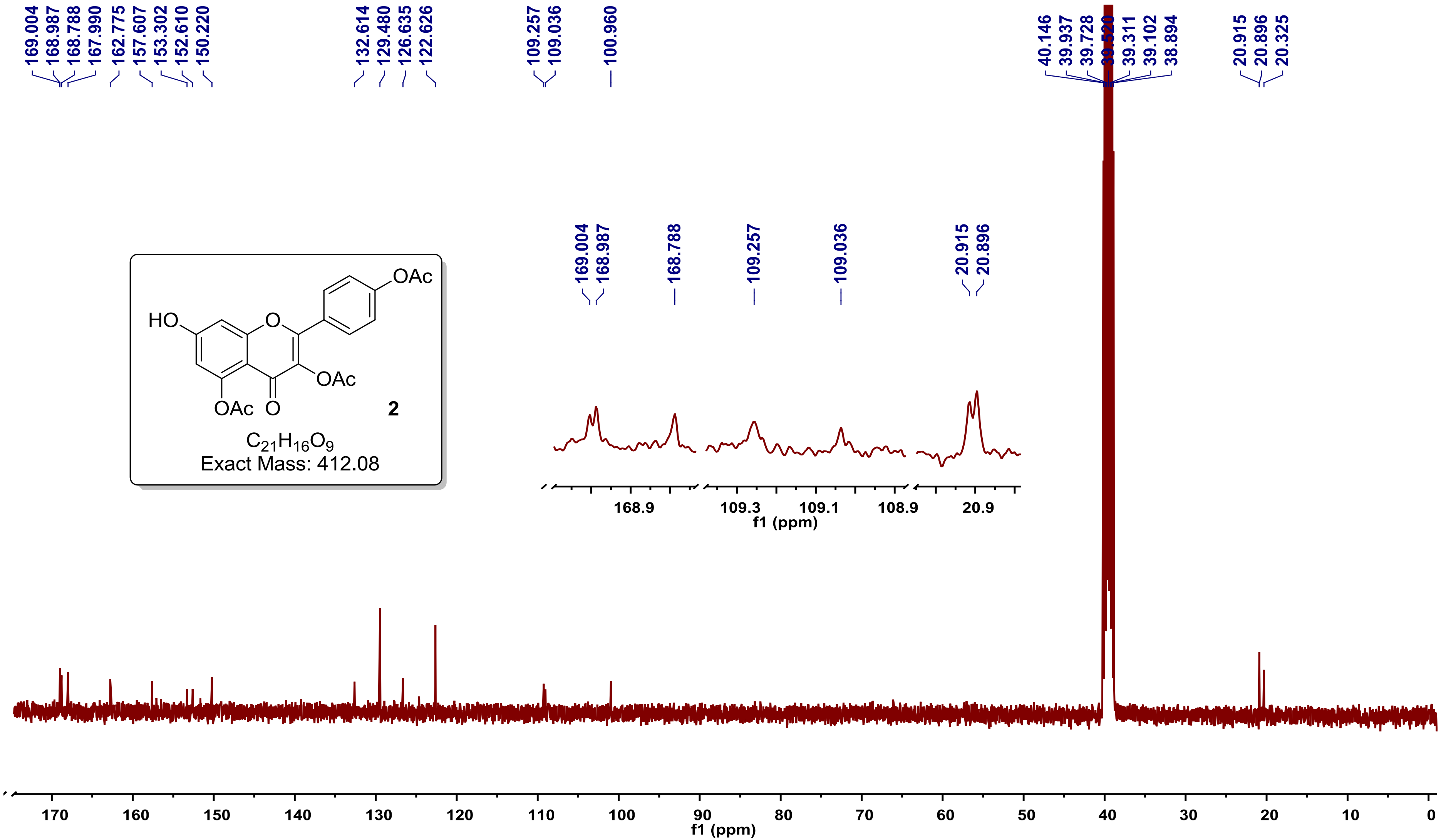
169.004
168.987
168.788
167.990
162.775
157.607
153.302
152.610
150.220
132.614
129.480
126.635
122.626
109.257
109.036
100.960
40.146
39.937
39.728
39.520
39.311
39.102
38.894
20.915
20.896
20.325
HO
O
OAc
OAc
OAc
O
2
C21H16O9
Exact Mass: 412.08
168.9
109.3
109.1
108.9
20.9
f1 (ppm)
f1 (ppm)

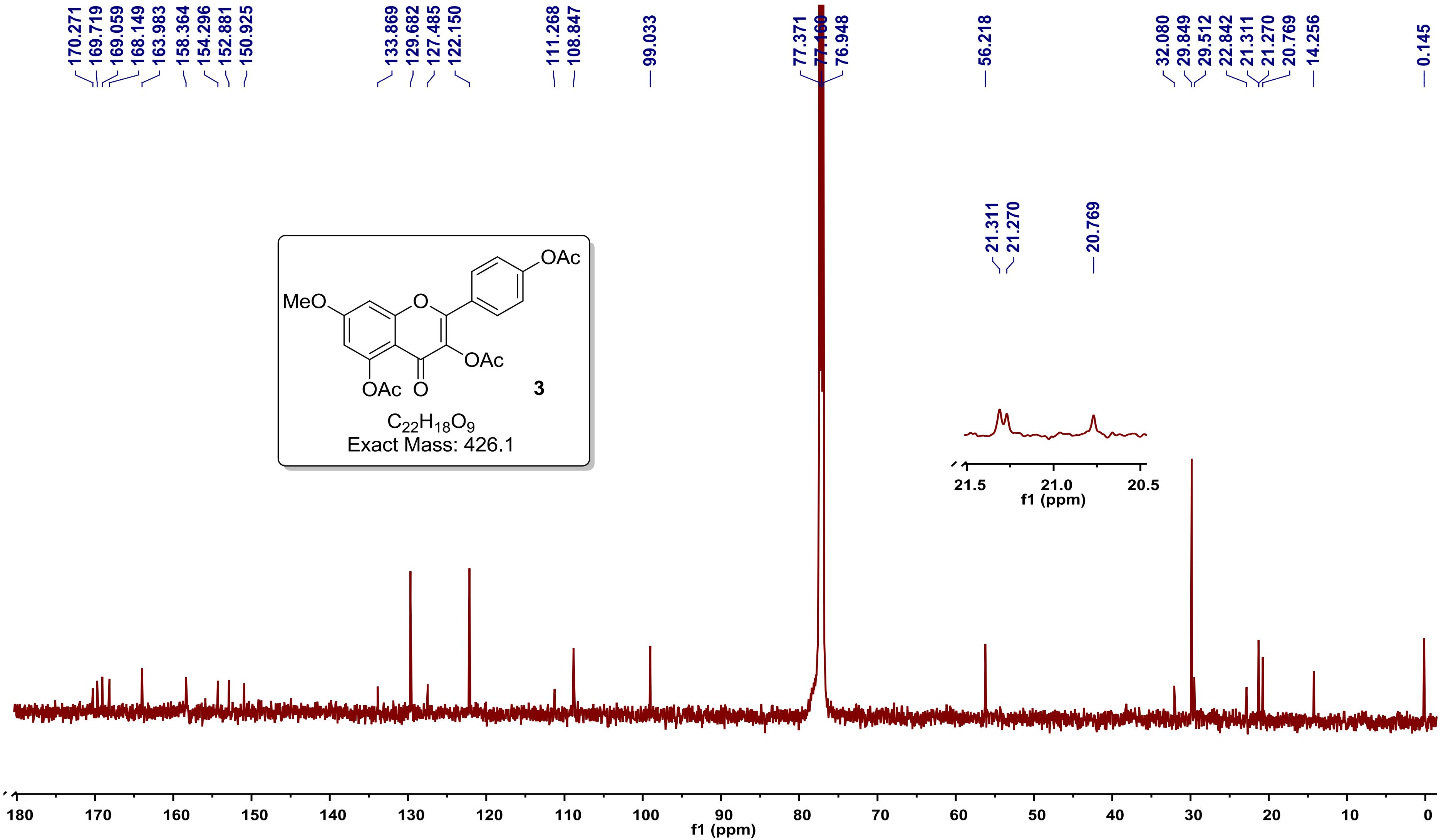
170.271
169.719
169.059
168.149
163.983
158.364
154.296
152.881
150.925
133.869
129.682
127.485
122.150
111.268
108.847
99.033
77.371
77.160
76.948
56.218
32.080
29.849
29.512
22.842
21.311
21.270
20.769
14.256
0.145
MeO
O
OAc
OAc
OAc
O
3
$C_{22}H_{18}O_9$
Exact Mass: 426.1
21.311
21.270
20.769
21.5
21.0
20.5
f1 (ppm)
180
170
160
150
140
130
120
110
100
90
80
70
60
50
40
30
20
10
0
f1 (ppm)

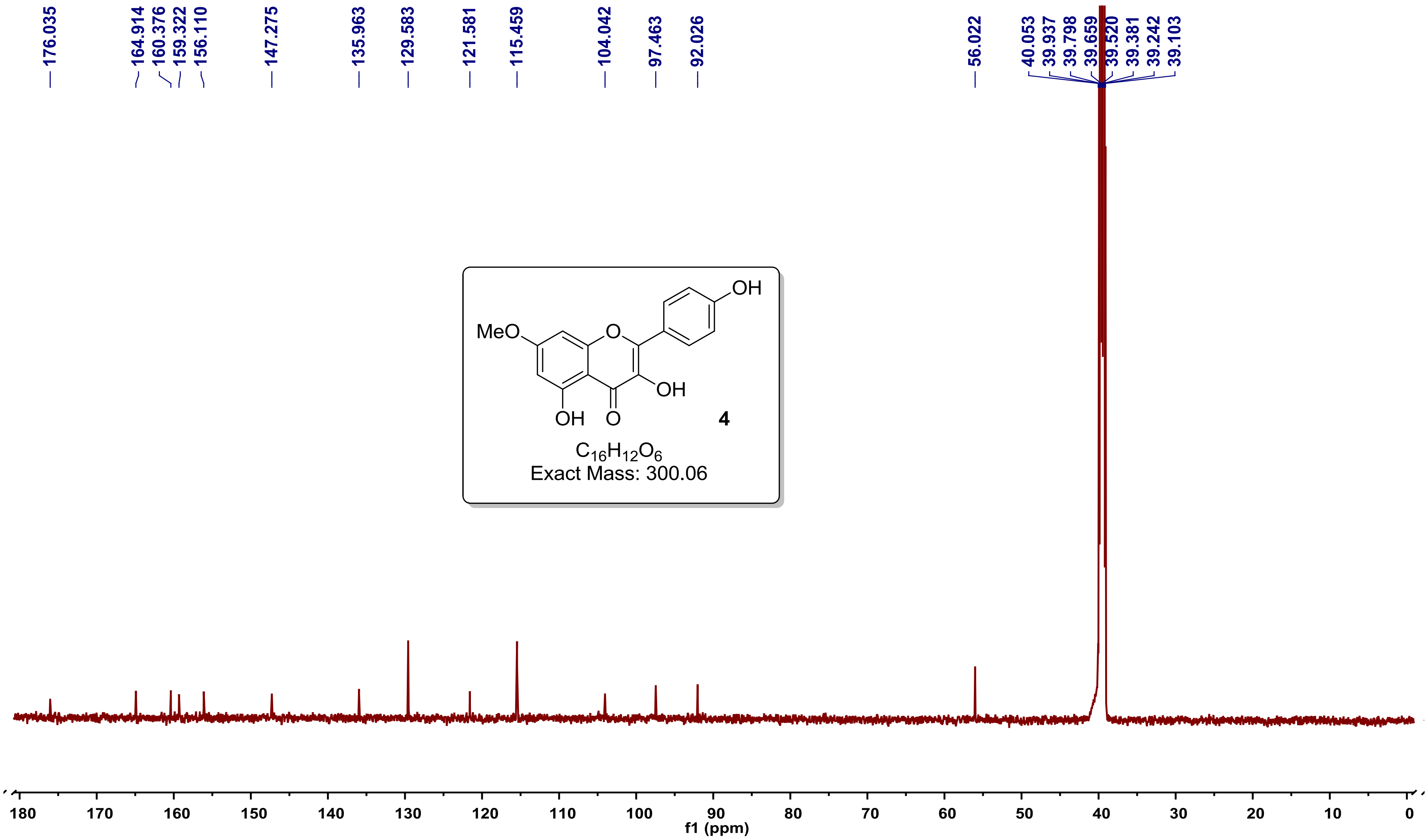
176.035
164.914
160.376
159.322
156.110
147.275
135.963
129.583
121.581
115.459
104.042
97.463
92.026
56.022
40.053
39.937
39.798
39.659
39.520
39.381
39.242
39.103
MeO
O
OH
OH
OH
O
4
$C_{16}H_{12}O_6$
Exact Mass: 300.06
180
170
160
150
140
130
120
110
100
90
80
70
60
50
40
30
20
10
0
f1 (ppm)

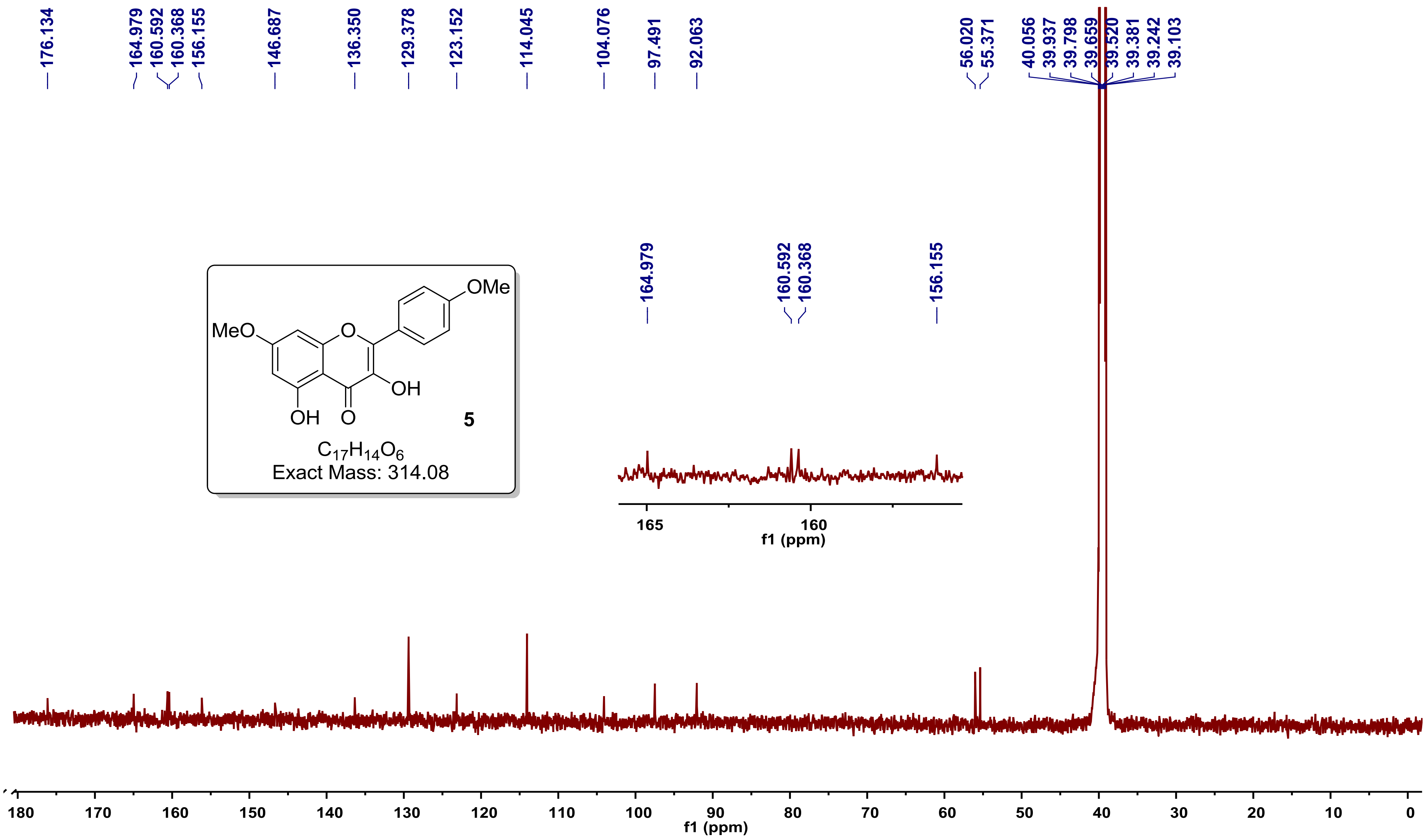
176.134
164.979
160.592
160.368
156.155
146.687
136.350
129.378
123.152
114.045
104.076
97.491
92.063
56.020
55.371
40.056
39.937
39.798
39.659
39.520
39.381
39.242
39.103
MeO
O
OMe
OH
OH
O
5
$C_{17}H_{14}O_6$
Exact Mass: 314.08
165
160
f1 (ppm)
180
170
160
150
140
130
120
110
100
90
80
70
60
50
40
30
20
10
0
f1 (ppm)

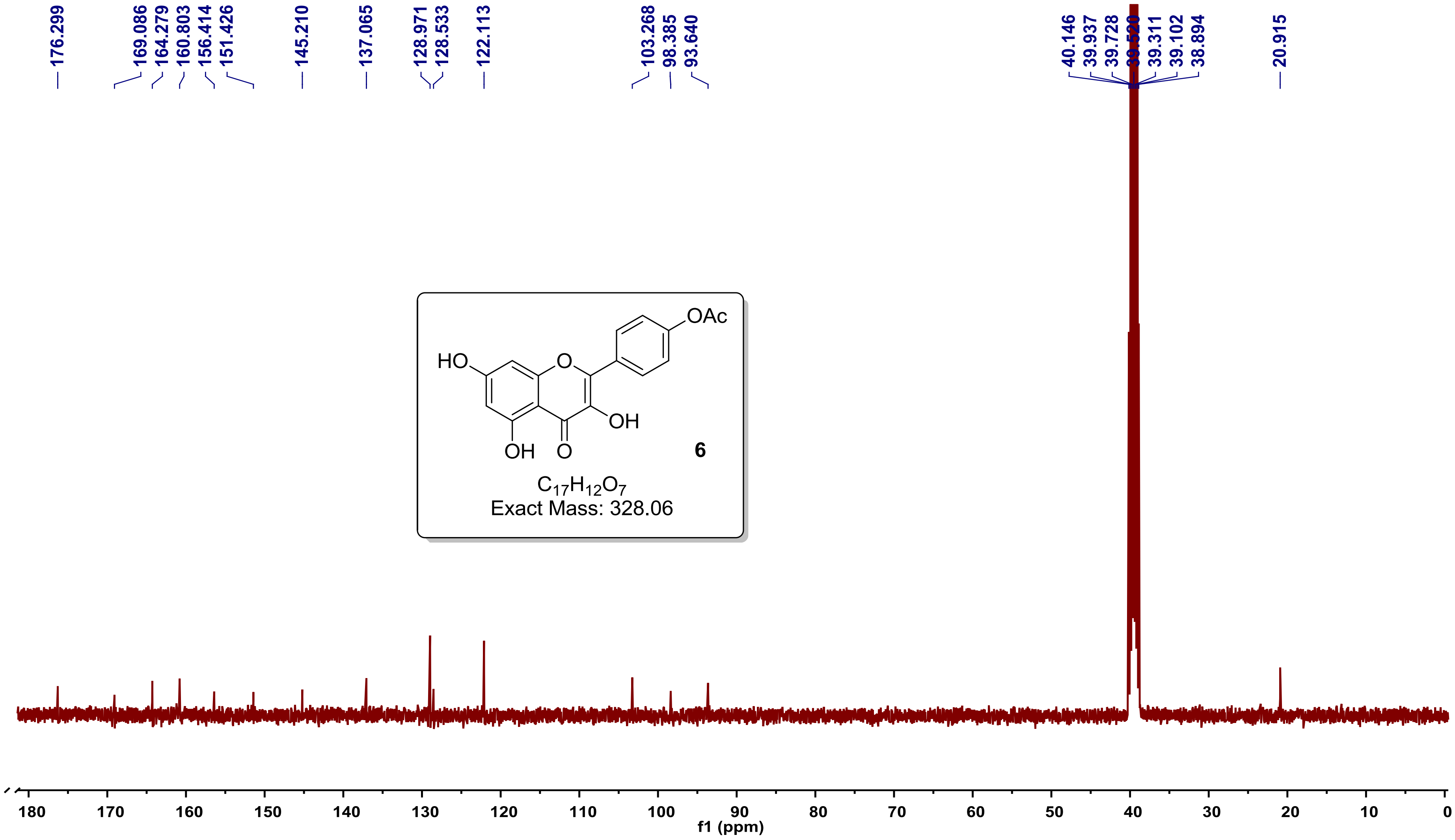

176.299
169.086
164.279
160.803
156.414
151.426
145.210
137.065
128.971
128.533
122.113
103.268
98.385
93.640
40.146
39.937
39.728
39.520
39.311
39.102
38.894
20.915
HO
O
OAc
OH
OH
O
6
$C_{17}H_{12}O_7$
Exact Mass: 328.06
180
170
160
150
140
130
120
110
100
90
80
70
60
50
40
30
20
10
0
f1 (ppm)

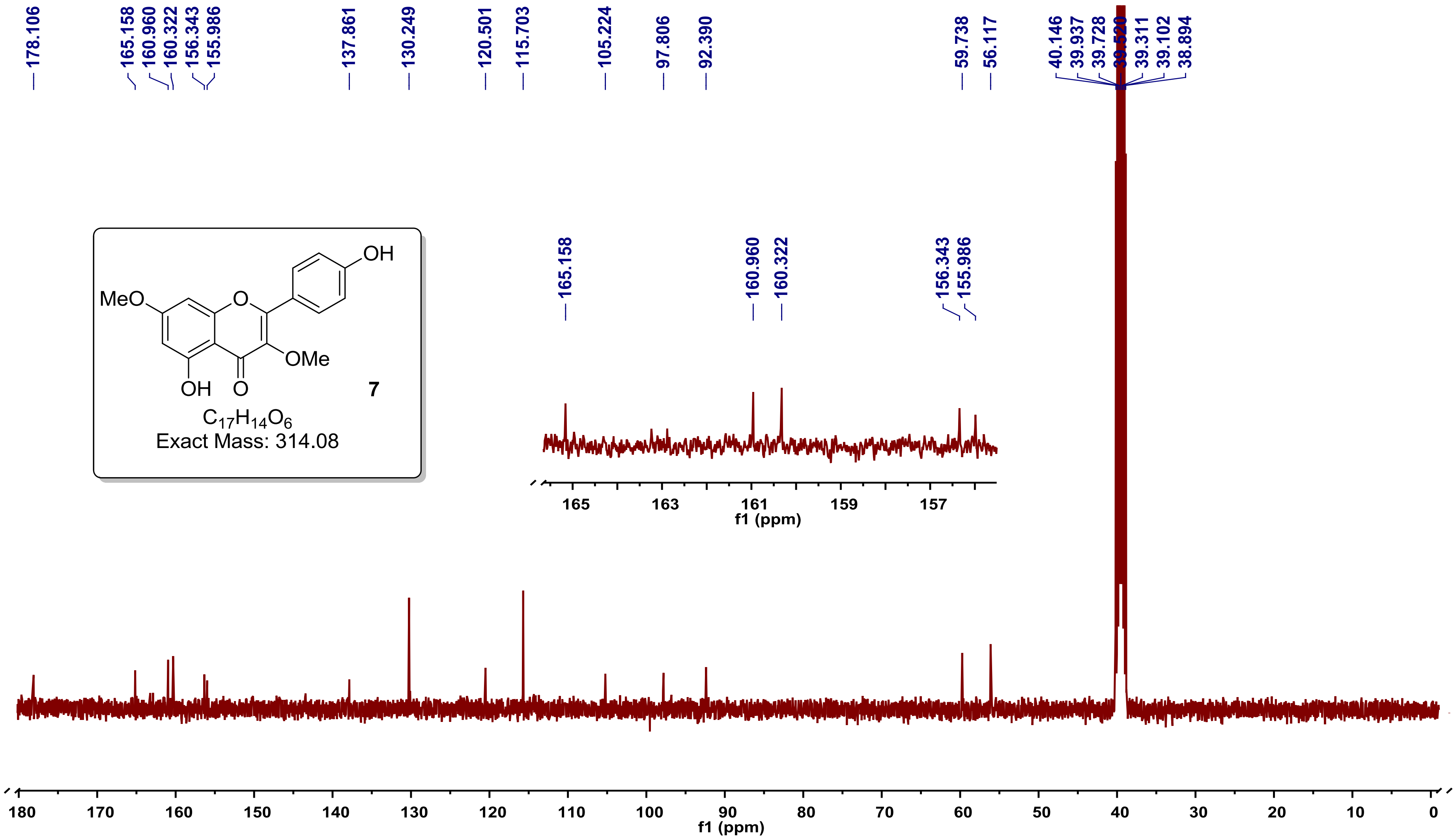
178.106
165.158
160.960
160.322
156.343
155.986
137.861
130.249
120.501
115.703
105.224
97.806
92.390
59.738
56.117
40.146
39.937
39.728
39.520
39.311
39.102
38.894
MeO
OH
OMe
OH
O
O
7
$C_{17}H_{14}O_6$
Exact Mass: 314.08
165
163
161
159
157
f1 (ppm)
180
170
160
150
140
130
120
110
100
90
80
70
60
50
40
30
20
10
0
f1 (ppm)

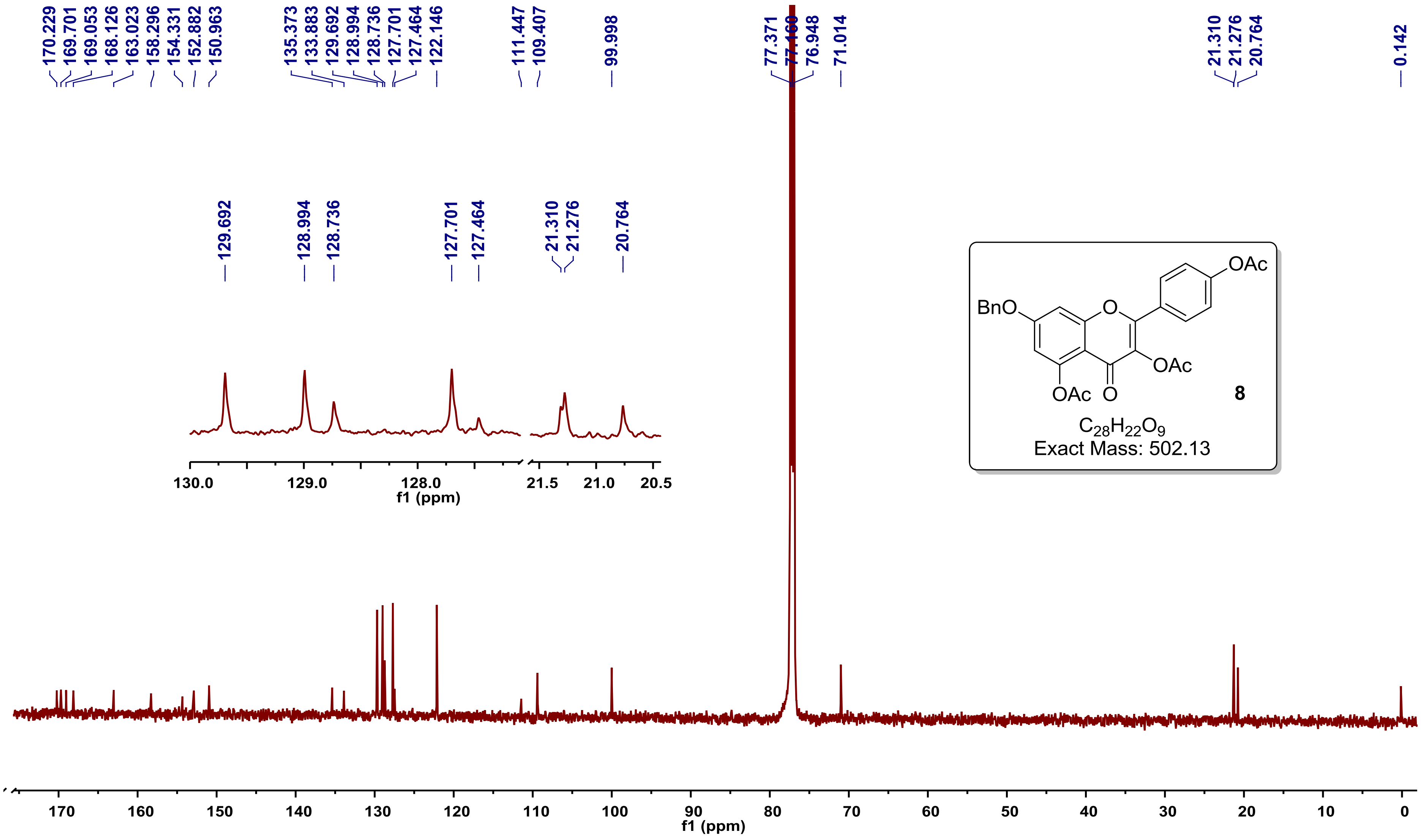
170.229
169.701
169.053
168.126
163.023
158.296
154.331
152.882
150.963
135.373
133.883
129.692
128.994
128.736
127.701
127.464
122.146
111.447
109.407
99.998
77.371
77.160
76.948
71.014
21.310
21.276
20.764
0.142
129.692
128.994
128.736
127.701
127.464
21.310
21.276
20.764
130.0
129.0
128.0
21.5
21.0
20.5
f1 (ppm)
BnO
O
OAc
OAc
OAc
O
8
$C_{28}H_{22}O_9$
Exact Mass: 502.13
170
160
150
140
130
120
110
100
90
80
70
60
50
40
30
20
10
0
f1 (ppm)

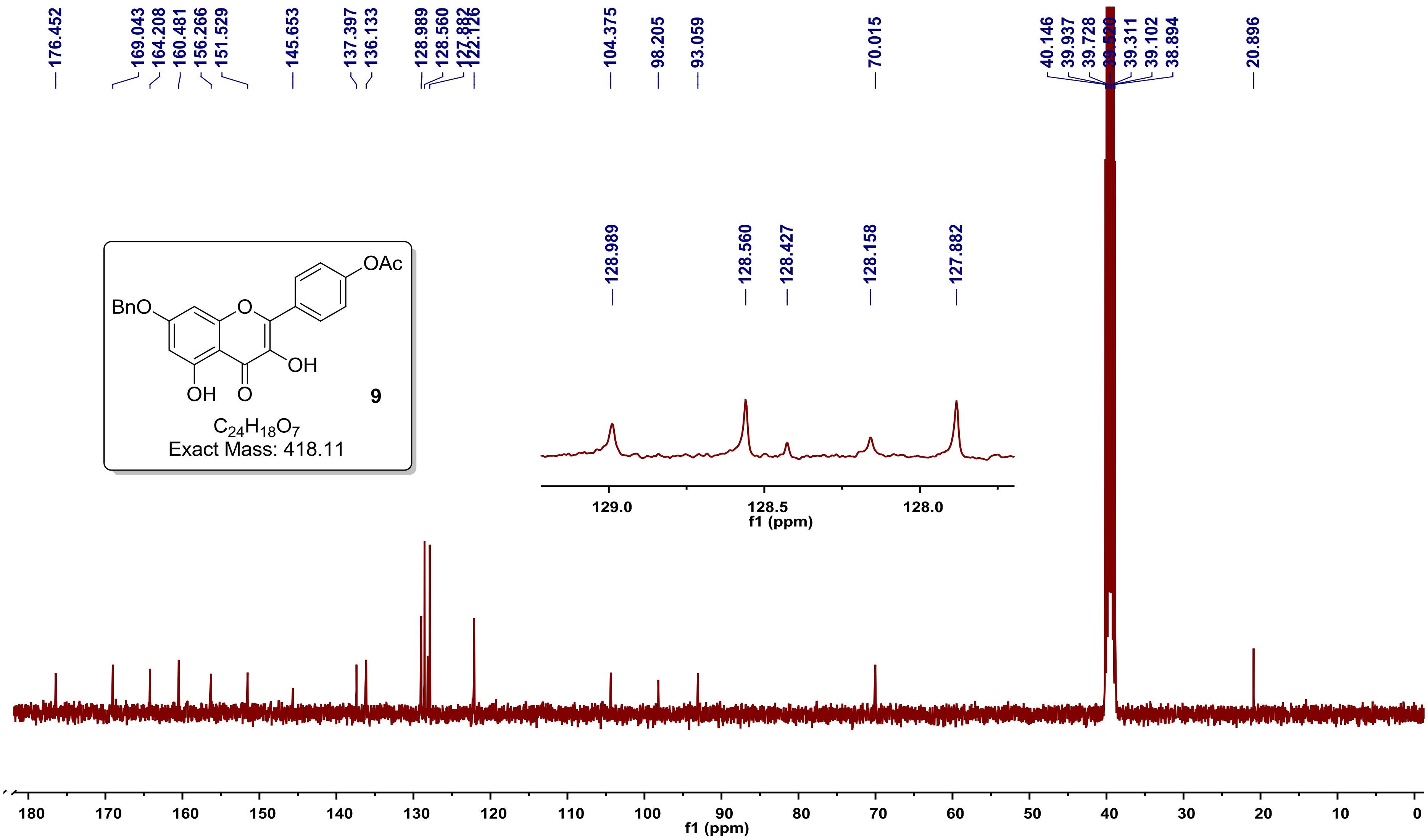
176.452
169.043
164.208
160.481
156.266
151.529
145.653
137.397
136.133
128.989
128.560
104.375
98.205
93.059
70.015
40.146
39.937
39.728
39.311
39.102
38.894
20.896
128.989
128.560
128.427
128.158
127.882
129.0
128.5
128.0
f1 (ppm)
OAc
BnO
O
OH
OH
O
9
C24H18O7
Exact Mass: 418.11
180
170
160
150
140
130
120
110
100
90
80
70
60
50
40
30
20
10
0
f1 (ppm)

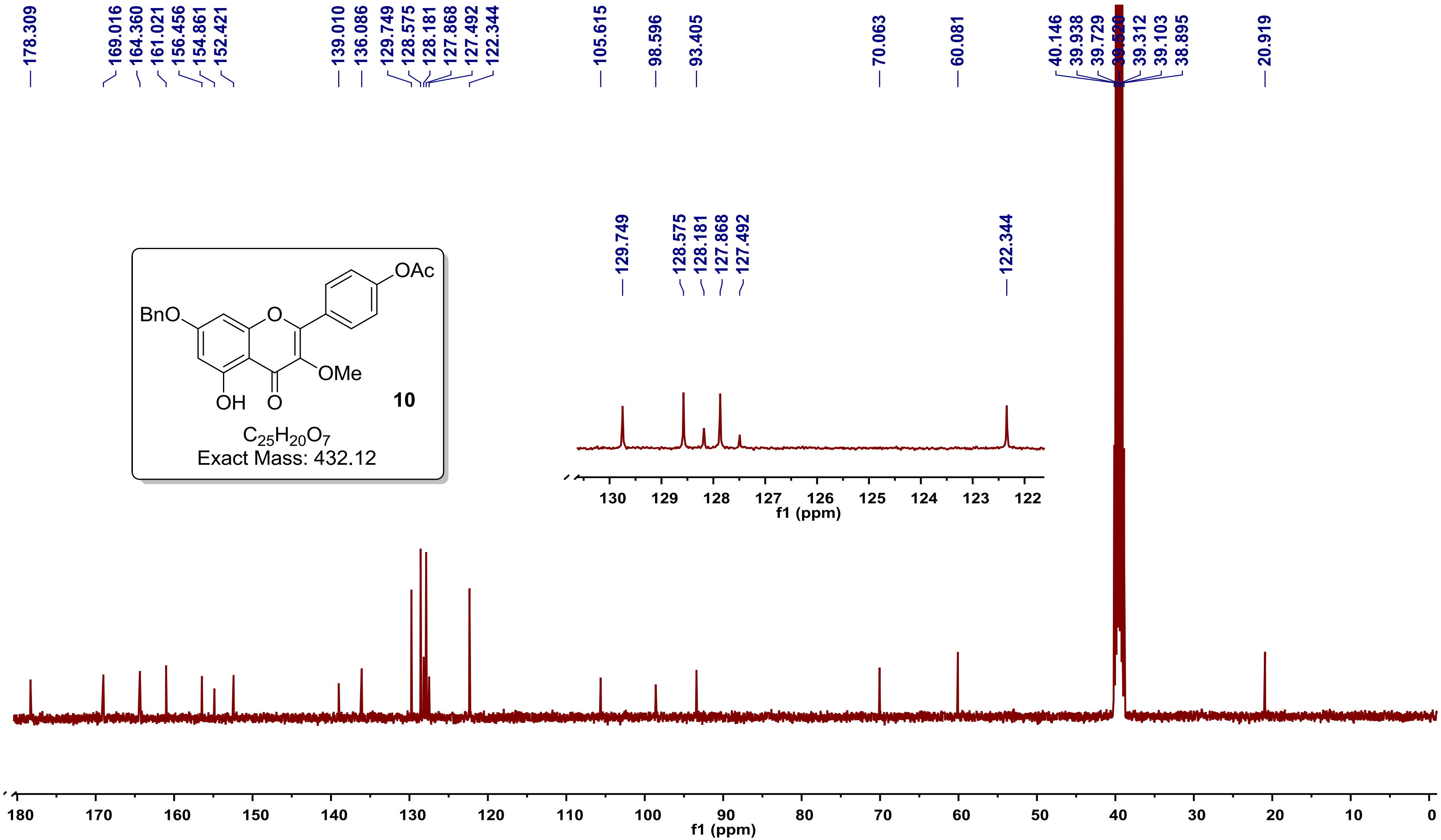
178.309
169.016
164.360
161.021
156.456
154.861
152.421
139.010
136.086
129.749
128.575
128.181
127.868
127.492
122.344
105.615
98.596
93.405
70.063
60.081
40.146
39.938
39.729
39.312
39.103
38.895
20.919
BnO
OAc
OMe
OH
O
O
10
C25H20O7
Exact Mass: 432.12
f1 (ppm)

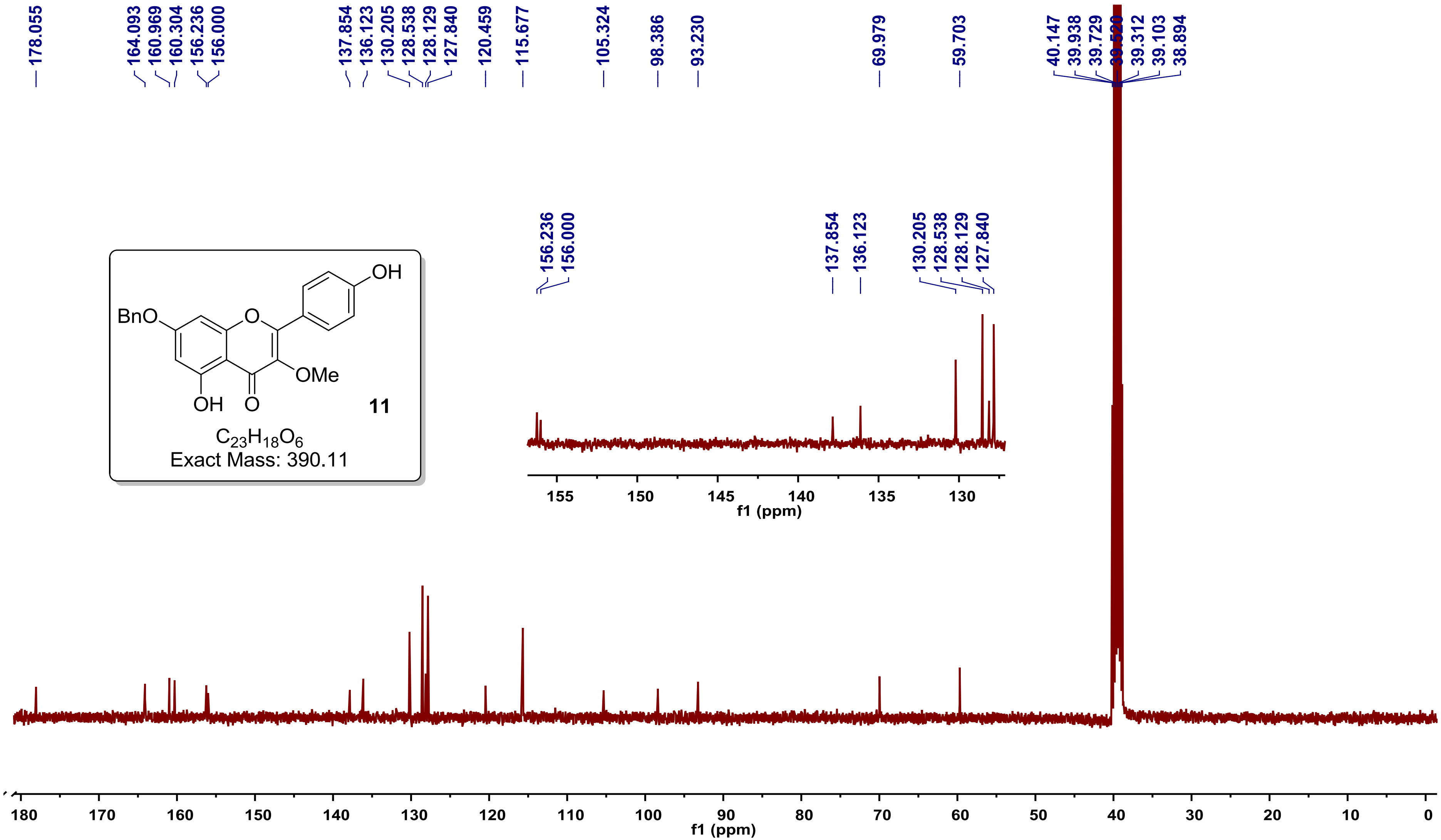

178.055
164.093
160.969
160.304
156.236
156.000
137.854
136.123
130.205
128.538
128.129
127.840
120.459
115.677
105.324
98.386
93.230
69.979
59.703
40.147
39.938
39.729
39.520
39.312
39.103
38.894
BnO
OH
OMe
OH
O
O
11
$C_{23}H_{18}O_6$
Exact Mass: 390.11
f1 (ppm)

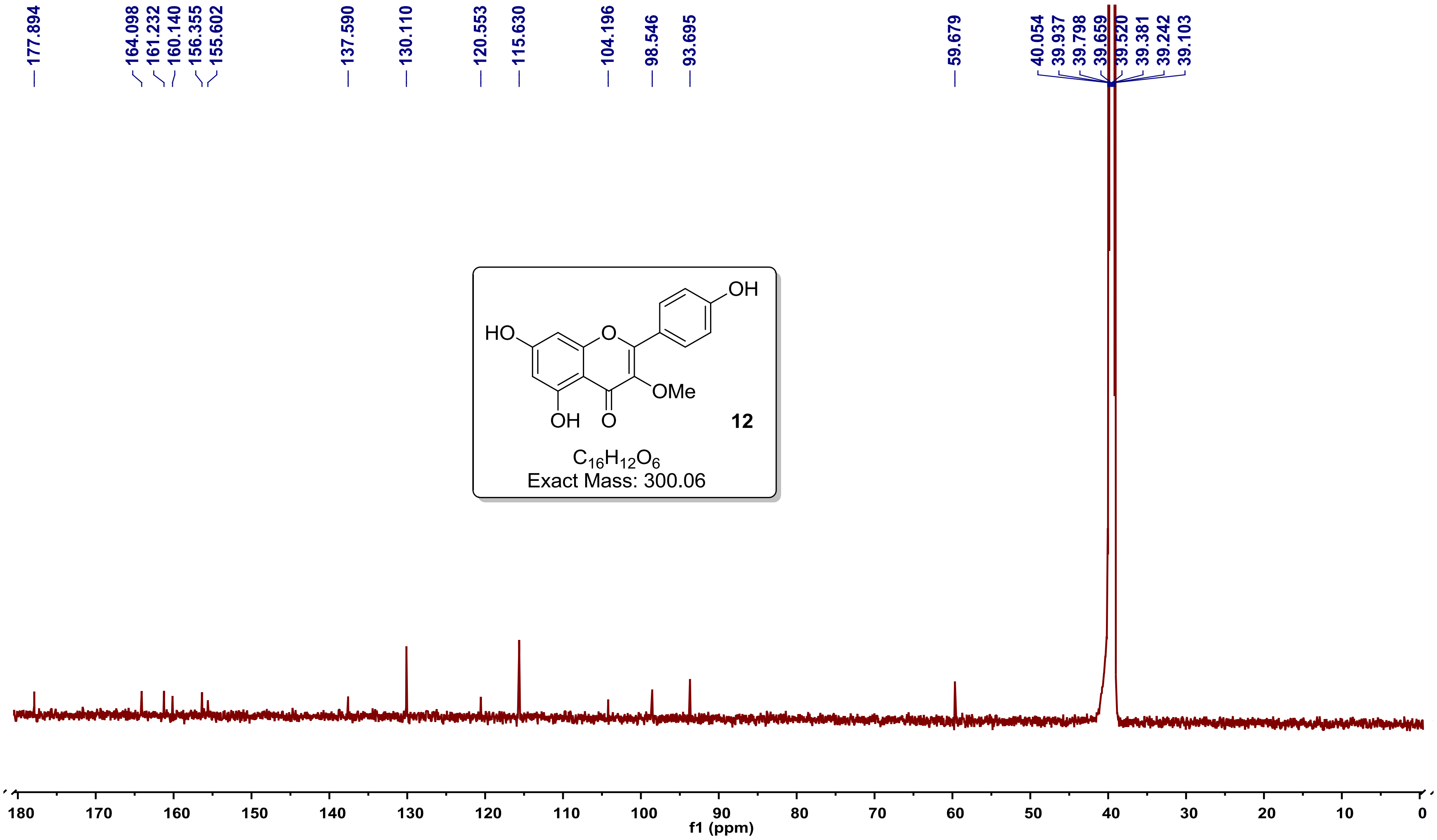
177.894
164.098
161.232
160.140
156.355
155.602
137.590
130.110
120.553
115.630
104.196
98.546
93.695
59.679
40.054
39.937
39.798
39.659
39.520
39.381
39.242
39.103
HO
O
OH
OMe
OH
O
12
$C_{16}H_{12}O_6$
Exact Mass: 300.06
180 170 160 150 140 130 120 110 100 90 80 70 60 50 40 30 20 10 0
f1 (ppm)

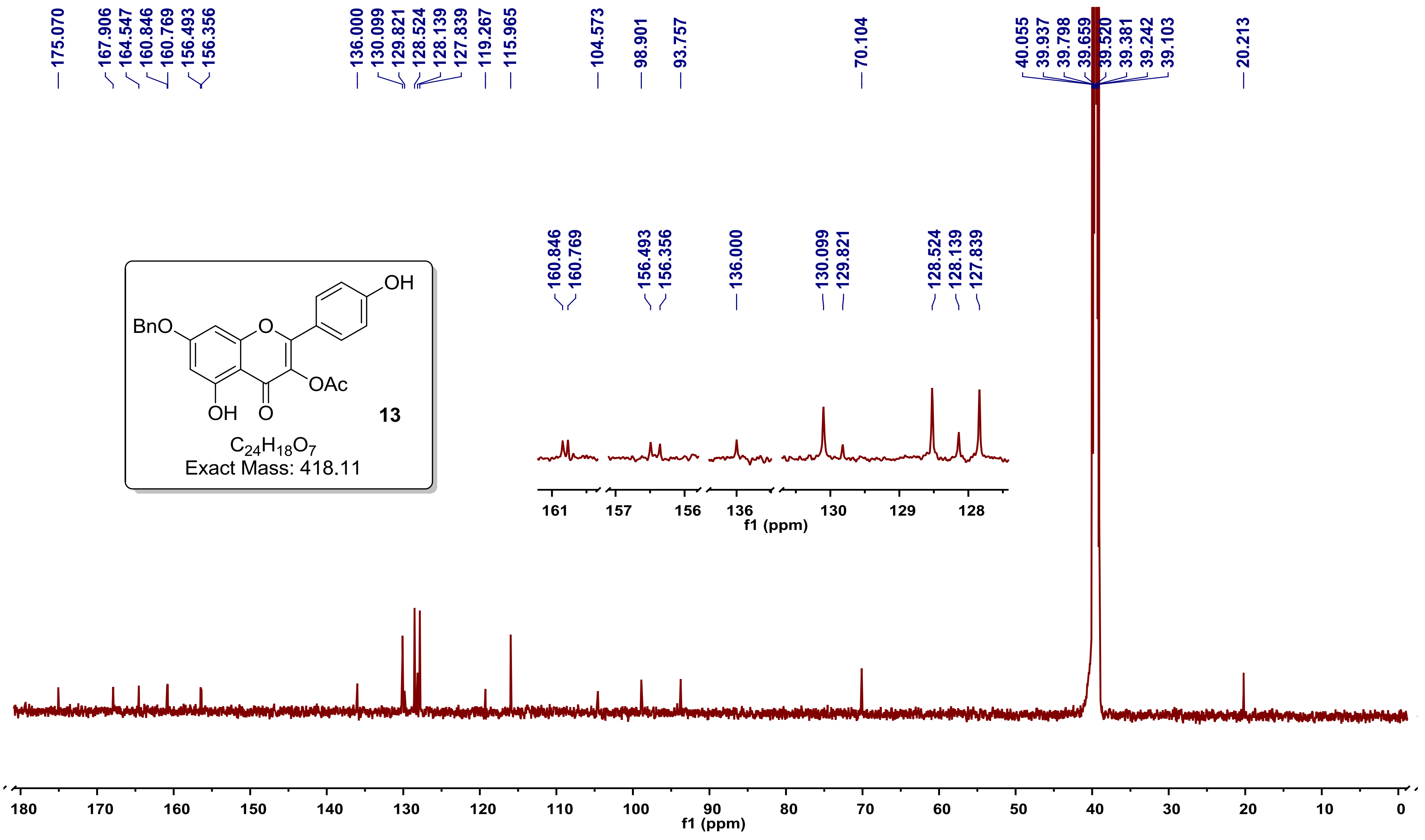

175.070
167.906
164.547
160.846
160.769
156.493
156.356
136.000
130.099
129.821
128.524
128.139
127.839
119.267
115.965
104.573
98.901
93.757
70.104
40.055
39.937
39.798
39.659
39.520
39.381
39.242
39.103
20.213
BnO
O
OH
OAc
OH
O
13
$C_{24}H_{18}O_7$
Exact Mass: 418.11
f1 (ppm)

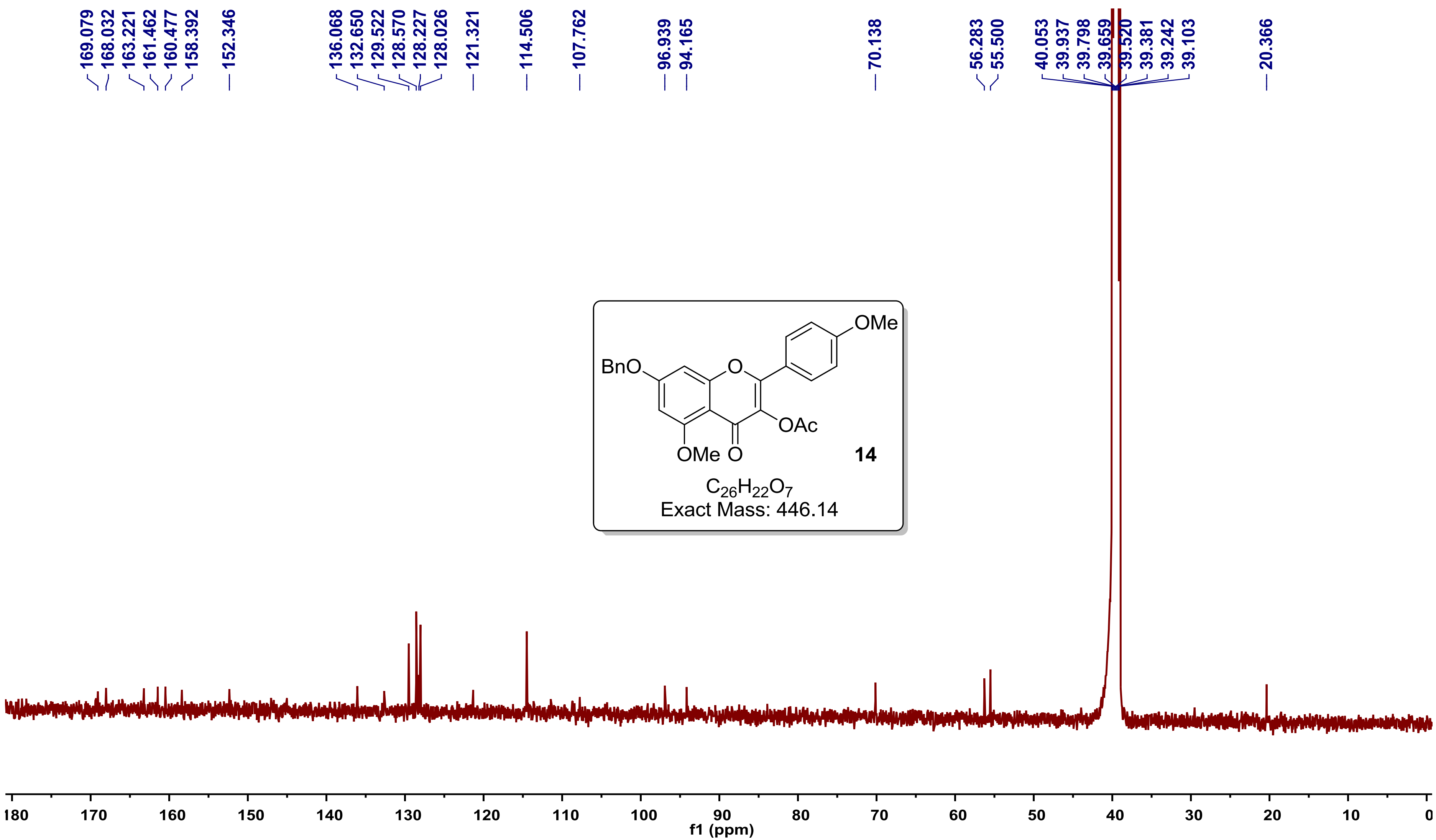

169.079
168.032
163.221
161.462
160.477
158.392
152.346
136.068
132.650
129.522
128.570
128.227
128.026
121.321
114.506
107.762
96.939
94.165
70.138
56.283
55.500
40.053
39.937
39.798
39.659
39.520
39.381
39.242
39.103
20.366
OMe
BnO
O
OAc
OMe O
14
$C_{26}H_{22}O_7$
Exact Mass: 446.14
180
170
160
150
140
130
120
110
100
90
80
70
60
50
40
30
20
10
0
f1 (ppm)

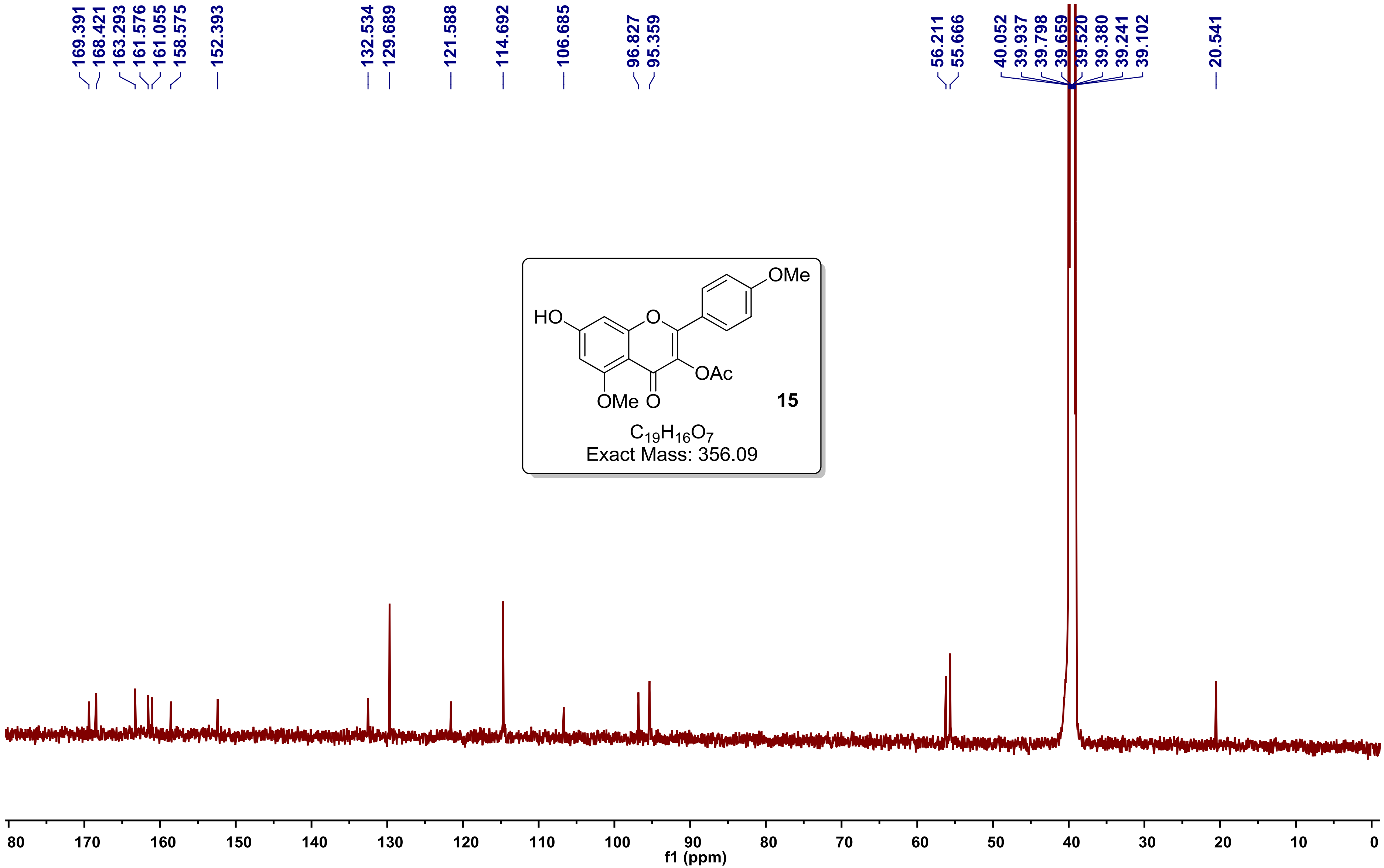
169.391
168.421
163.293
161.576
161.055
158.575
152.393
132.534
129.689
121.588
114.692
106.685
96.827
95.359
56.211
55.666
40.052
39.937
39.798
39.659
39.520
39.380
39.241
39.102
20.541
OMe
HO
O
OAc
OMe O
15
$C_{19}H_{16}O_7$
Exact Mass: 356.09
180 170 160 150 140 130 120 110 100 90 80 70 60 50 40 30 20 10 0
f1 (ppm)

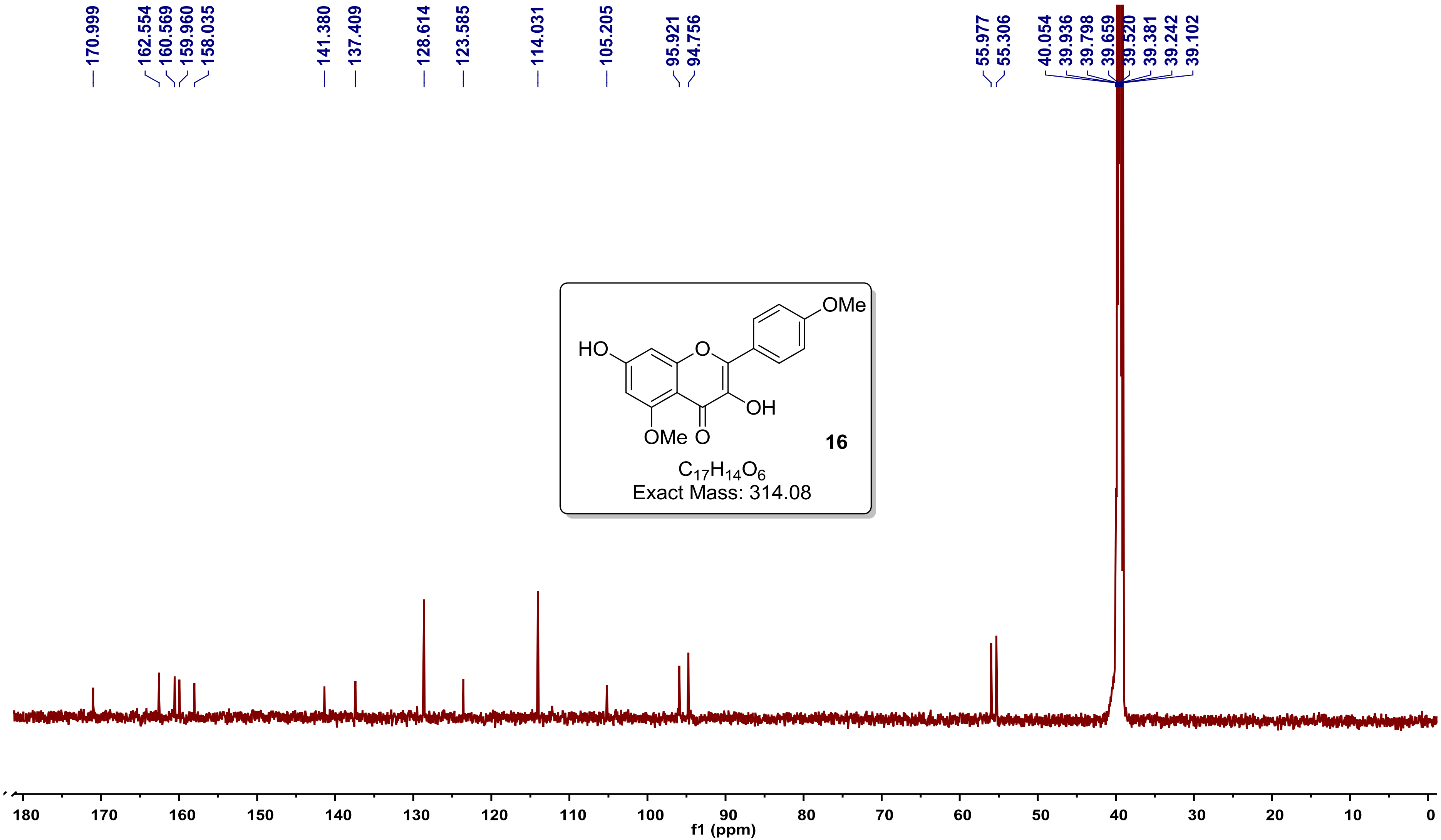
170.999
162.554
160.569
159.960
158.035
141.380
137.409
128.614
123.585
114.031
105.205
95.921
94.756
55.977
55.306
40.054
39.936
39.798
39.659
39.520
39.381
39.242
39.102
OMe
HO
O
OH
OMe O
16
$C_{17}H_{14}O_6$
Exact Mass: 314.08
180 170 160 150 140 130 120 110 100 90 80 70 60 50 40 30 20 10 0
f1 (ppm)

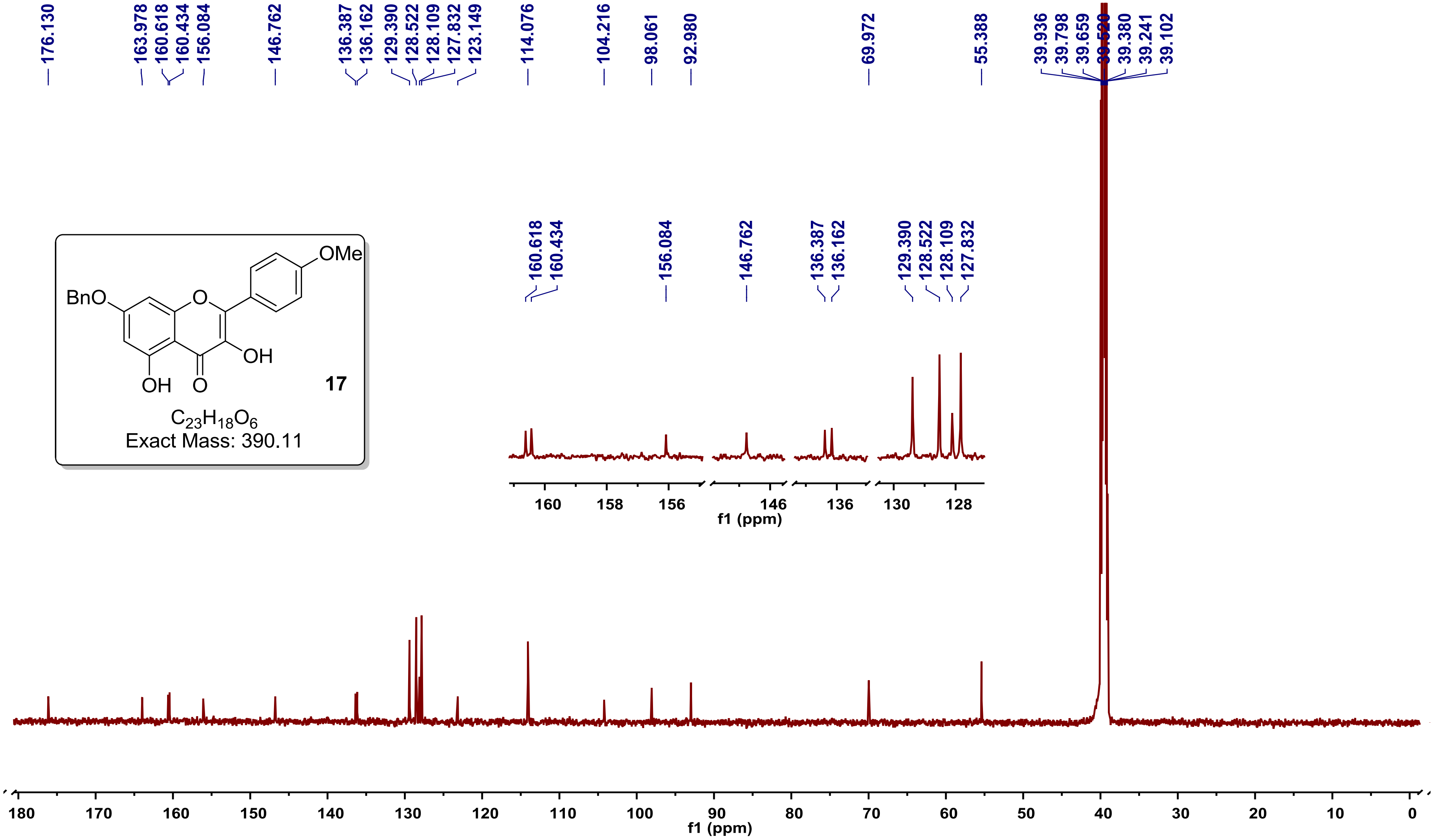

176.130
163.978
160.618
160.434
156.084
146.762
136.387
136.162
129.390
128.522
128.109
127.832
123.149
114.076
104.216
98.061
92.980
69.972
55.388
39.936
39.798
39.659
39.380
39.241
39.102
BnO
O
OMe
OH
OH
O
17
$C_{23}H_{18}O_6$
Exact Mass: 390.11
160.618
160.434
156.084
146.762
136.387
136.162
129.390
128.522
128.109
127.832
160 158 156 146 136 130 128
f1 (ppm)
180 170 160 150 140 130 120 110 100 90 80 70 60 50 40 30 20 10 0
f1 (ppm)

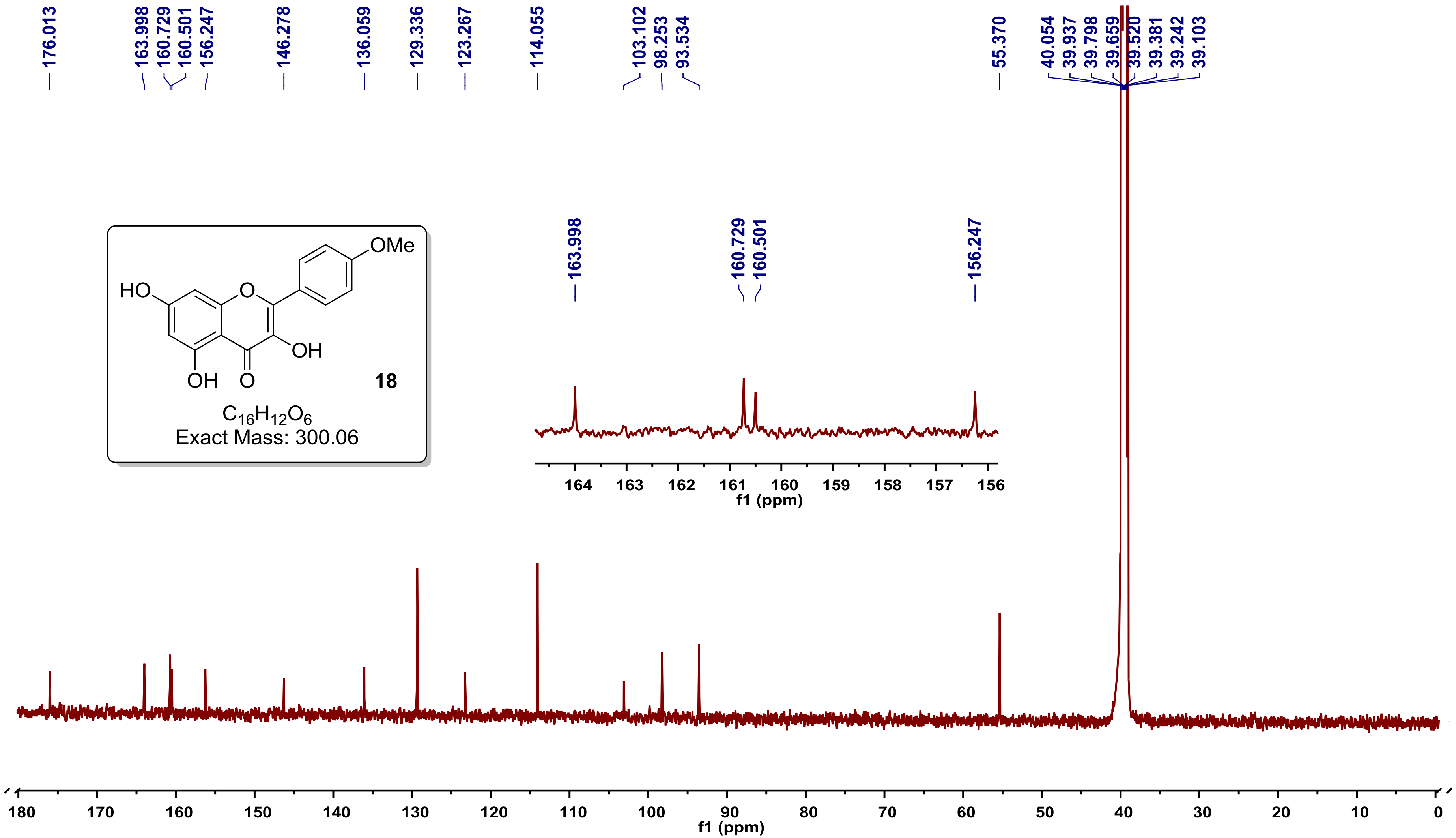

176.013
163.998
160.729
160.501
156.247
146.278
136.059
129.336
123.267
114.055
103.102
98.253
93.534
55.370
40.054
39.937
39.798
39.659
39.520
39.381
39.242
39.103
HO
OMe
OH
OH
O
18
$C_{16}H_{12}O_6$
Exact Mass: 300.06
f1 (ppm)

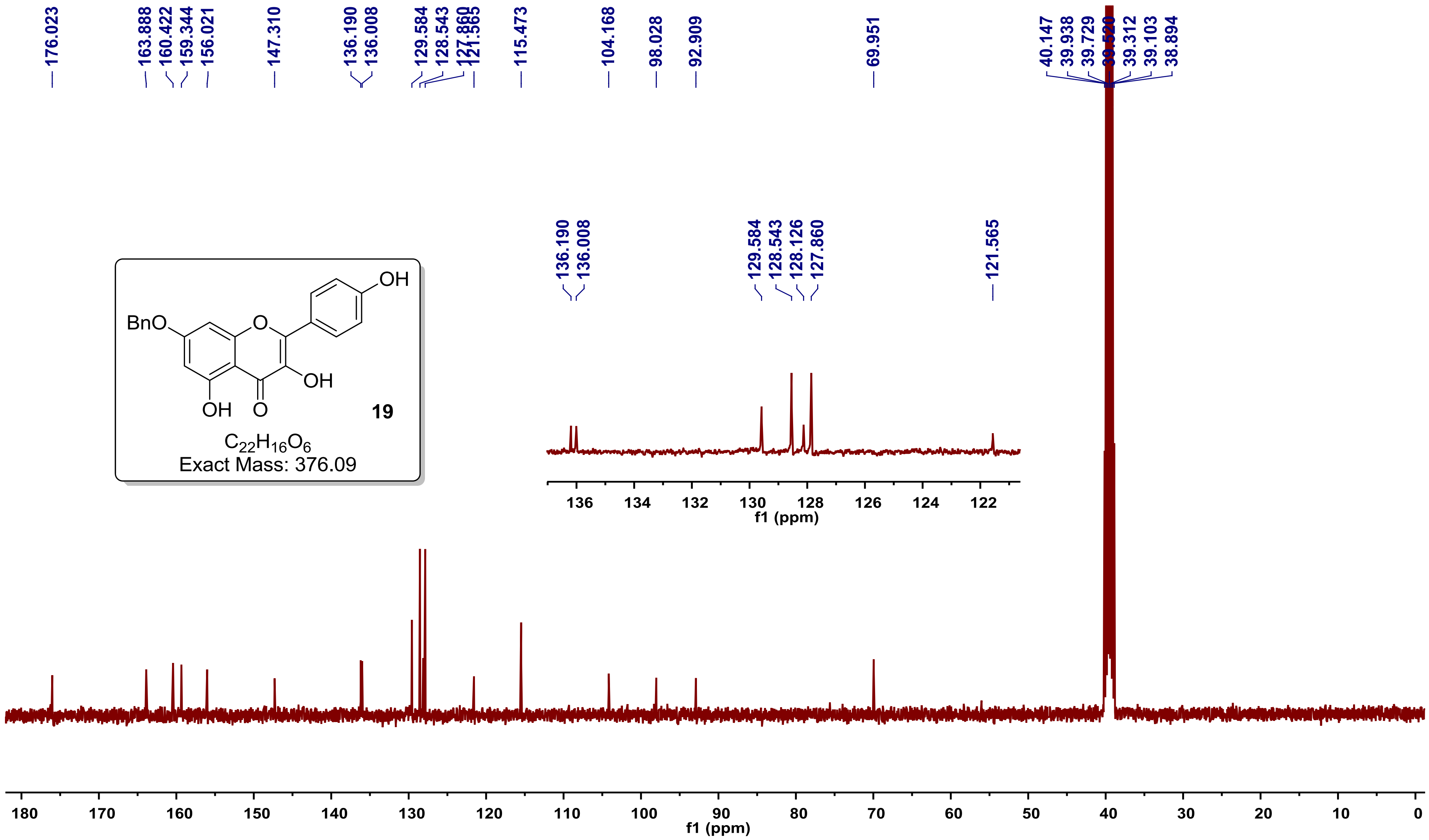

176.023
163.888
160.422
159.344
156.021
147.310
136.190
136.008
129.584
128.543
127.860
121.565
115.473
104.168
98.028
92.909
69.951
40.147
39.938
39.729
39.520
39.312
39.103
38.894
136.190
136.008
129.584
128.543
128.126
127.860
121.565
BnO
O
OH
OH
OH
O
19
$C_{22}H_{16}O_6$
Exact Mass: 376.09
136 134 132 130 128 126 124 122
f1 (ppm)
180 170 160 150 140 130 120 110 100 90 80 70 60 50 40 30 20 10 0
f1 (ppm)

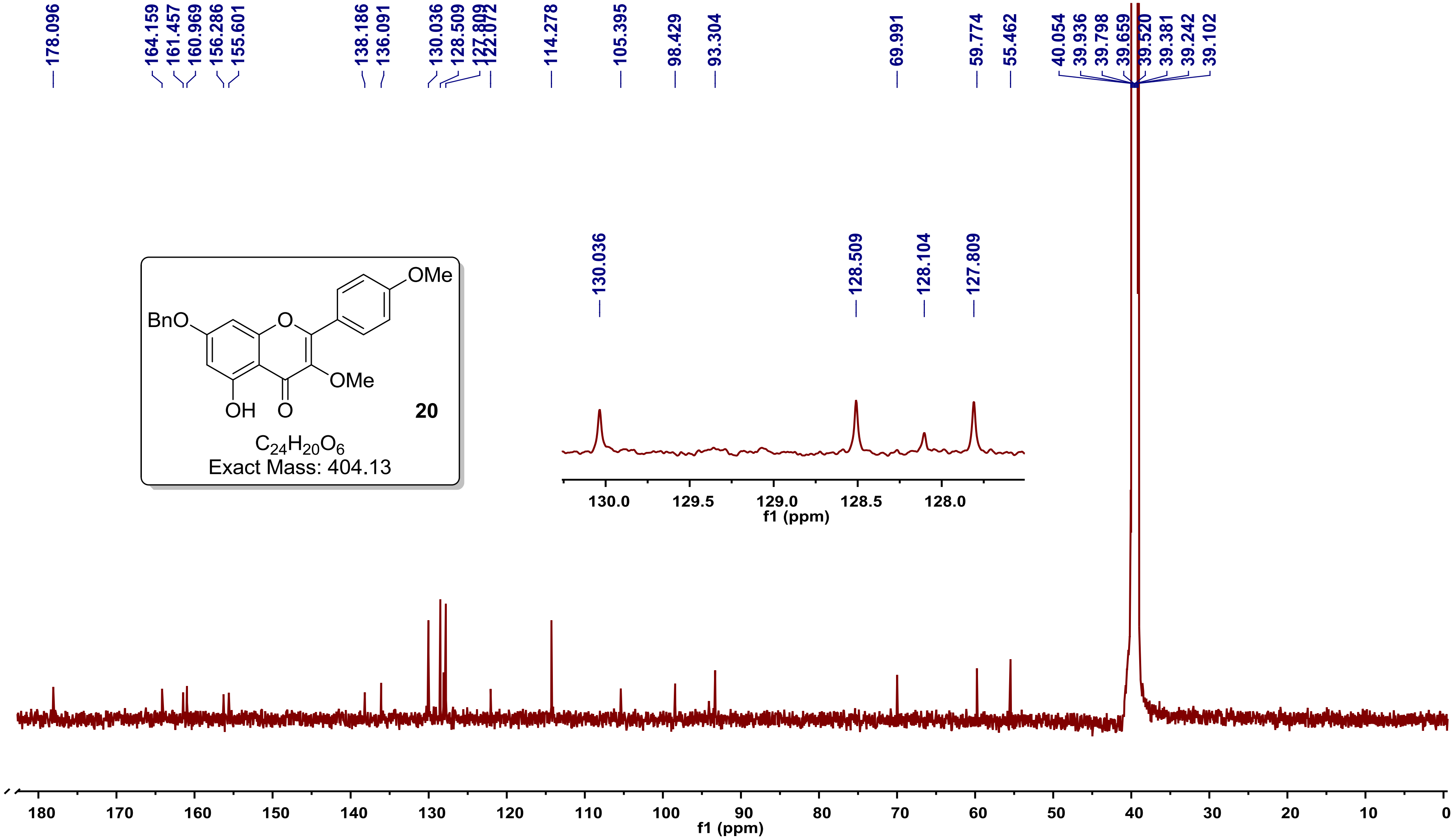
178.096
164.159
161.457
160.969
156.286
155.601
138.186
136.091
130.036
128.509
127.809
122.072
114.278
105.395
98.429
93.304
69.991
59.774
55.462
40.054
39.936
39.798
39.659
39.520
39.381
39.242
39.102
130.036
128.509
128.104
127.809
130.0
129.5
129.0
128.5
128.0
f1 (ppm)
BnO
O
OMe
OMe
OH
O
20
C24H20O6
Exact Mass: 404.13
180
170
160
150
140
130
120
110
100
90
80
70
60
50
40
30
20
10
0
f1 (ppm)

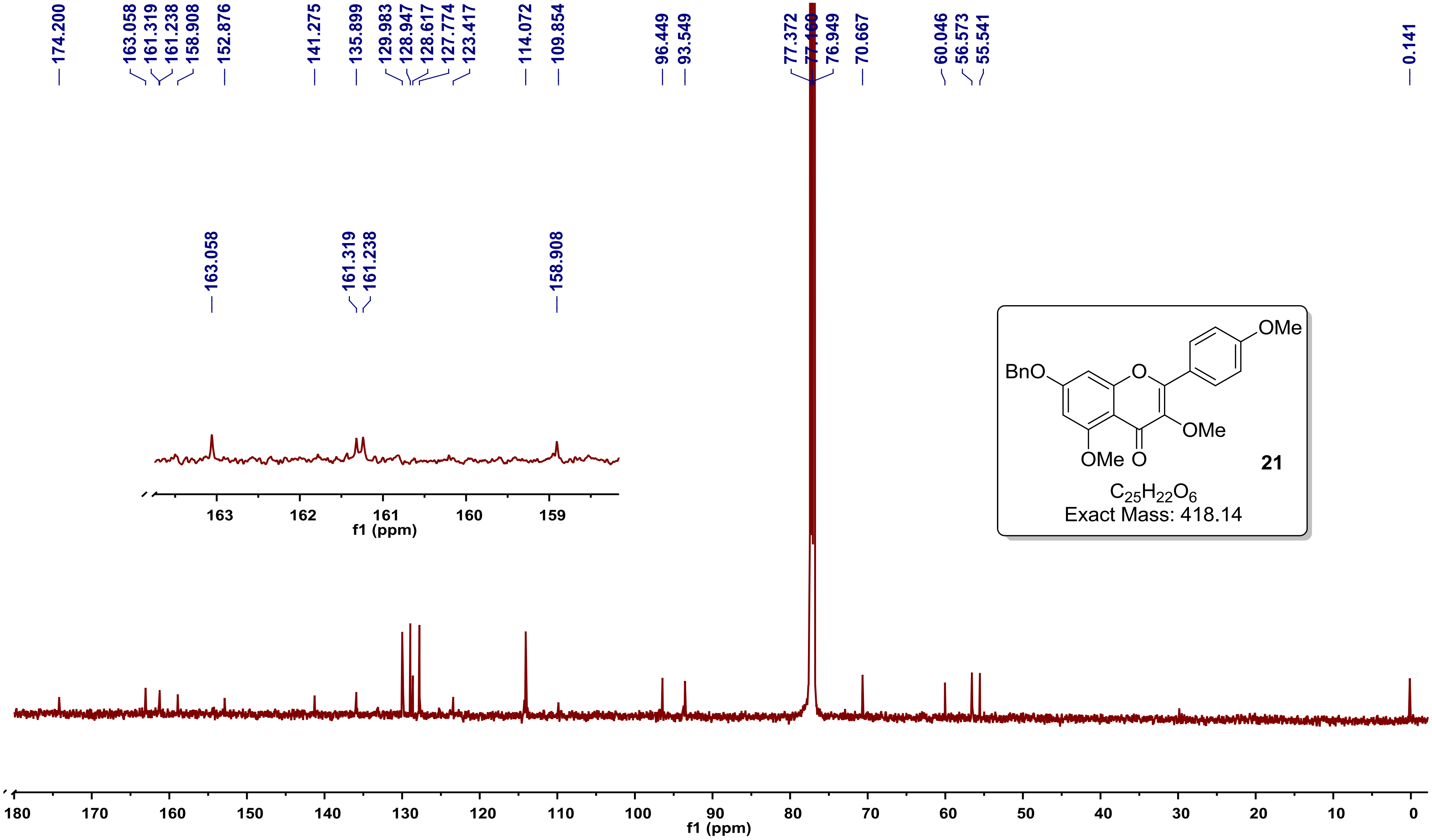
174.200
163.058
161.319
161.238
158.908
152.876
141.275
135.899
129.983
128.947
128.617
127.774
123.417
114.072
109.854
96.449
93.549
77.372
77.160
76.949
70.667
60.046
56.573
55.541
0.141
163.058
161.319
161.238
158.908
163
162
161
160
159
f1 (ppm)
OMe
BnO
O
OMe
OMe O
21
$C_{25}H_{22}O_6$
Exact Mass: 418.14
180
170
160
150
140
130
120
110
100
90
80
70
60
50
40
30
20
10
0
f1 (ppm)

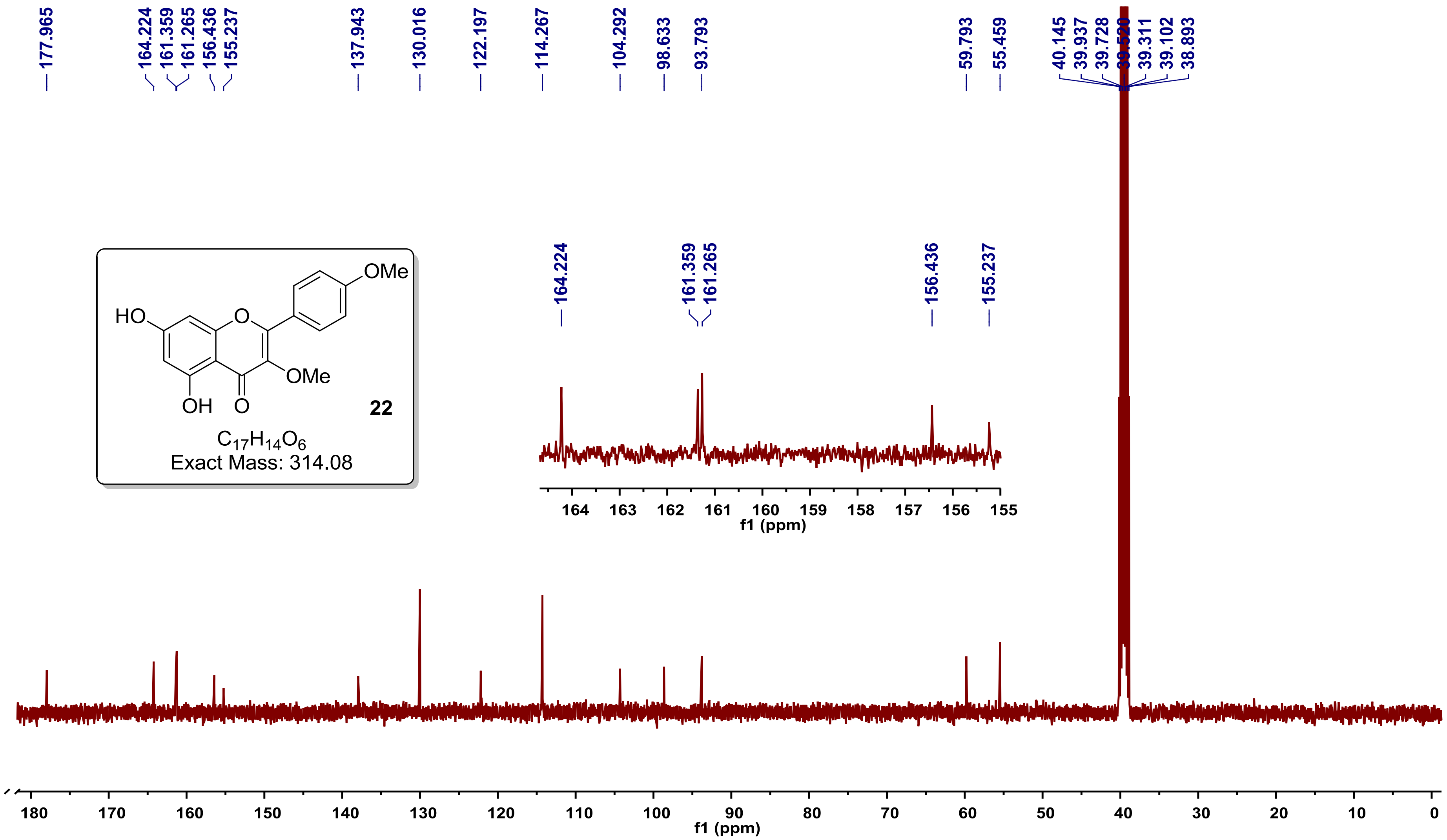
177.965
164.224
161.359
161.265
156.436
155.237
137.943
130.016
122.197
114.267
104.292
98.633
93.793
59.793
55.459
40.145
39.937
39.728
39.311
39.102
38.893
HO
O
OMe
OMe
OH
O
22
$C_{17}H_{14}O_6$
Exact Mass: 314.08
164 163 162 161 160 159 158 157 156 155
f1 (ppm)
180 170 160 150 140 130 120 110 100 90 80 70 60 50 40 30 20 10 0
f1 (ppm)

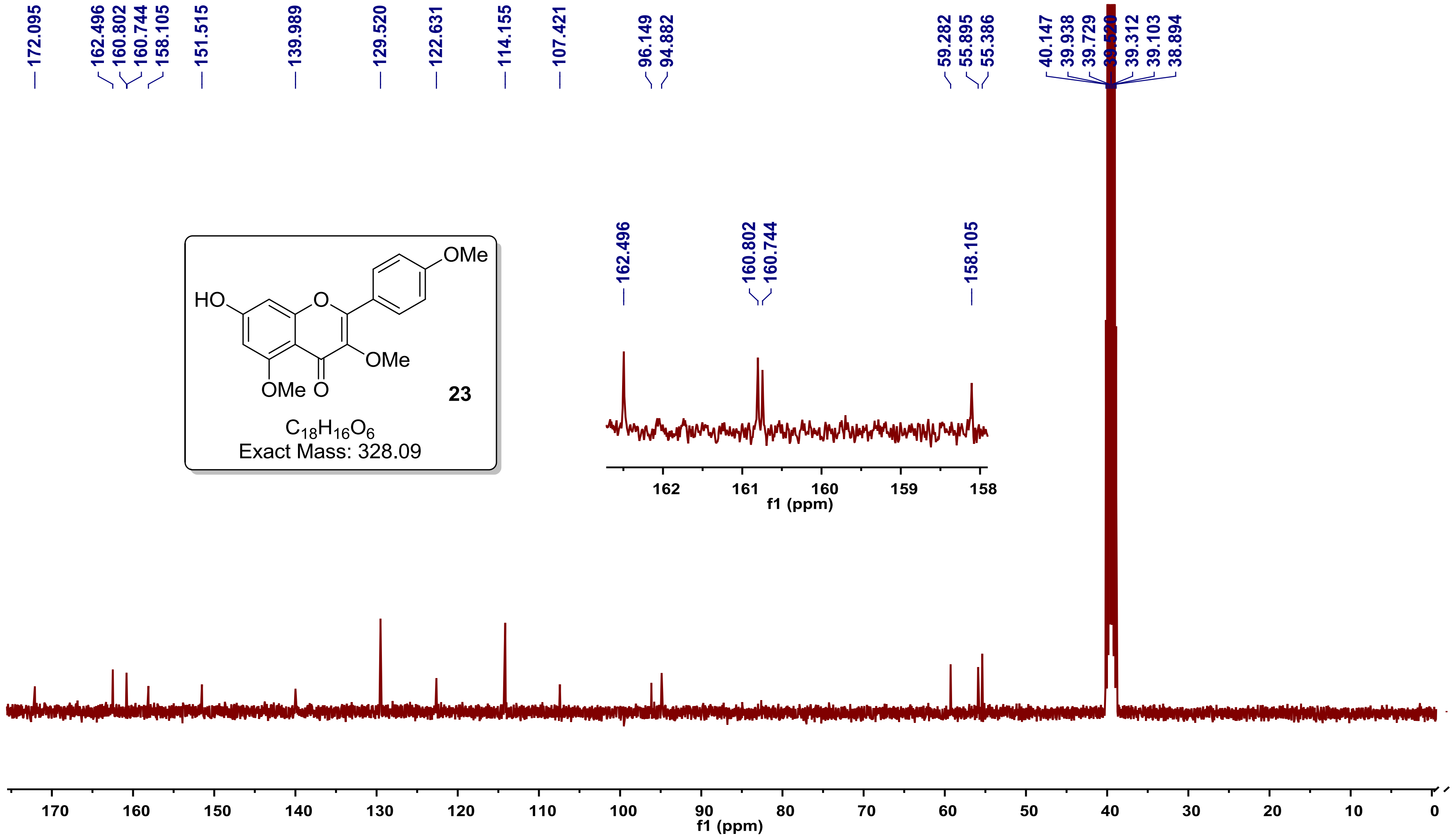
172.095
162.496
160.802
160.744
158.105
151.515
139.989
129.520
122.631
114.155
107.421
96.149
94.882
59.282
55.895
55.386
40.147
39.938
39.729
39.520
39.312
39.103
38.894
OMe
HO
O
OMe
OMe O
23
C18H16O6
Exact Mass: 328.09
162.496
160.802
160.744
158.105
162
161
160
159
158
f1 (ppm)
170
160
150
140
130
120
110
100
90
80
70
60
50
40
30
20
10
0
f1 (ppm)

# Mass Spectrum SmartFormula Report

**Analysis Info**

| | | | |
|---|---|---|---|
| Analysis Name | D:\Data\USER-2013\MEI-454-1.d | Acquisition Date | 1/15/2013 5:07:00 PM |
| Method | WU_tune_low_20121222.m | Operator | Ma |
| Sample Name | MEI-454-1 | Instrument / Ser# | micrOTOF-Q II 10203 |
| Comment | | | |

**Acquisition Parameter**

| | | | | | |
|---|---|---|---|---|---|
| Source Type | ESI | Ion Polarity | Positive | Set Nebulizer | 0.3 Bar |
| Focus | Active | Set Capillary | 4500 V | Set Dry Heater | 180 °C |
| Scan Begin | 50 m/z | Set End Plate Offset | -500 V | Set Dry Gas | 4.0 l/min |
| Scan End | 3000 m/z | Set Collision Cell RF | 150.0 Vpp | Set Divert Valve | Source |

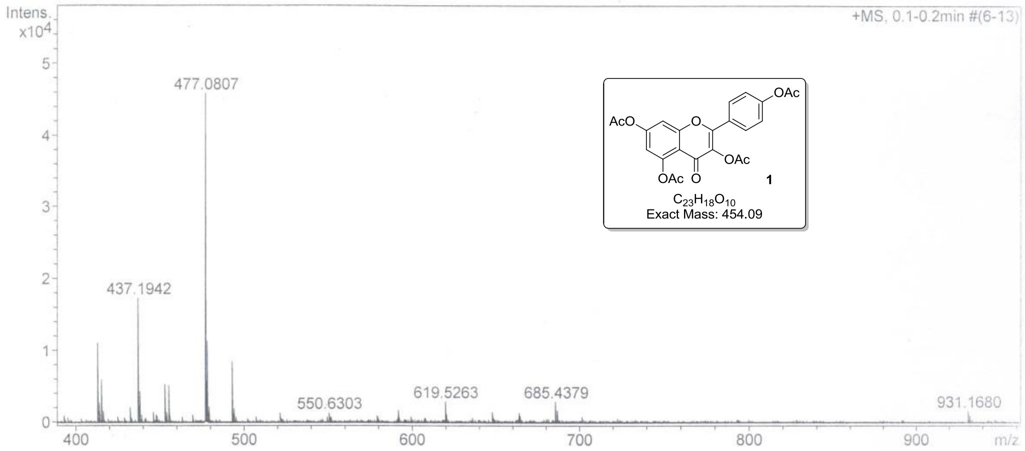


| Meas. m/z | # | Formula | Score | m/z | err [ppm] | Mean err [ppm] | mSigma | rdb | e⁻ Conf | N-Rule |
|---|---|---|---|---|---|---|---|---|---|---|
| 477.0807 | 1 | C 23 H 18 Na O 10 | 100.00 | 477.0792 | -3.2 | -3.1 | 3.5 | 14.5 | even | ok |

Bruker Compass DataAnalysis 4.0 printed: 1/15/2013 5:51:51 PM Page 1 of 1

# Mass Spectrum SmartFormula Report

**Analysis Info**

| | | | |
|---|---|---|---|
| Analysis Name | D:\Data\USER-2014\MEI412+.d | Acquisition Date | 3/19/2014 3:14:49 PM |
| Method | WU_tune_low_20121222.m | Operator | Ma |
| Sample Name | MEI412+ | Instrument / Ser# | micrOTOF-Q II 10203 |
| Comment | | | |

**Acquisition Parameter**

| | | | | | |
|---|---|---|---|---|---|
| Source Type | ESI | Ion Polarity | Positive | Set Nebulizer | 0.4 Bar |
| Focus | Not active | Set Capillary | 4500 V | Set Dry Heater | 180 °C |
| Scan Begin | 50 m/z | Set End Plate Offset | -500 V | Set Dry Gas | 3.0 l/min |
| Scan End | 3000 m/z | Set Collision Cell RF | 150.0 Vpp | Set Divert Valve | Source |

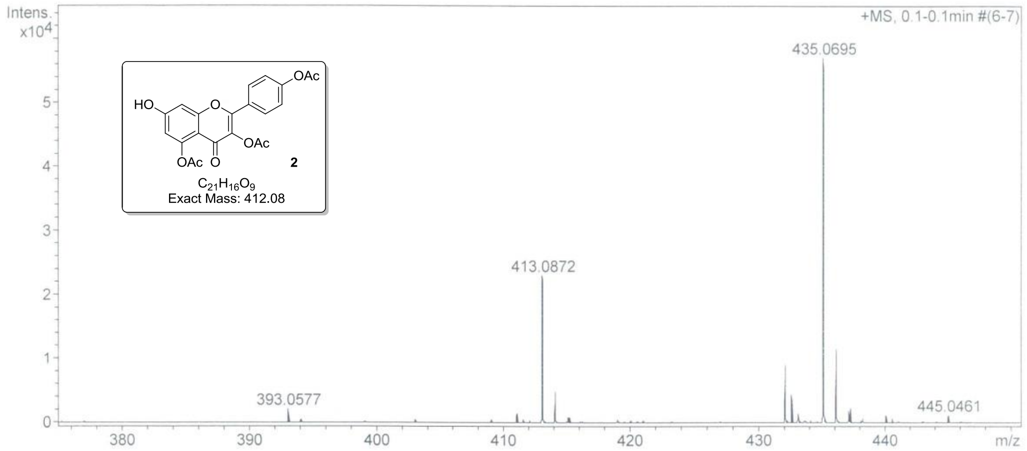


| Meas. m/z | # | Formula | Score | m/z | err [ppm] | Mean err [ppm] | mSigma | rdb | e⁻ Conf | N-Rule |
|---|---|---|---|---|---|---|---|---|---|---|
| 413.0872 | 1 | C 21 H 17 O 9 | 100.00 | 413.0867 | -1.3 | -0.6 | 9.5 | 13.5 | even | ok |
| 413.0884 | 1 | C 22 H 13 N 4 O 5 | | 413.0880 | -0.9 | | n.a. | 18.5 | even | ok |
| 435.0695 | 1 | C 21 H 16 Na O 9 | 100.00 | 435.0687 | -2.0 | -1.3 | 14.9 | 13.5 | even | ok |

# Mass Spectrum SmartFormula Report

**Analysis Info**

| | | | |
|---|---|---|---|
| Analysis Name | D:\Data\USER-2014\mei83008.d | Acquisition Date | 9/18/2014 10:25:37 AM |
| Method | WU_tune_low_20121222.m | Operator | Ma |
| Sample Name | mei83008 | Instrument / Ser# | micrOTOF-Q II 10203 |
| Comment | | | |

**Acquisition Parameter**

| | | | | | |
|---|---|---|---|---|---|
| Source Type | ESI | Ion Polarity | Positive | Set Nebulizer | 0.4 Bar |
| Focus | Not active | Set Capillary | 4500 V | Set Dry Heater | 180 °C |
| Scan Begin | 30 m/z | Set End Plate Offset | -500 V | Set Dry Gas | 3.0 l/min |
| Scan End | 3000 m/z | Set Collision Cell RF | 150.0 Vpp | Set Divert Valve | Source |

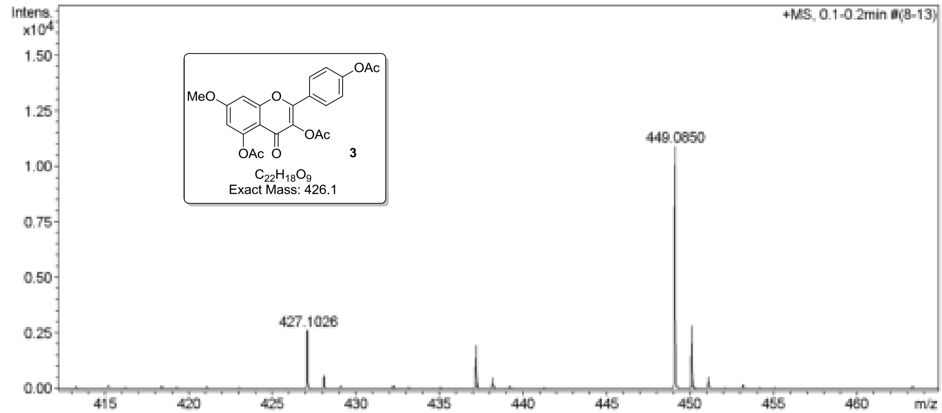


| Meas. m/z | # | Formula | Score | m/z | err [ppm] | Mean err [ppm] | mSigma | rdb | e⁻ Conf | N-Rule |
|---|---|---|---|---|---|---|---|---|---|---|
| 427.1026 | 1 | C 22 H 19 O 9 | 100.00 | 427.1024 | -0.5 | -1.0 | 5.8 | 13.5 | even | ok |
| 449.0850 | 1 | C 22 H 18 Na O 9 | 100.00 | 449.0843 | -1.6 | -1.3 | 11.0 | 13.5 | even | ok |

# Mass Spectrum SmartFormula Report

**Analysis Info**

| | | | |
|---|---|---|---|
| Analysis Name | D:\Data\USER-2014\0619.d | Acquisition Date | 9/27/2014 1:02:53 PM |
| Method | WU_tune_low_20121222.m | Operator | Ma |
| Sample Name | 0619 | Instrument / Ser# | micrOTOF-Q II 10203 |
| Comment | | | |

**Acquisition Parameter**

| | | | | | |
|---|---|---|---|---|---|
| Source Type | ESI | Ion Polarity | Negative | Set Nebulizer | 0.4 Bar |
| Focus | Not active | Set Capillary | 4000 V | Set Dry Heater | 180 °C |
| Scan Begin | 30 m/z | Set End Plate Offset | -500 V | Set Dry Gas | 3.0 l/min |
| Scan End | 3000 m/z | Set Collision Cell RF | 150.0 Vpp | Set Divert Valve | Source |

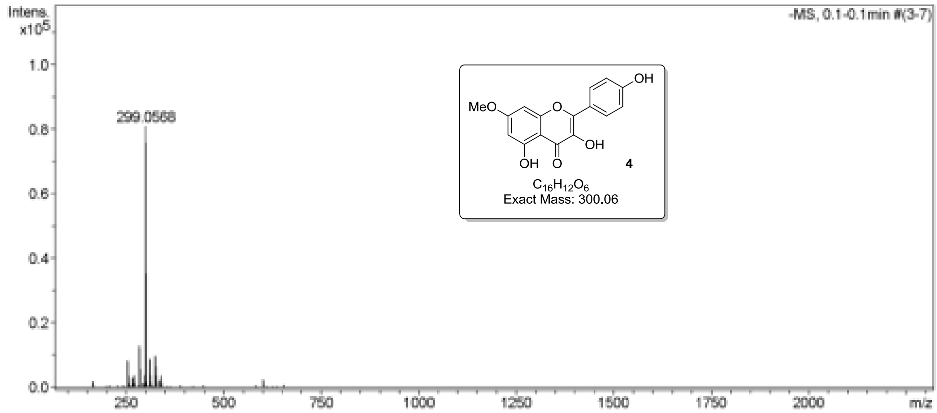


| Meas. m/z | # | Formula | Score | m/z | err [ppm] | Mean err [ppm] | mSigma | rdb | e⁻ Conf | N-Rule |
|---|---|---|---|---|---|---|---|---|---|---|
| 299.0568 | 1 | C 16 H 11 O 6 | 100.00 | 299.0561 | -2.3 | -2.0 | 3.4 | 11.5 | even | ok |

Bruker Compass DataAnalysis 4.0 printed: 9/27/2014 1:17:49 PM Page 1 of 1

# Mass Spectrum SmartFormula Report

**Analysis Info**

| | | | |
|---|---|---|---|
| Analysis Name | D:\Data\USER-2014\MEI314-2.d | Acquisition Date | 3/26/2014 2:54:50 PM |
| Method | WU_tune_low_20121222.m | Operator | Ma |
| Sample Name | MEI314-2 | Instrument / Ser# | micrOTOF-Q II 10203 |
| Comment | | | |

**Acquisition Parameter**

| | | | | | |
|---|---|---|---|---|---|
| Source Type | ESI | Ion Polarity | Positive | Set Nebulizer | 0.4 Bar |
| Focus | Not active | Set Capillary | 4500 V | Set Dry Heater | 180 °C |
| Scan Begin | 50 m/z | Set End Plate Offset | -500 V | Set Dry Gas | 3.0 l/min |
| Scan End | 3000 m/z | Set Collision Cell RF | 150.0 Vpp | Set Divert Valve | Source |

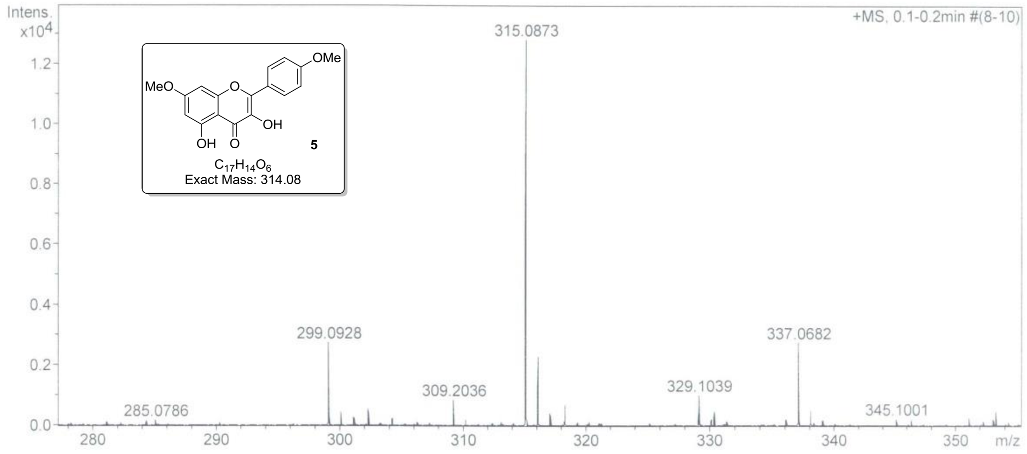


| Meas. m/z | # | Formula | Score | m/z | err [ppm] | Mean err [ppm] | mSigma | rdb | e⁻ Conf | N-Rule |
|---|---|---|---|---|---|---|---|---|---|---|
| 315.0873 | 1 | C 17 H 15 O 6 | 100.00 | 315.0863 | -3.0 | -2.9 | 3.4 | 10.5 | even | ok |
| 337.0682 | 1 | C 17 H 14 Na O 6 | 100.00 | 337.0683 | 0.1 | 2.9 | 18.4 | 10.5 | even | ok |

Bruker Compass DataAnalysis 4.0 printed: 3/26/2014 2:57:26 PM Page 1 of 1

# Mass Spectrum SmartFormula Report

**Analysis Info**

| | | | |
|---|---|---|---|
| Analysis Name | D:\Data\USER-2014\mei328.d | Acquisition Date | 4/23/2014 4:51:34 PM |
| Method | WU_tune_low_20121222.m | Operator | Ma |
| Sample Name | mei328 | Instrument / Ser# | micrOTOF-Q II 10203 |
| Comment | | | |

**Acquisition Parameter**

| | | | | | |
|---|---|---|---|---|---|
| Source Type | ESI | Ion Polarity | Negative | Set Nebulizer | 0.4 Bar |
| Focus | Not active | Set Capillary | 4000 V | Set Dry Heater | 180 °C |
| Scan Begin | 50 m/z | Set End Plate Offset | -500 V | Set Dry Gas | 3.0 l/min |
| Scan End | 3000 m/z | Set Collision Cell RF | 150.0 Vpp | Set Divert Valve | Source |

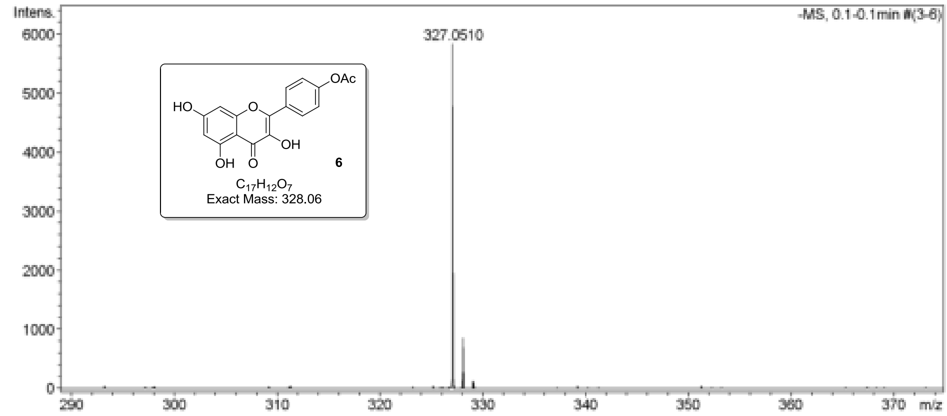


| Meas. m/z | # | Formula | Score | m/z | err [ppm] | Mean err [ppm] | mSigma | rdb | e⁻ Conf | N-Rule |
|---|---|---|---|---|---|---|---|---|---|---|
| 327.0510 | 1 | C 17 H 11 O 7 | 100.00 | 327.0510 | 0.1 | 0.1 | 21.8 | 12.5 | even | ok |

# Mass Spectrum SmartFormula Report

**Analysis Info**

| | | | |
|---|---|---|---|
| Analysis Name | D:\Data\USER-2014\mei0606.d | Acquisition Date | 9/17/2014 2:53:23 PM |
| Method | WU_tune_low_20121222.m | Operator | Ma |
| Sample Name | mei0606 | Instrument / Ser# | micrOTOF-Q II 10203 |
| Comment | | | |

**Acquisition Parameter**

| | | | | | | | |
|---|---|---|---|---|---|---|---|
| Source Type | ESI | Ion Polarity | Positive | Set Nebulizer | 0.4 Bar | | |
| Focus | Not active | Set Capillary | 4500 V | Set Dry Heater | 180 °C | | |
| Scan Begin | 30 m/z | Set End Plate Offset | -500 V | Set Dry Gas | 3.0 l/min | | |
| Scan End | 3000 m/z | Set Collision Cell RF | 150.0 Vpp | Set Divert Valve | Source | | |

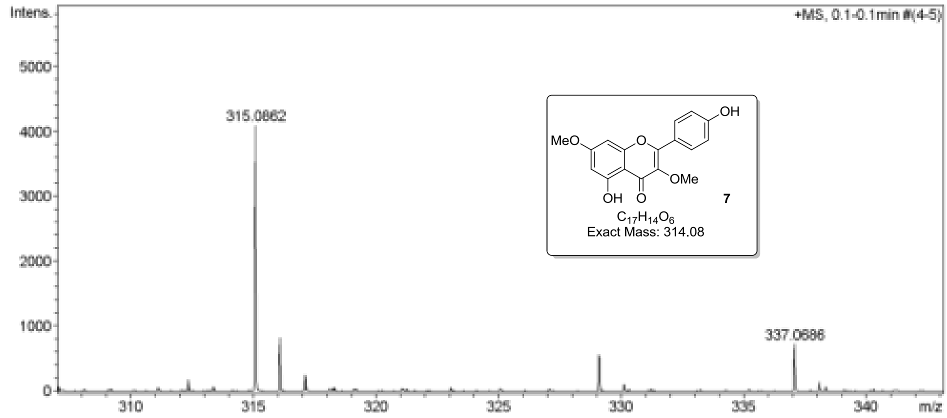


| Meas. m/z | # | Formula | Score | m/z | err [ppm] | Mean err [ppm] | mSigma | rdb | e⁻ Conf | N-Rule |
|---|---|---|---|---|---|---|---|---|---|---|
| 315.0862 | 1 | C 17 H 15 O 6 | 100.00 | 315.0863 | 0.4 | -1.2 | 20.7 | 10.5 | even | ok |
| 337.0686 | 1 | C 17 H 14 Na O 6 | 100.00 | 337.0683 | -1.1 | -2.6 | 16.4 | 10.5 | even | ok |

# Mass Spectrum SmartFormula Report

**Analysis Info**

| | | | |
|---|---|---|---|
| Analysis Name | D:\Data\USER-2013\M502.d | Acquisition Date | 3/11/2013 4:35:28 PM |
| Method | WU_tune_low_20121222.m | Operator | Ma |
| Sample Name | M502 | Instrument / Ser# | micrOTOF-Q II 10203 |
| Comment | | | |

**Acquisition Parameter**

| | | | | | |
|---|---|---|---|---|---|
| Source Type | ESI | Ion Polarity | Positive | Set Nebulizer | 0.3 Bar |
| Focus | Not active | Set Capillary | 4500 V | Set Dry Heater | 180 °C |
| Scan Begin | 50 m/z | Set End Plate Offset | -500 V | Set Dry Gas | 4.0 l/min |
| Scan End | 3000 m/z | Set Collision Cell RF | 150.0 Vpp | Set Divert Valve | Source |

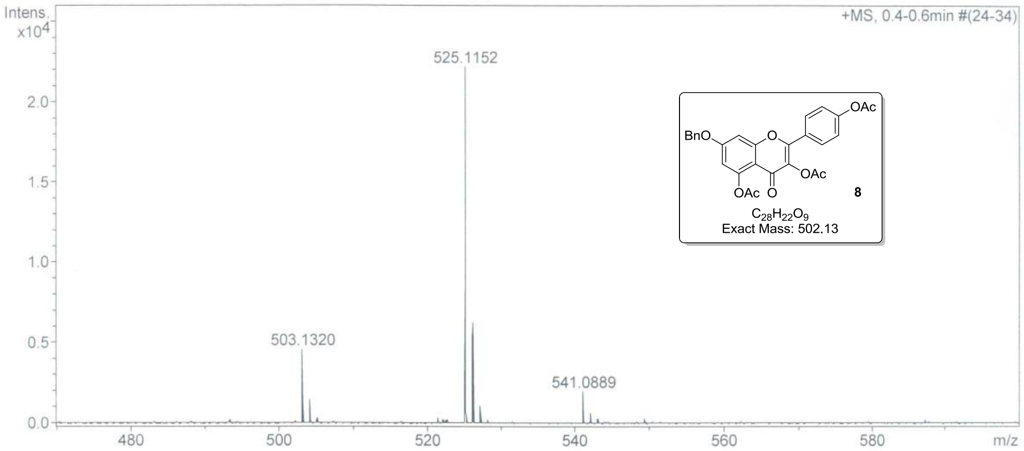


| Meas. m/z | # | Formula | Score | m/z | err [ppm] | Mean err [ppm] | mSigma | rdb | e⁻ Conf | N-Rule |
|---|---|---|---|---|---|---|---|---|---|---|
| 503.1320 | 1 | C 28 H 23 O 9 | 51.57 | 503.1337 | 3.3 | 4.8 | 12.4 | 17.5 | even | ok |
| | 2 | C 26 H 21 N 3 O 8 | 100.00 | 503.1323 | 0.6 | 2.1 | 17.3 | 18.0 | odd | ok |
| | 3 | C 23 H 23 N 2 O 11 | 18.55 | 503.1296 | -4.7 | -3.2 | 33.1 | 13.5 | even | ok |
| | 4 | C 38 H 17 N O | 24.51 | 503.1305 | -3.1 | -1.5 | 45.9 | 31.0 | odd | ok |
| 525.1152 | 1 | C 26 H 20 N 3 Na O 8 | 84.25 | 525.1143 | -1.8 | -1.4 | 6.7 | 18.0 | odd | ok |
| | 2 | C 28 H 22 Na O 9 | 100.00 | 525.1156 | 0.8 | 1.2 | 12.3 | 17.5 | even | ok |
| | 3 | C 29 H 18 N 4 Na O 5 | 35.57 | 525.1169 | 3.3 | 3.7 | 24.3 | 22.5 | even | ok |

# Mass Spectrum SmartFormula Report

**Analysis Info**

| | | | |
|---|---|---|---|
| Analysis Name | D:\Data\USER-2014\MEITBTS.d | Acquisition Date | 4/10/2014 3:25:02 PM |
| Method | WU_tune_low_20121222.m | Operator | Ma |
| Sample Name | MEITBTS | Instrument / Ser# | micrOTOF-Q II 10203 |
| Comment | | | |

**Acquisition Parameter**

| | | | | | |
|---|---|---|---|---|---|
| Source Type | ESI | Ion Polarity | Positive | Set Nebulizer | 0.3 Bar |
| Focus | Not active | Set Capillary | 4500 V | Set Dry Heater | 180 °C |
| Scan Begin | 50 m/z | Set End Plate Offset | -500 V | Set Dry Gas | 3.0 l/min |
| Scan End | 3000 m/z | Set Collision Cell RF | 150.0 Vpp | Set Divert Valve | Source |

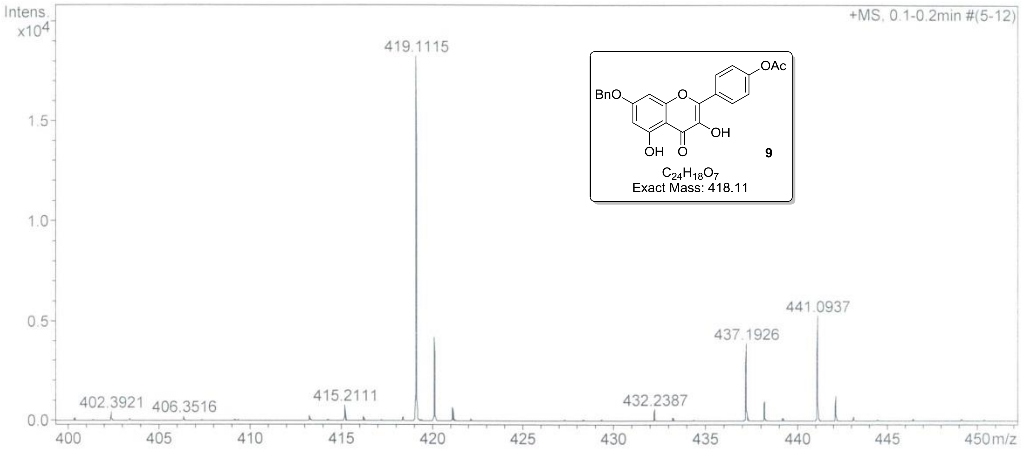


| Meas. m/z | # | Formula | Score | m/z | err [ppm] | Mean err [ppm] | mSigma | rdb | e⁻ Conf | N-Rule |
|---|---|---|---|---|---|---|---|---|---|---|
| 419.1115 | 1 | C 24 H 19 O 7 | 100.00 | 419.1125 | 2.5 | 2.3 | 14.4 | 15.5 | even | ok |

# Mass Spectrum SmartFormula Report

**Analysis Info**

Analysis Name D:\Data\USER-2014\mei0716.d
Method WU_tune_low_20121222.m
Sample Name mei0716
Comment

Acquisition Date 9/18/2014 10:02:01 AM
Operator Ma
Instrument / Ser# micrOTOF-Q II 10203

**Acquisition Parameter**

| | | | | | |
|---|---|---|---|---|---|
| Source Type | ESI | Ion Polarity | Positive | Set Nebulizer | 0.4 Bar |
| Focus | Not active | Set Capillary | 4500 V | Set Dry Heater | 180 °C |
| Scan Begin | 30 m/z | Set End Plate Offset | -500 V | Set Dry Gas | 3.0 l/min |
| Scan End | 3000 m/z | Set Collision Cell RF | 150.0 Vpp | Set Divert Valve | Source |

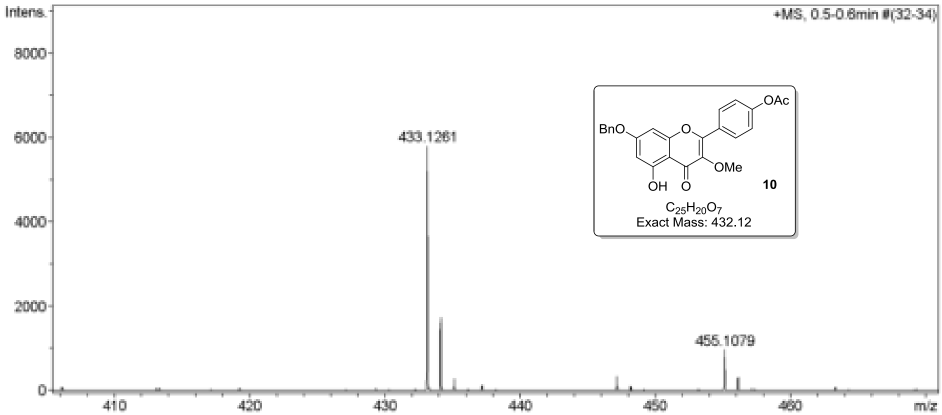


| Meas. m/z | # | Formula | Score | m/z | err [ppm] | Mean err [ppm] | mSigma | rdb | e⁻ Conf | N-Rule |
|---|---|---|---|---|---|---|---|---|---|---|
| 433.1261 | 1 | C 25 H 21 O 7 | 100.00 | 433.1282 | 4.7 | 4.9 | 13.0 | 15.5 | even | ok |
| 455.1079 | 1 | C 25 H 20 Na O 7 | 100.00 | 455.1101 | 5.0 | 5.2 | 38.6 | 15.5 | even | ok |

Bruker Compass DataAnalysis 4.0 printed: 9/23/2014 9:17:17 AM Page 1 of 1

# Mass Spectrum SmartFormula Report

**Analysis Info**

| | | | |
|---|---|---|---|
| Analysis Name | D:\Data\USER-2014\mei37610.d | Acquisition Date | 4/23/2014 4:48:20 PM |
| Method | WU_tune_low_20121222.m | Operator | Ma |
| Sample Name | mei37610 | Instrument / Ser# | micrOTOF-Q II 10203 |
| Comment | | | |

**Acquisition Parameter**

| | | | | | | | |
|---|---|---|---|---|---|---|---|
| Source Type | ESI | Ion Polarity | Negative | Set Nebulizer | 0.4 Bar | | |
| Focus | Not active | Set Capillary | 4000 V | Set Dry Heater | 180 °C | | |
| Scan Begin | 50 m/z | Set End Plate Offset | -500 V | Set Dry Gas | 3.0 l/min | | |
| Scan End | 3000 m/z | Set Collision Cell RF | 150.0 Vpp | Set Divert Valve | Source | | |

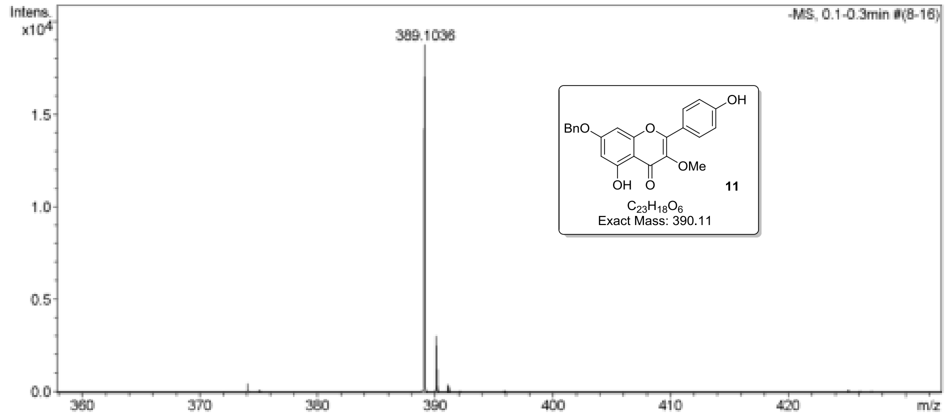


| Meas. m/z | # | Formula | Score | m/z | err [ppm] | Mean err [ppm] | mSigma | rdb | e⁻ Conf | N-Rule |
|---|---|---|---|---|---|---|---|---|---|---|
| 389.1036 | 1 | C 23 H 17 O 6 | 100.00 | 389.1031 | -1.4 | -1.6 | 52.1 | 15.5 | even | ok |

## Mass Spectrum SmartFormula Report

**Analysis Info**

| | | | |
|---|---|---|---|
| Analysis Name | D:\Data\USER-2014\3300.d | Acquisition Date | 9/27/2014 1:04:19 PM |
| Method | WU_tune_low_20121222.m | Operator | Ma |
| Sample Name | 3300 | Instrument / Ser# | micrOTOF-Q II 10203 |
| Comment | | | |

**Acquisition Parameter**

| | | | | | |
|---|---|---|---|---|---|
| Source Type | ESI | Ion Polarity | Negative | Set Nebulizer | 0.4 Bar |
| Focus | Not active | Set Capillary | 4000 V | Set Dry Heater | 180 °C |
| Scan Begin | 30 m/z | Set End Plate Offset | -500 V | Set Dry Gas | 3.0 l/min |
| Scan End | 3000 m/z | Set Collision Cell RF | 150.0 Vpp | Set Divert Valve | Source |

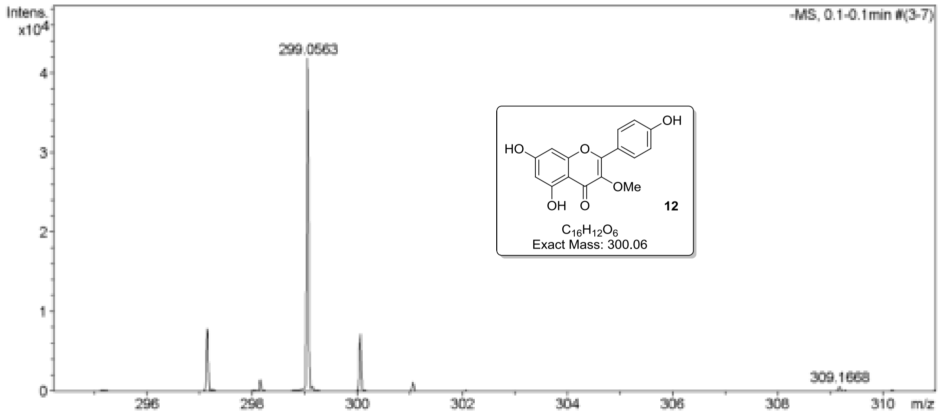


| Meas. m/z | # | Formula | Score | m/z | err [ppm] | Mean err [ppm] | mSigma | rdb | e⁻ Conf | N-Rule |
|---|---|---|---|---|---|---|---|---|---|---|
| 299.0563 | 1 | C 16 H 11 O 6 | 100.00 | 299.0561 | -0.5 | -0.1 | 0.8 | 11.5 | even | ok |

# Mass Spectrum SmartFormula Report

**Analysis Info**

| | | | |
|---|---|---|---|
| Analysis Name | D:\Data\USER-2013\M418.d | Acquisition Date | 3/28/2013 4:28:26 PM |
| Method | WU_tune_low_20121222.m | Operator | Ma |
| Sample Name | M418 | Instrument / Ser# | micrOTOF-Q II 10203 |
| Comment | | | |

**Acquisition Parameter**

| | | | | | |
|---|---|---|---|---|---|
| Source Type | ESI | Ion Polarity | Negative | Set Nebulizer | 0.3 Bar |
| Focus | Not active | Set Capillary | 4000 V | Set Dry Heater | 180 °C |
| Scan Begin | 50 m/z | Set End Plate Offset | -500 V | Set Dry Gas | 4.0 l/min |
| Scan End | 3000 m/z | Set Collision Cell RF | 150.0 Vpp | Set Divert Valve | Source |

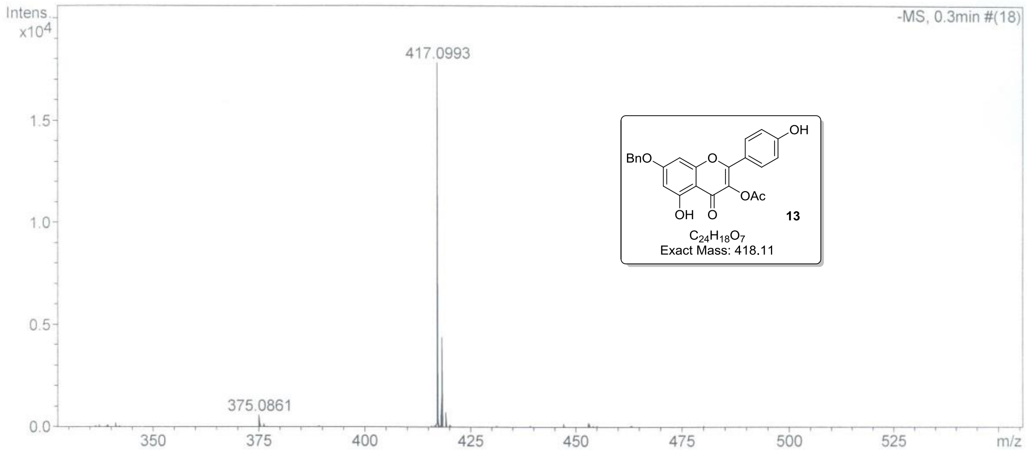


| Meas. m/z | # | Formula | Score | m/z | err [ppm] | Mean err [ppm] | mSigma | rdb | e⁻ Conf | N-Rule |
|---|---|---|---|---|---|---|---|---|---|---|
| 417.0993 | 1 | C 24 H 17 O 7 | 100.00 | 417.0980 | -3.2 | -3.0 | 7.9 | 16.5 | even | ok |

# Mass Spectrum SmartFormula Report

**Analysis Info**

| | | | |
|---|---|---|---|
| Analysis Name | D:\Data\USER-2014\mei51316.d | Acquisition Date | 9/18/2014 10:17:32 AM |
| Method | WU_tune_low_20121222.m | Operator | Ma |
| Sample Name | mei51316 | Instrument / Ser# | micrOTOF-Q II 10203 |
| Comment | | | |

**Acquisition Parameter**

| | | | | | |
|---|---|---|---|---|---|
| Source Type | ESI | Ion Polarity | Positive | Set Nebulizer | 0.4 Bar |
| Focus | Not active | Set Capillary | 4500 V | Set Dry Heater | 180 °C |
| Scan Begin | 30 m/z | Set End Plate Offset | -500 V | Set Dry Gas | 3.0 l/min |
| Scan End | 3000 m/z | Set Collision Cell RF | 150.0 Vpp | Set Divert Valve | Source |

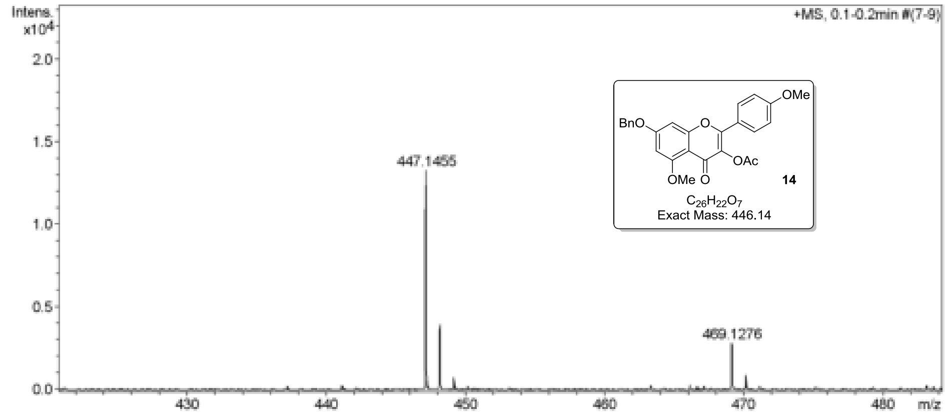


| Meas. m/z | # | Formula | Score | m/z | err [ppm] | Mean err [ppm] | mSigma | rdb | e⁻ Conf | N-Rule |
|---|---|---|---|---|---|---|---|---|---|---|
| 447.1455 | 1 | C 26 H 23 O 7 | 100.00 | 447.1438 | -3.8 | -3.5 | 9.5 | 15.5 | even | ok |
| 469.1276 | 1 | C 26 H 22 Na O 7 | 100.00 | 469.1258 | -3.8 | -2.4 | 16.0 | 15.5 | even | ok |

# Mass Spectrum SmartFormula Report

**Analysis Info**

| | | | |
|---|---|---|---|
| Analysis Name | D:\Data\USER-2014\8328.d | Acquisition Date | 9/27/2014 12:58:26 PM |
| Method | WU_tune_low_20121222.m | Operator | Ma |
| Sample Name | 8328 | Instrument / Ser# | micrOTOF-Q II 10203 |
| Comment | | | |

**Acquisition Parameter**

| | | | | | |
|---|---|---|---|---|---|
| Source Type | ESI | Ion Polarity | Positive | Set Nebulizer | 0.4 Bar |
| Focus | Not active | Set Capillary | 4500 V | Set Dry Heater | 180 °C |
| Scan Begin | 30 m/z | Set End Plate Offset | -500 V | Set Dry Gas | 3.0 l/min |
| Scan End | 3000 m/z | Set Collision Cell RF | 150.0 Vpp | Set Divert Valve | Source |

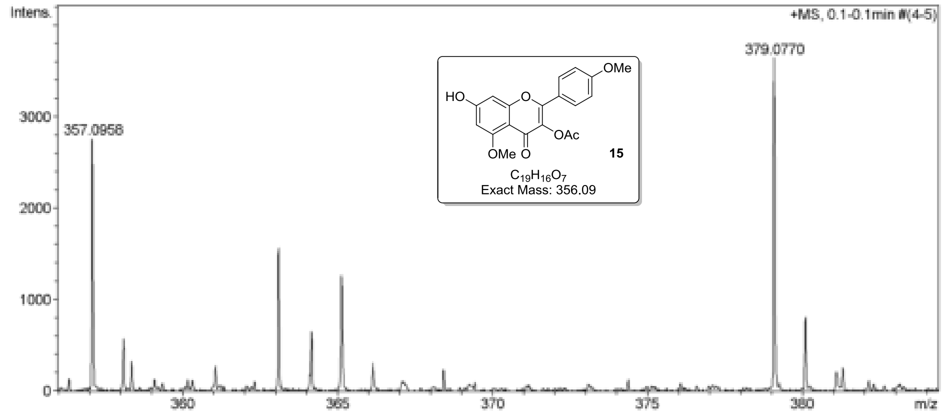


| Meas. m/z | # | Formula | Score | m/z | err [ppm] | Mean err [ppm] | mSigma | rdb | e⁻ Conf | N-Rule |
|---|---|---|---|---|---|---|---|---|---|---|
| 357.0958 | 1 | C 19 H 17 O 7 | 100.00 | 357.0969 | 3.0 | 3.4 | 20.0 | 11.5 | even | ok |
| 379.0770 | 1 | C 19 H 16 Na O 7 | 100.00 | 379.0788 | 4.9 | 3.1 | 16.9 | 11.5 | even | ok |

# Mass Spectrum SmartFormula Report

**Analysis Info**

| | | | |
|---|---|---|---|
| Analysis Name | D:\Data\USER-2014\mei0705.d | Acquisition Date | 9/18/2014 10:08:49 AM |
| Method | WU_tune_low_20121222.m | Operator | Ma |
| Sample Name | mei0705 | Instrument / Ser# | micrOTOF-Q II 10203 |
| Comment | | | |

**Acquisition Parameter**

| | | | | | |
|---|---|---|---|---|---|
| Source Type | ESI | Ion Polarity | Positive | Set Nebulizer | 0.4 Bar |
| Focus | Not active | Set Capillary | 4500 V | Set Dry Heater | 180 °C |
| Scan Begin | 30 m/z | Set End Plate Offset | -500 V | Set Dry Gas | 3.0 l/min |
| Scan End | 3000 m/z | Set Collision Cell RF | 150.0 Vpp | Set Divert Valve | Source |

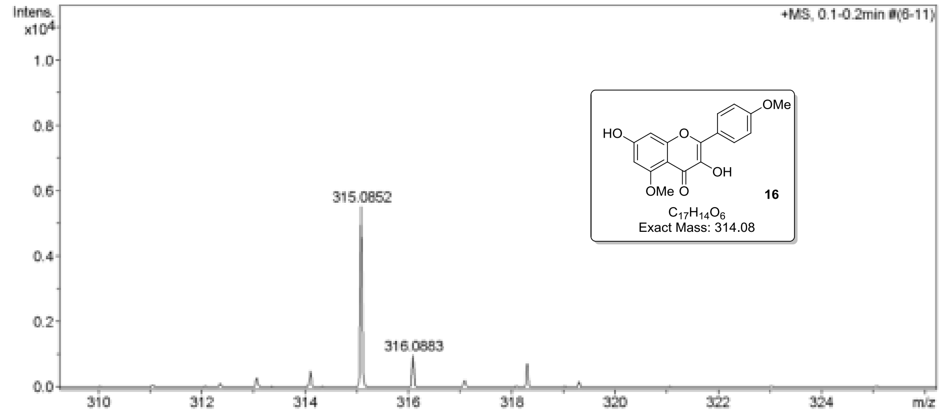


| Meas. m/z | # | Formula | Score | m/z | err [ppm] | Mean err [ppm] | mSigma | rdb | e⁻ Conf | N-Rule |
|---|---|---|---|---|---|---|---|---|---|---|
| 315.0852 | 1 | C 17 H 15 O 6 | 100.00 | 315.0863 | 3.5 | 3.6 | 16.6 | 10.5 | even | ok |

# Mass Spectrum SmartFormula Report

**Analysis Info**

| | | | |
|---|---|---|---|
| Analysis Name | D:\Data\USER-2013\MEI390-2.d | Acquisition Date | 4/16/2013 4:50:18 PM |
| Method | WU_tune_low_20121222-negative.m | Operator | Ma |
| Sample Name | MEI390-2 | Instrument / Ser# | micrOTOF-Q II 10203 |
| Comment | | | |

**Acquisition Parameter**

| | | | | | |
|---|---|---|---|---|---|
| Source Type | ESI | Ion Polarity | Negative | Set Nebulizer | 0.3 Bar |
| Focus | Not active | Set Capillary | 4000 V | Set Dry Heater | 180 °C |
| Scan Begin | 50 m/z | Set End Plate Offset | -500 V | Set Dry Gas | 4.0 l/min |
| Scan End | 3000 m/z | Set Collision Cell RF | 150.0 Vpp | Set Divert Valve | Source |

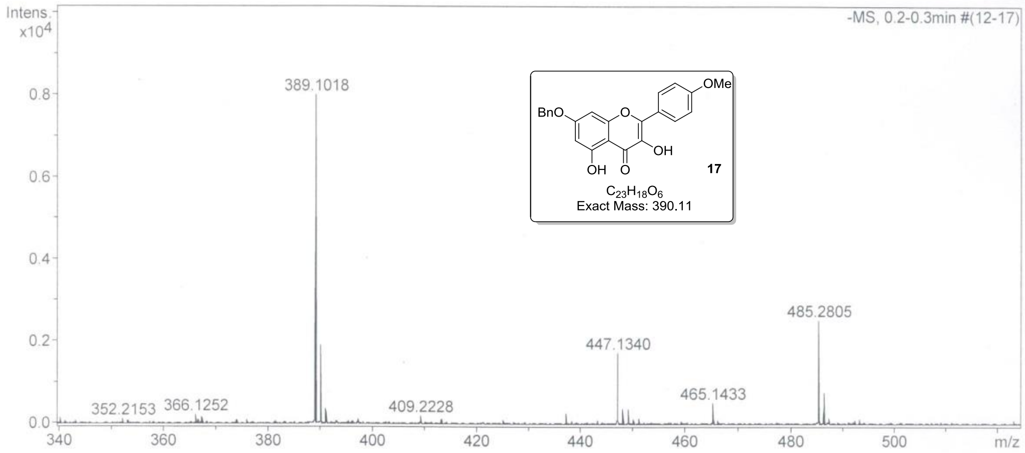


| Meas. m/z | # | Formula | Score | m/z | err [ppm] | Mean err [ppm] | mSigma | rdb | e⁻ Conf | N-Rule |
|---|---|---|---|---|---|---|---|---|---|---|
| 389.1018 | 1 | C 23 H 17 O 6 | 100.00 | 389.1031 | 3.2 | 2.6 | 5.5 | 15.5 | even | ok |

# Mass Spectrum SmartFormula Report

**Analysis Info**

| | | | |
|---|---|---|---|
| Analysis Name | D:\Data\USER-2013\mei300.d | Acquisition Date | 10/15/2013 11:45:54 AM |
| Method | WU_tune_low_20121222.m | Operator | Ma |
| Sample Name | mei300 | Instrument / Ser# | micrOTOF-Q II 10203 |
| Comment | | | |

**Acquisition Parameter**

| | | | | | | | |
|---|---|---|---|---|---|---|---|
| Source Type | ESI | Ion Polarity | Positive | Set Nebulizer | 0.4 Bar | | |
| Focus | Not active | Set Capillary | 4500 V | Set Dry Heater | 100 °C | | |
| Scan Begin | 50 m/z | Set End Plate Offset | -500 V | Set Dry Gas | 3.0 l/min | | |
| Scan End | 3000 m/z | Set Collision Cell RF | 150.0 Vpp | Set Divert Valve | Source | | |

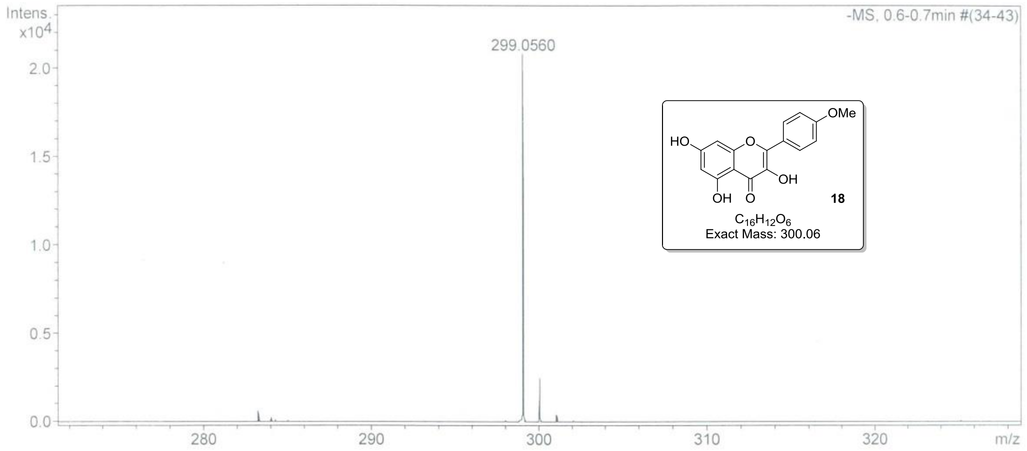


| Meas. m/z | # | Formula | Score | m/z | err [ppm] | Mean err [ppm] | mSigma | rdb | e⁻ Conf | N-Rule |
|---|---|---|---|---|---|---|---|---|---|---|
| 299.0560 | 1 | C 16 H 11 O 6 | 100.00 | 299.0561 | 0.3 | -0.2 | 32.2 | 11.5 | even | ok |

# Mass Spectrum SmartFormula Report

**Analysis Info**

| | | | |
|---|---|---|---|
| Analysis Name | D:\Data\USER-2014\mei39045.d | Acquisition Date | 4/23/2014 4:58:39 PM |
| Method | WU_tune_low_20121222.m | Operator | Ma |
| Sample Name | mei39045 | Instrument / Ser# | micrOTOF-Q II 10203 |
| Comment | | | |

**Acquisition Parameter**

| | | | | | | | |
|---|---|---|---|---|---|---|---|
| Source Type | ESI | Ion Polarity | Negative | Set Nebulizer | 0.4 Bar | | |
| Focus | Not active | Set Capillary | 4000 V | Set Dry Heater | 180 °C | | |
| Scan Begin | 50 m/z | Set End Plate Offset | -500 V | Set Dry Gas | 3.0 l/min | | |
| Scan End | 3000 m/z | Set Collision Cell RF | 150.0 Vpp | Set Divert Valve | Source | | |

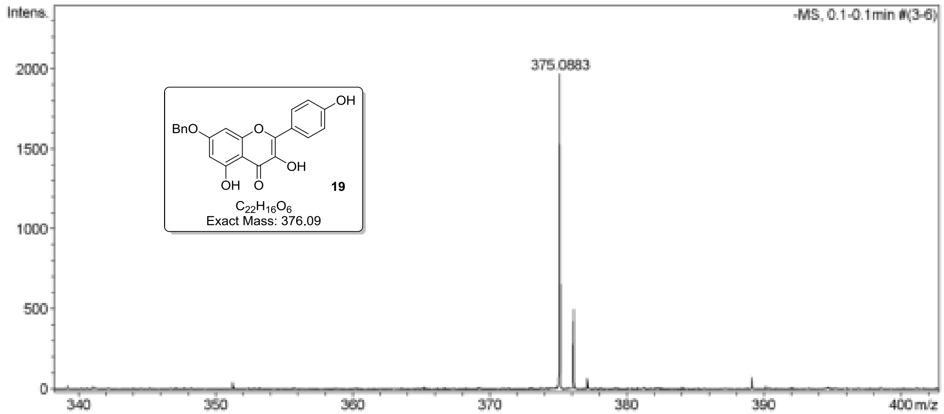


| Meas. m/z | # | Formula | Score | m/z | err [ppm] | Mean err [ppm] | mSigma | rdb | e⁻ Conf | N-Rule |
|---|---|---|---|---|---|---|---|---|---|---|
| 375.0883 | 1 | C 22 H 15 O 6 | 100.00 | 375.0874 | -2.4 | -2.4 | 24.6 | 15.5 | even | ok |

# Mass Spectrum SmartFormula Report

**Analysis Info**

| | | | |
|---|---|---|---|
| Analysis Name | D:\Data\USER-2014\mei37603+.d | Acquisition Date | 4/23/2014 5:21:10 PM |
| Method | WU_tune_low_20121222.m | Operator | Ma |
| Sample Name | mei37603+ | Instrument / Ser# | micrOTOF-Q II 10203 |
| Comment | | | |

**Acquisition Parameter**

| | | | | | |
|---|---|---|---|---|---|
| Source Type | ESI | Ion Polarity | Positive | Set Nebulizer | 0.4 Bar |
| Focus | Not active | Set Capillary | 4500 V | Set Dry Heater | 180 °C |
| Scan Begin | 50 m/z | Set End Plate Offset | -500 V | Set Dry Gas | 3.0 l/min |
| Scan End | 3000 m/z | Set Collision Cell RF | 150.0 Vpp | Set Divert Valve | Source |

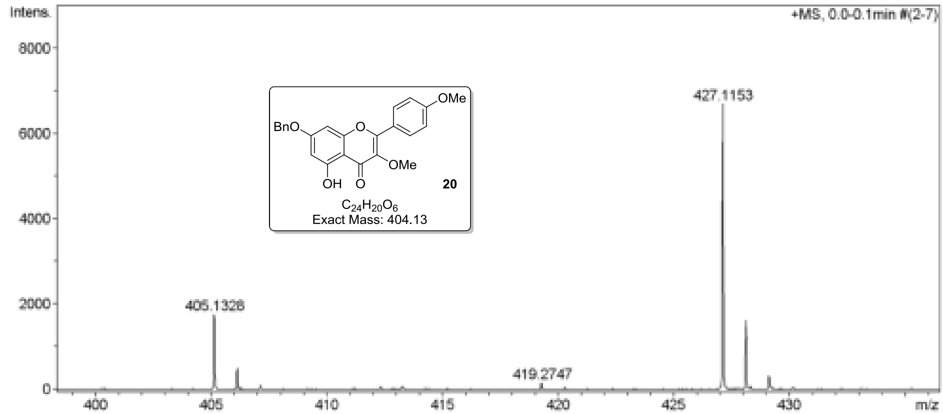


| Meas. m/z | # | Formula | Score | m/z | err [ppm] | Mean err [ppm] | mSigma | rdb | e⁻ Conf | N-Rule |
|---|---|---|---|---|---|---|---|---|---|---|
| 405.1328 | 1 | C 24 H 21 O 6 | 100.00 | 405.1333 | 1.1 | 2.2 | 30.6 | 14.5 | even | ok |
| 427.1153 | 1 | C 24 H 20 Na O 6 | 100.00 | 427.1152 | -0.2 | 0.1 | 11.5 | 14.5 | even | ok |

Bruker Compass DataAnalysis 4.0 printed: 4/23/2014 5:27:08 PM Page 1 of 1

# Mass Spectrum SmartFormula Report

**Analysis Info**

Acquisition Date 9/18/2014 10:14:43 AM

| | | | |
|---|---|---|---|
| Analysis Name | D:\Data\USER-2014\mei418sj.d | | |
| Method | WU_tune_low_20121222.m | Operator | Ma |
| Sample Name | mei418sj | Instrument / Ser# | micrOTOF-Q II 10203 |
| Comment | | | |

**Acquisition Parameter**

| | | | | | |
|---|---|---|---|---|---|
| Source Type | ESI | Ion Polarity | Positive | Set Nebulizer | 0.4 Bar |
| Focus | Not active | Set Capillary | 4500 V | Set Dry Heater | 180 °C |
| Scan Begin | 30 m/z | Set End Plate Offset | -500 V | Set Dry Gas | 3.0 l/min |
| Scan End | 3000 m/z | Set Collision Cell RF | 150.0 Vpp | Set Divert Valve | Source |

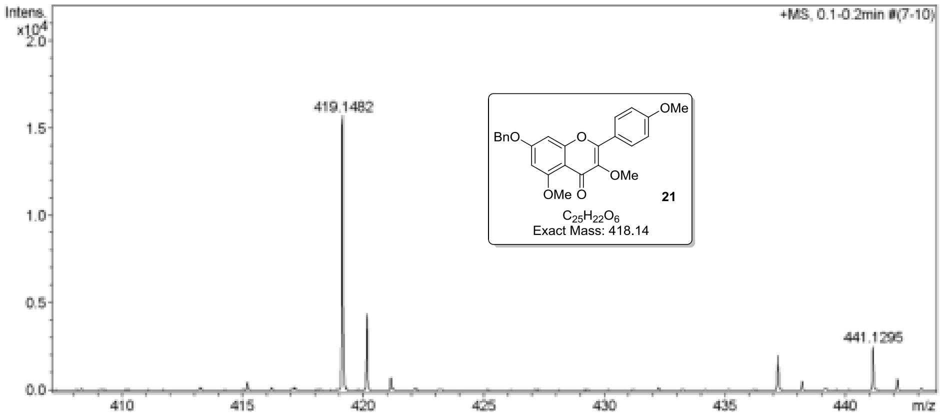


| Meas. m/z | # | Formula | Score | m/z | err [ppm] | Mean err [ppm] | mSigma | rdb | e⁻ Conf | N-Rule |
|---|---|---|---|---|---|---|---|---|---|---|
| 419.1482 | 1 | C 25 H 23 O 6 | 100.00 | 419.1489 | 1.8 | 1.7 | 3.1 | 14.5 | even | ok |
| 441.1295 | 1 | C 25 H 22 Na O 6 | 100.00 | 441.1309 | 3.1 | 2.6 | 5.5 | 14.5 | even | ok |

# Mass Spectrum SmartFormula Report

| **Analysis Info** | | Acquisition Date | 9/17/2014 2:58:17 PM |
|---|---|---|---|
| Analysis Name | D:\Data\USER-2014\mei3314.d | | |
| Method | WU_tune_low_20121222.m | Operator | Ma |
| Sample Name | mei3314 | Instrument / Ser# | micrOTOF-Q II 10203 |
| Comment | | | |

**Acquisition Parameter**

| | | | | | |
|---|---|---|---|---|---|
| Source Type | ESI | Ion Polarity | Positive | Set Nebulizer | 0.4 Bar |
| Focus | Not active | Set Capillary | 4500 V | Set Dry Heater | 180 °C |
| Scan Begin | 30 m/z | Set End Plate Offset | -500 V | Set Dry Gas | 3.0 l/min |
| Scan End | 3000 m/z | Set Collision Cell RF | 150.0 Vpp | Set Divert Valve | Source |

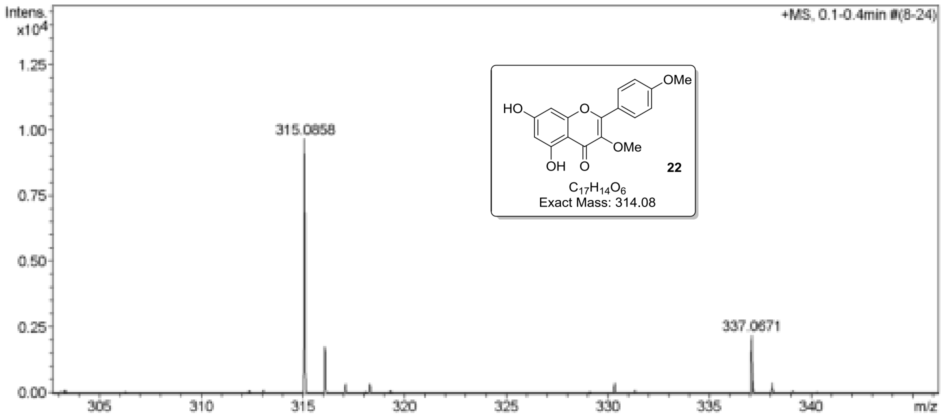


| Meas. m/z | # | Formula | Score | m/z | err [ppm] | Mean err [ppm] | mSigma | rdb | e⁻ Conf | N-Rule |
|---|---|---|---|---|---|---|---|---|---|---|
| 315.0858 | 1 | C 17 H 15 O 6 | 100.00 | 315.0863 | 1.6 | 1.6 | 107.4 | 10.5 | even | ok |
| 337.0671 | 1 | C 17 H 14 Na O 6 | 100.00 | 337.0683 | 3.5 | 3.5 | 107.4 | 10.5 | even | ok |

# Mass Spectrum SmartFormula Report

**Analysis Info**

| | | | |
|---|---|---|---|
| Analysis Name | D:\Data\USER-2014\mei9328.d | Acquisition Date | 9/17/2014 2:43:47 PM |
| Method | WU_tune_low_20121222.m | Operator | Ma |
| Sample Name | mei9328 | Instrument / Ser# | micrOTOF-Q II 10203 |
| Comment | | | |

**Acquisition Parameter**

| | | | | | | | |
|---|---|---|---|---|---|---|---|
| Source Type | ESI | Ion Polarity | Positive | | | Set Nebulizer | 0.4 Bar |
| Focus | Not active | Set Capillary | 4500 V | | | Set Dry Heater | 180 °C |
| Scan Begin | 30 m/z | Set End Plate Offset | -500 V | | | Set Dry Gas | 3.0 l/min |
| Scan End | 3000 m/z | Set Collision Cell RF | 150.0 Vpp | | | Set Divert Valve | Source |

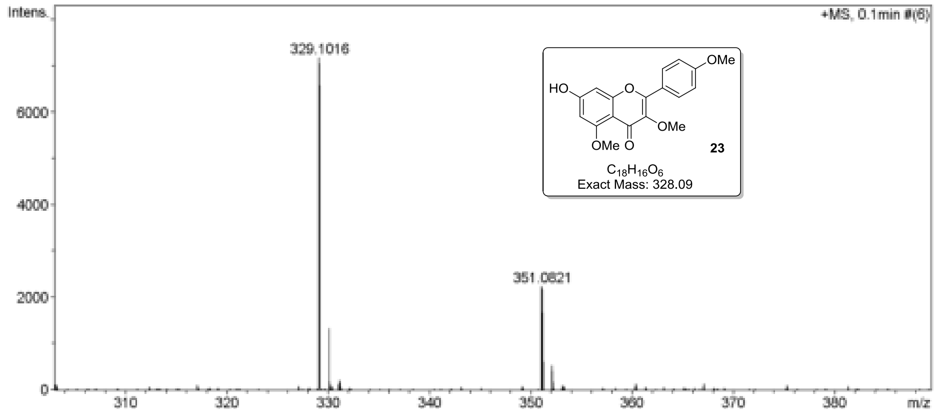


| Meas. m/z | # | Formula | Score | m/z | err [ppm] | Mean err [ppm] | mSigma | rdb | e⁻ Conf | N-Rule |
|---|---|---|---|---|---|---|---|---|---|---|
| 329.1016 | 1 | C 18 H 17 O 6 | 100.00 | 329.1020 | 1.2 | 1.8 | 18.2 | 10.5 | even | ok |
| 351.0821 | 1 | C 15 H 8 N 10 Na | 100.00 | 351.0826 | 1.2 | 1.1 | 24.6 | 16.5 | even | ok |

# NOESY Spectrum of Compound **4**

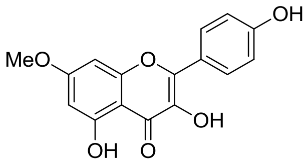


**4**

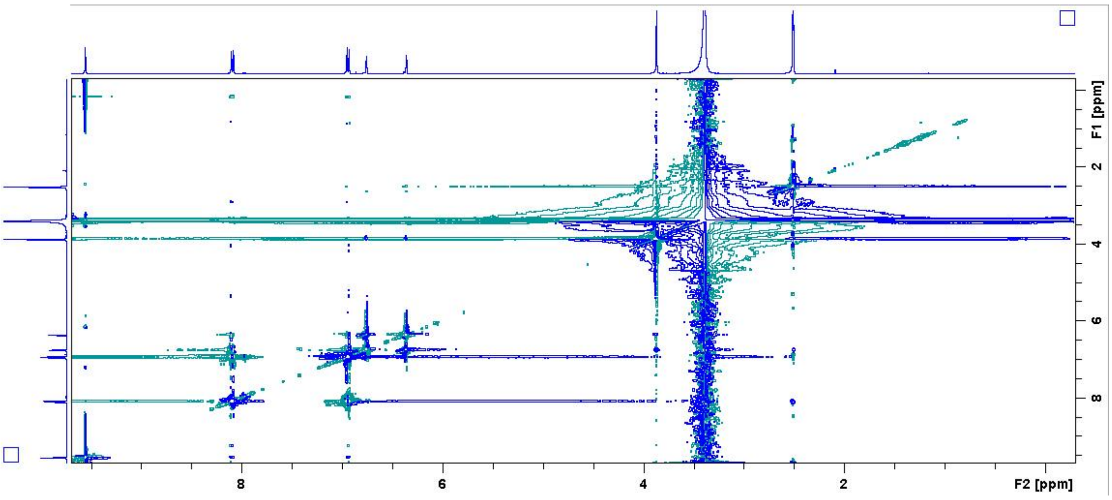


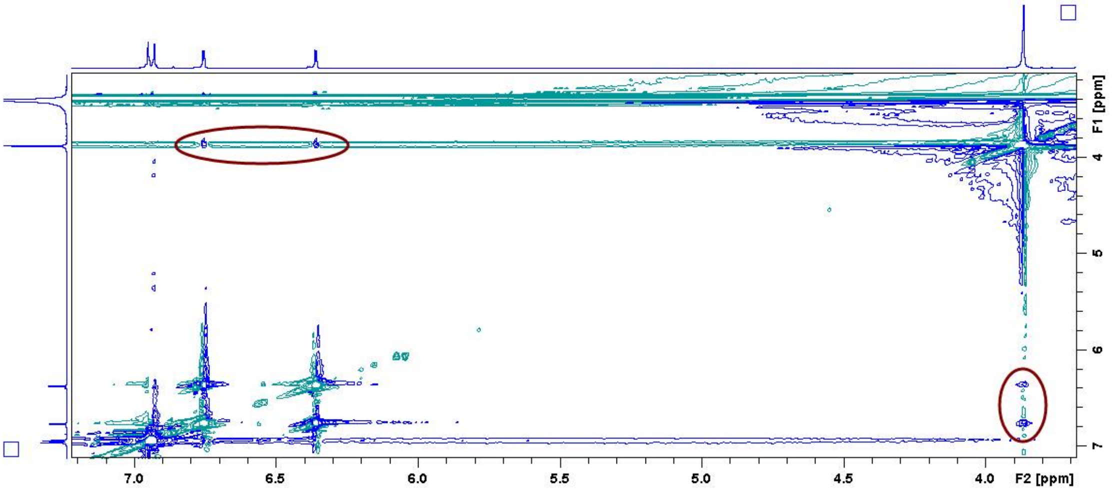

# NOESY Spectrum of Compound **13**

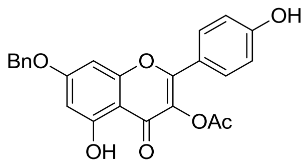


**13**

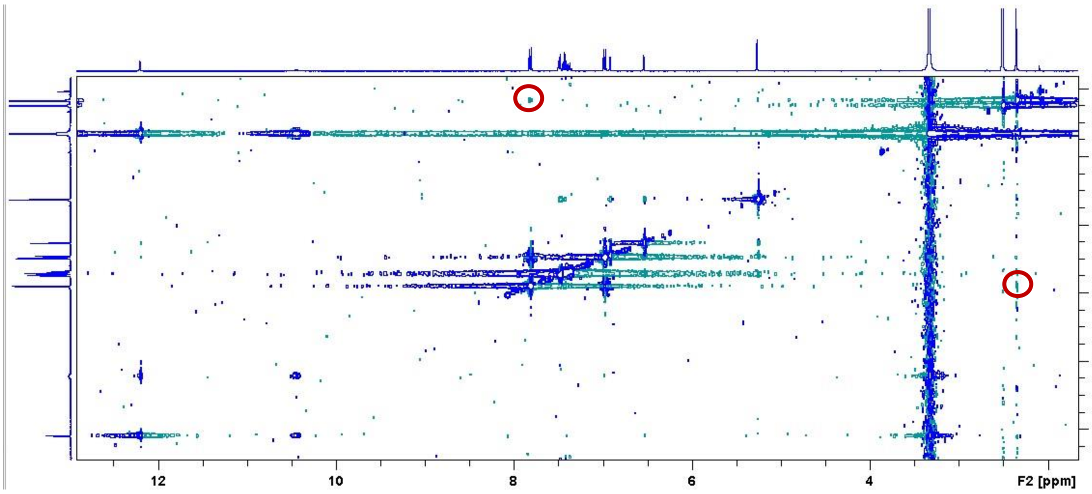


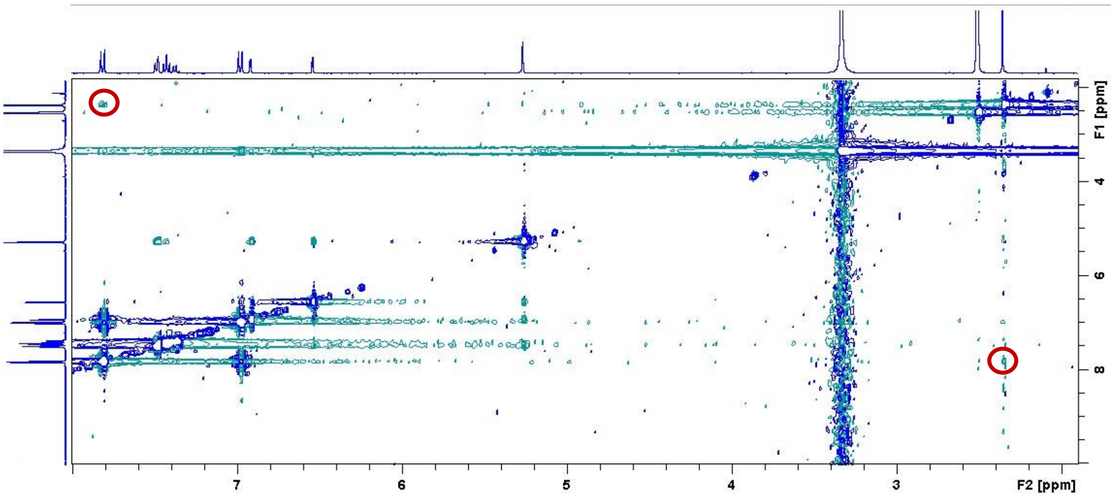

# HMBC Spectrum of Compound **4**

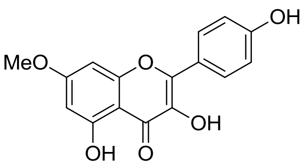


**4**

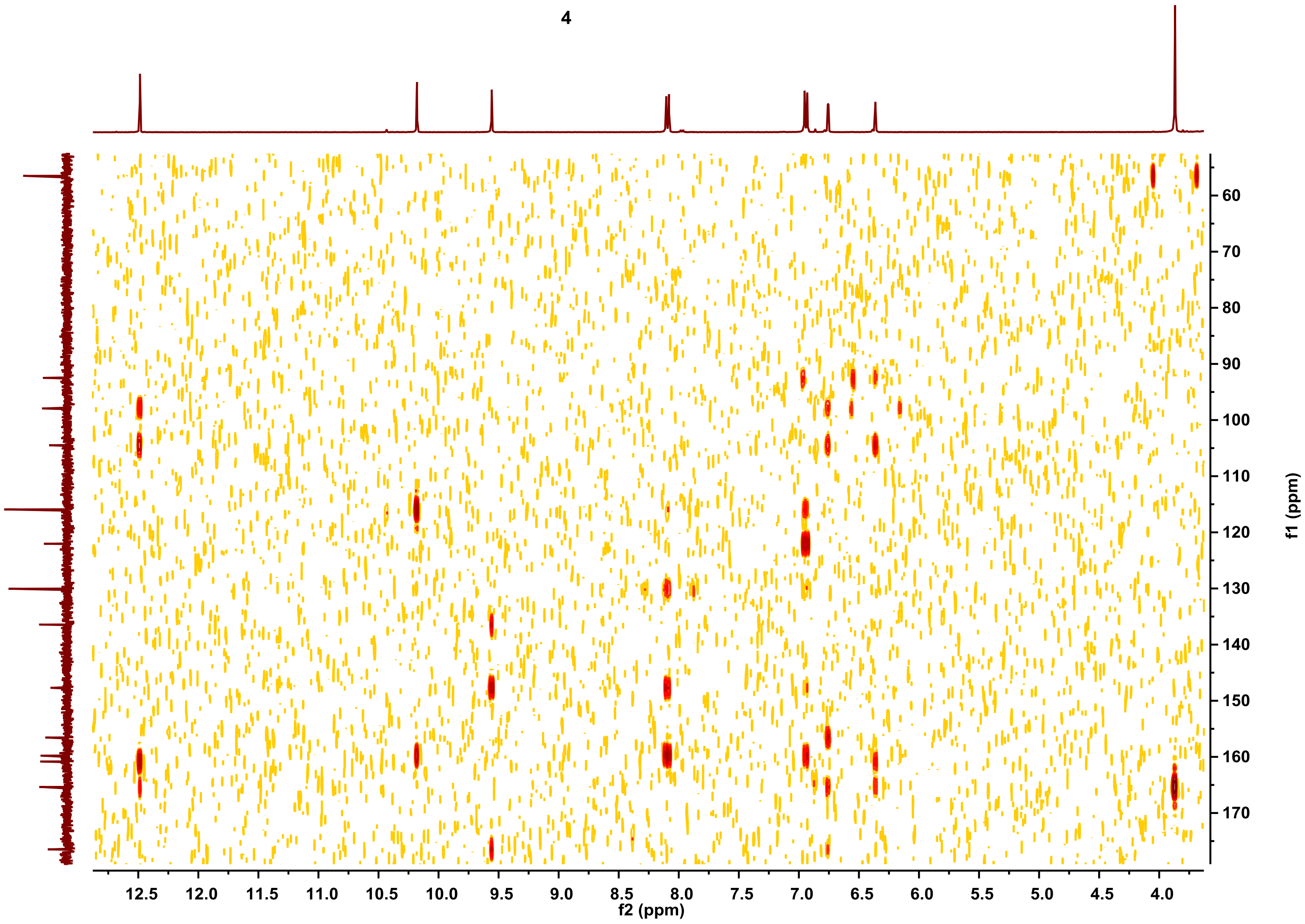

# HMBC Spectrum of Compound **5**

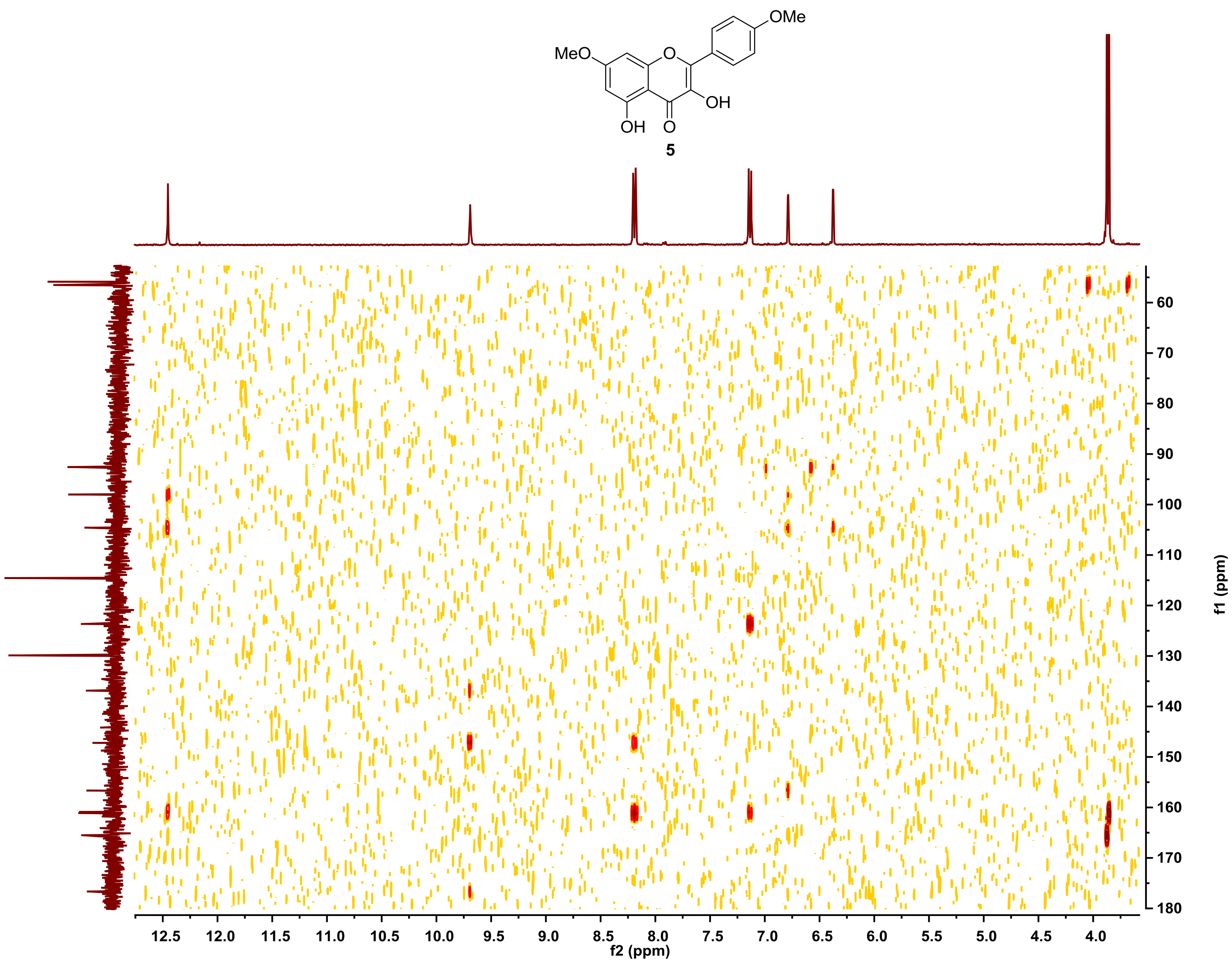

# HMBC Spectrum of Compound **7**

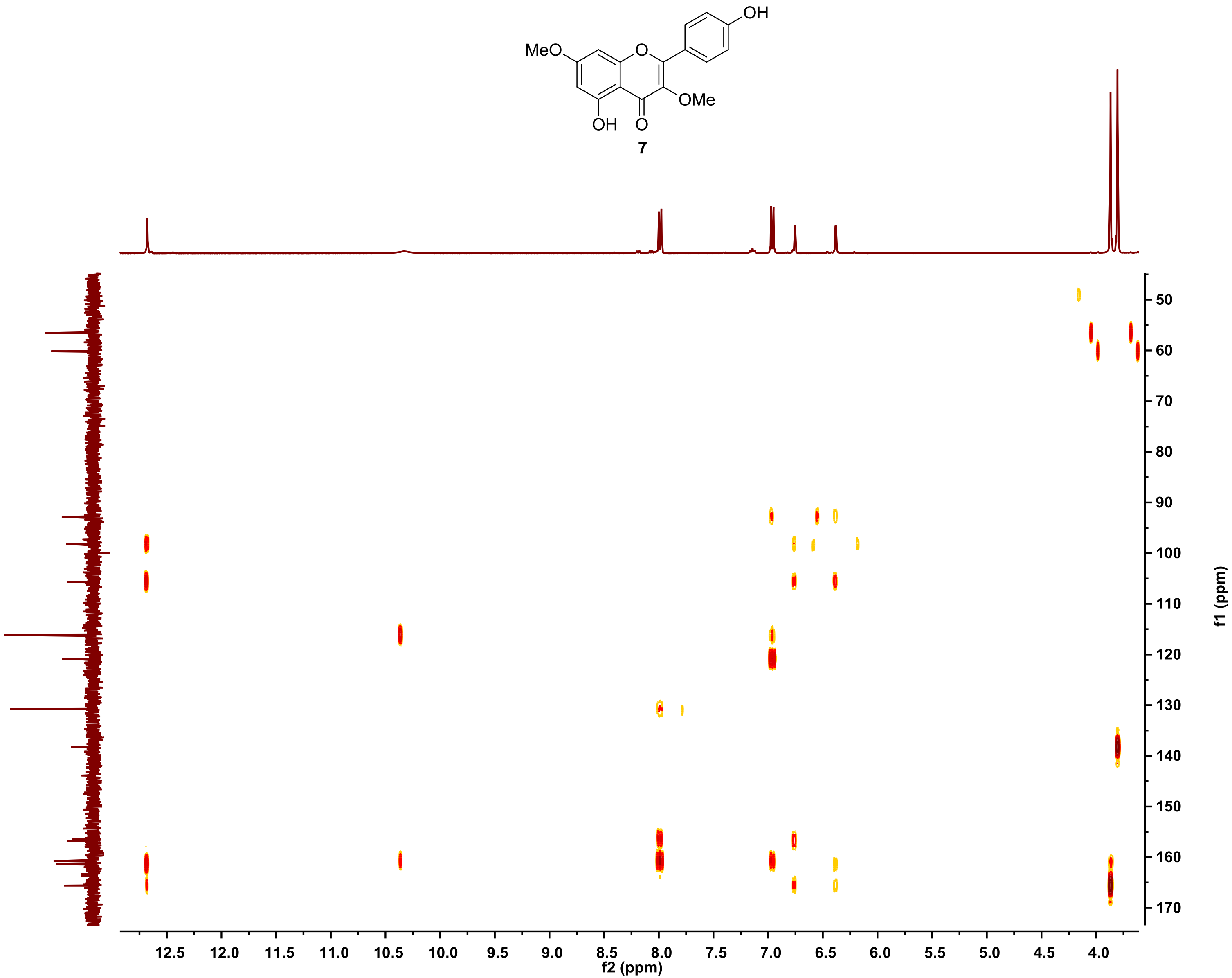

# HMBC Spectrum of Compound **9**

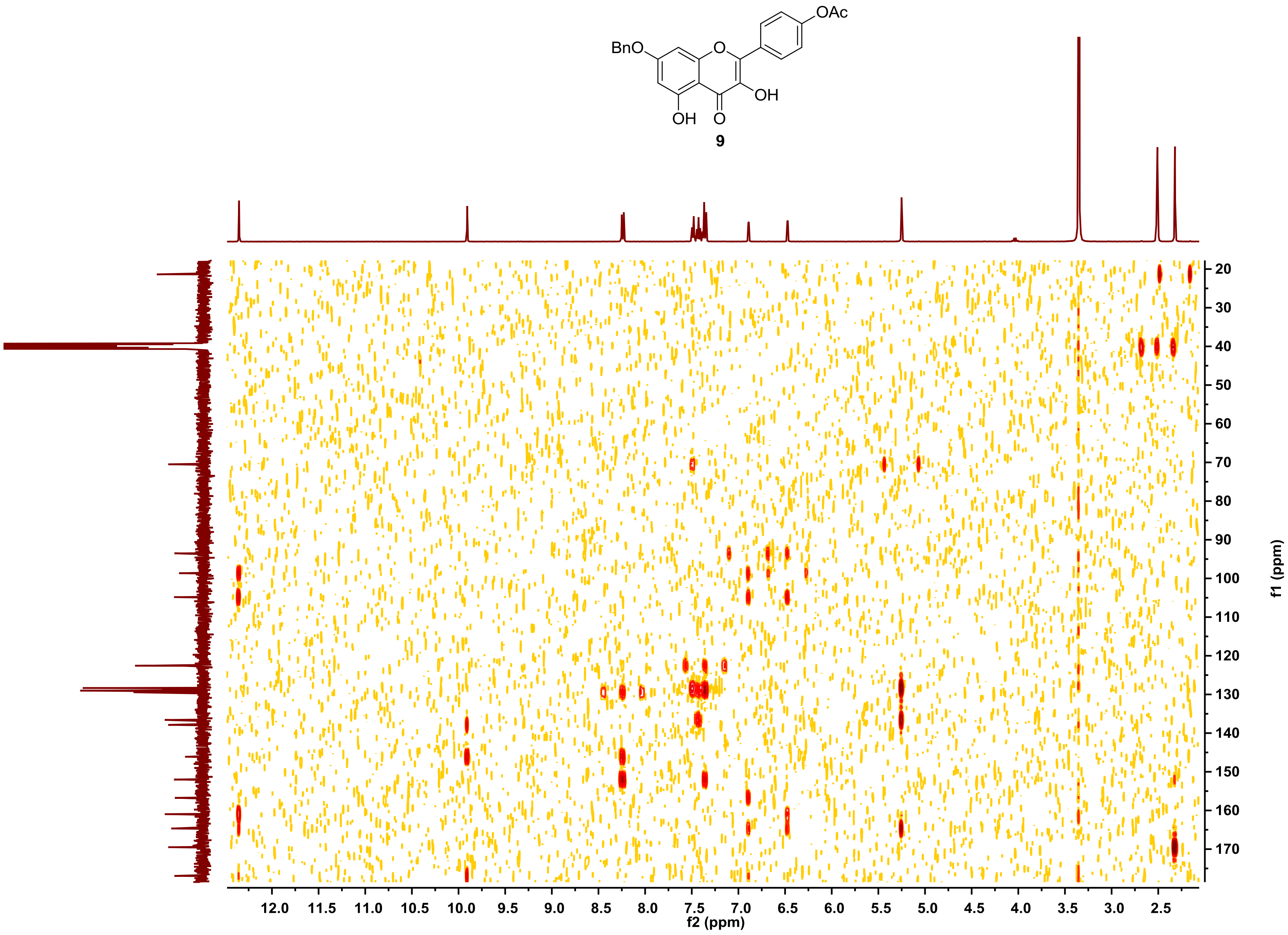

# HMBC Spectrum of Compound **12**

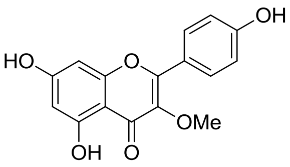


**12**

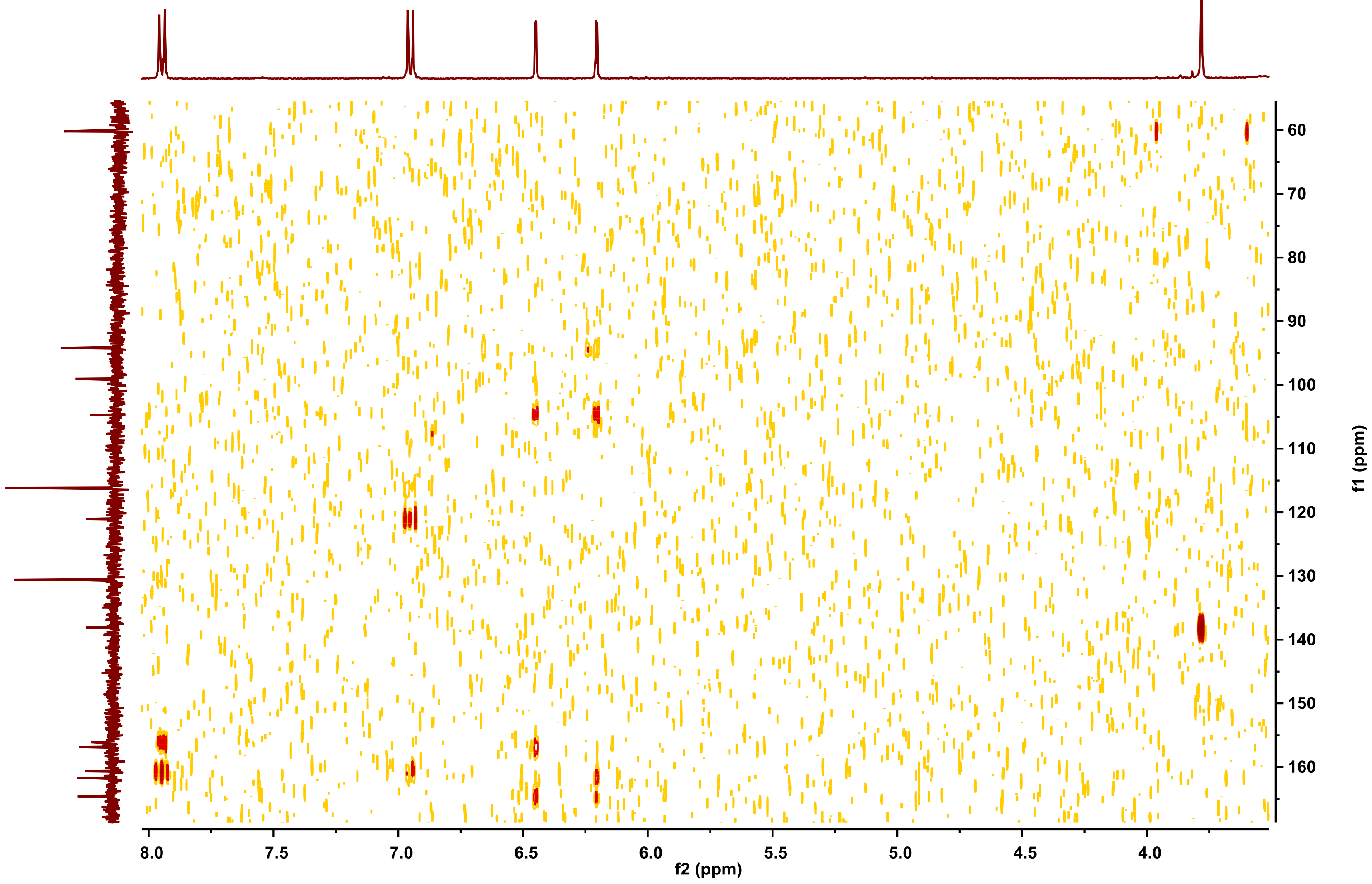

# HMBC Spectrum of Compound **13**

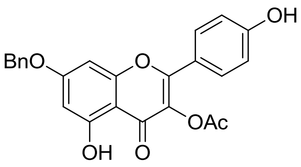


**13**

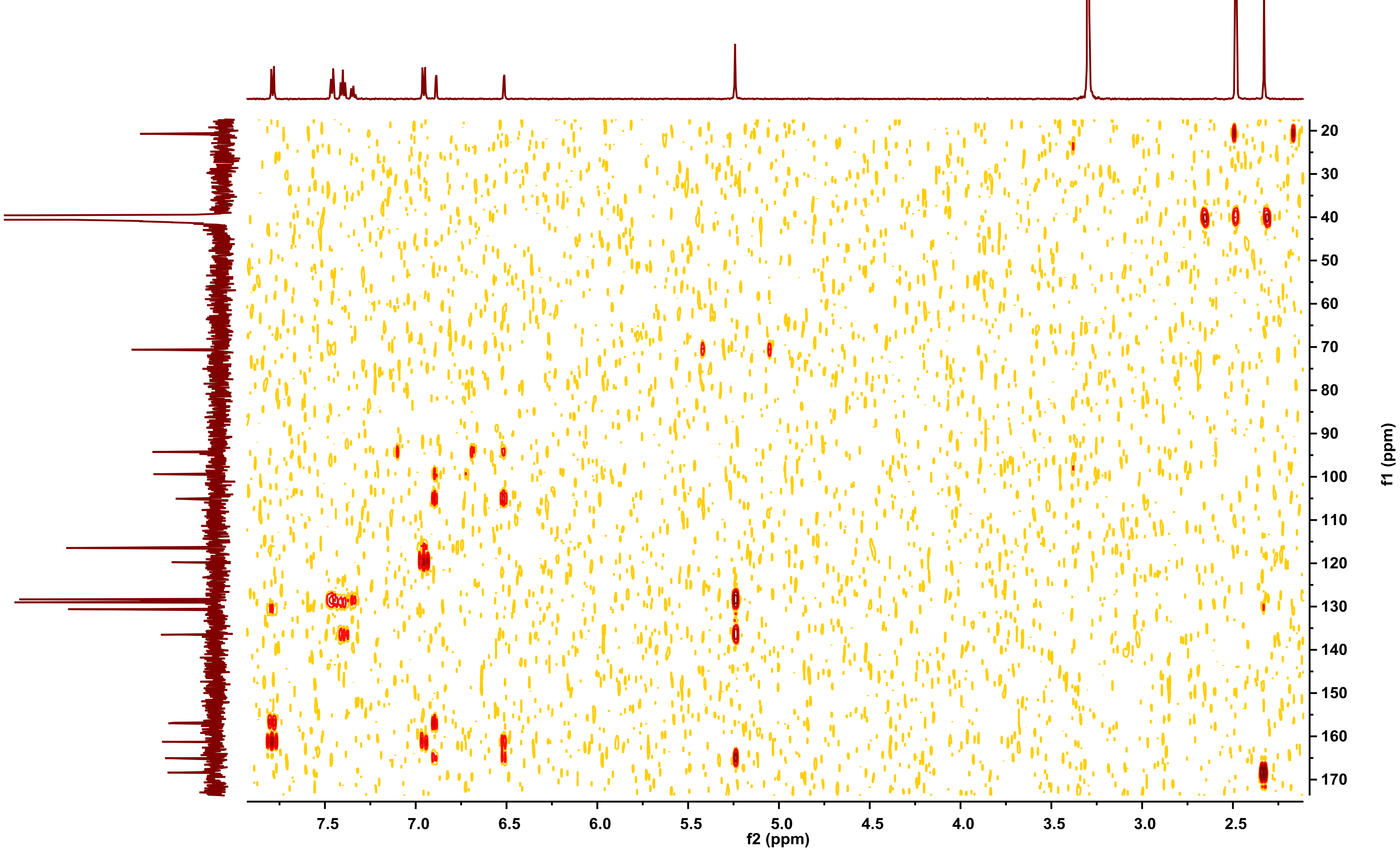

# HMBC Spectrum of Compound **16**

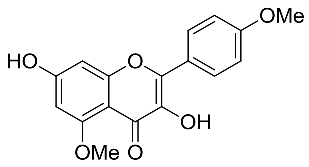


**16**

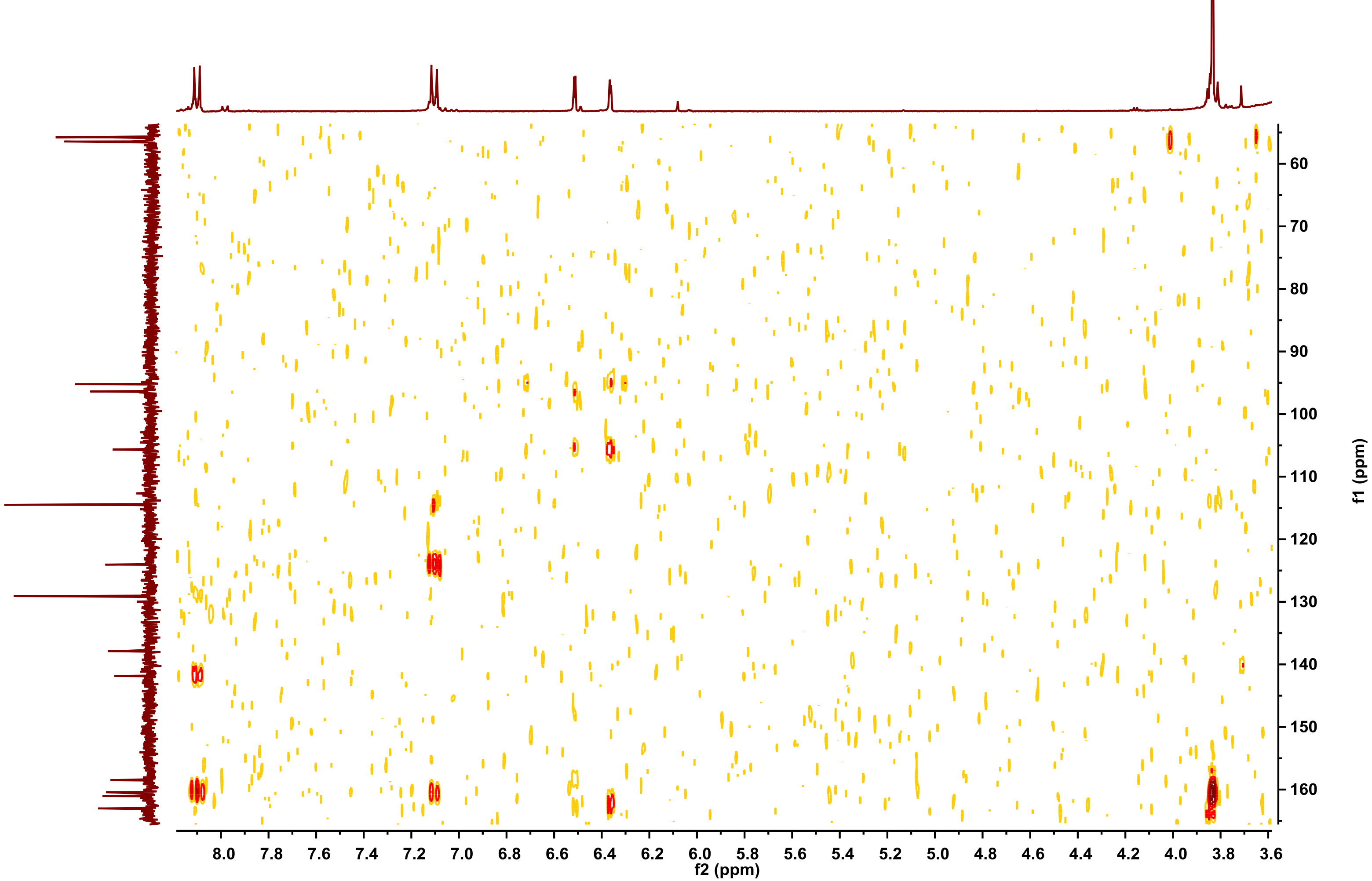

# HMBC Spectrum of Compound **18**

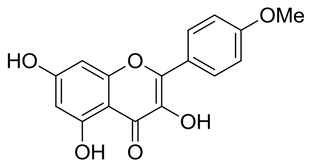


**18**

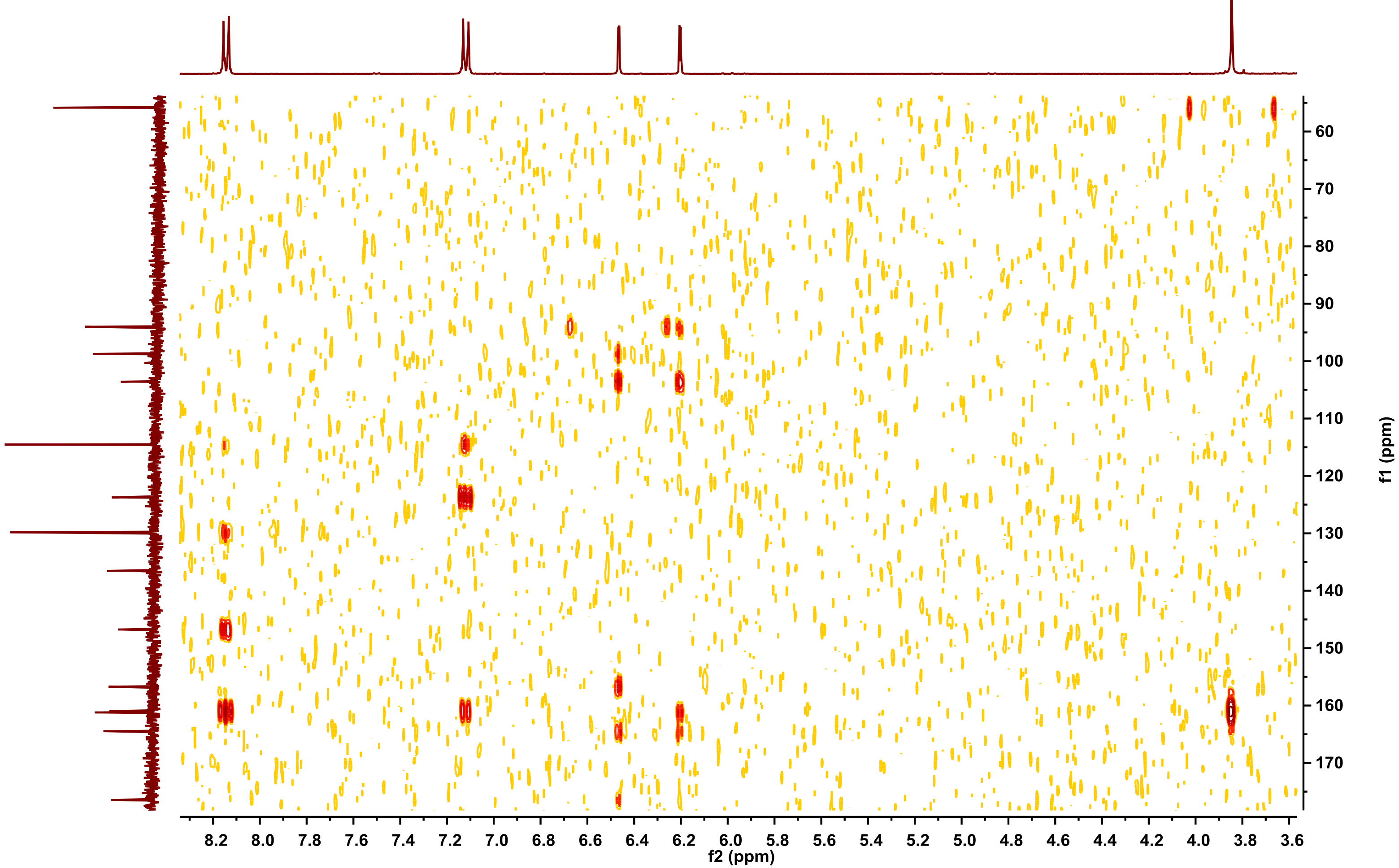

# HMBC Spectrum of Compound **22**

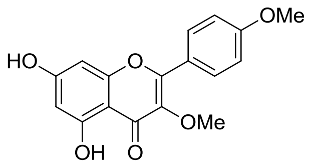


**22**

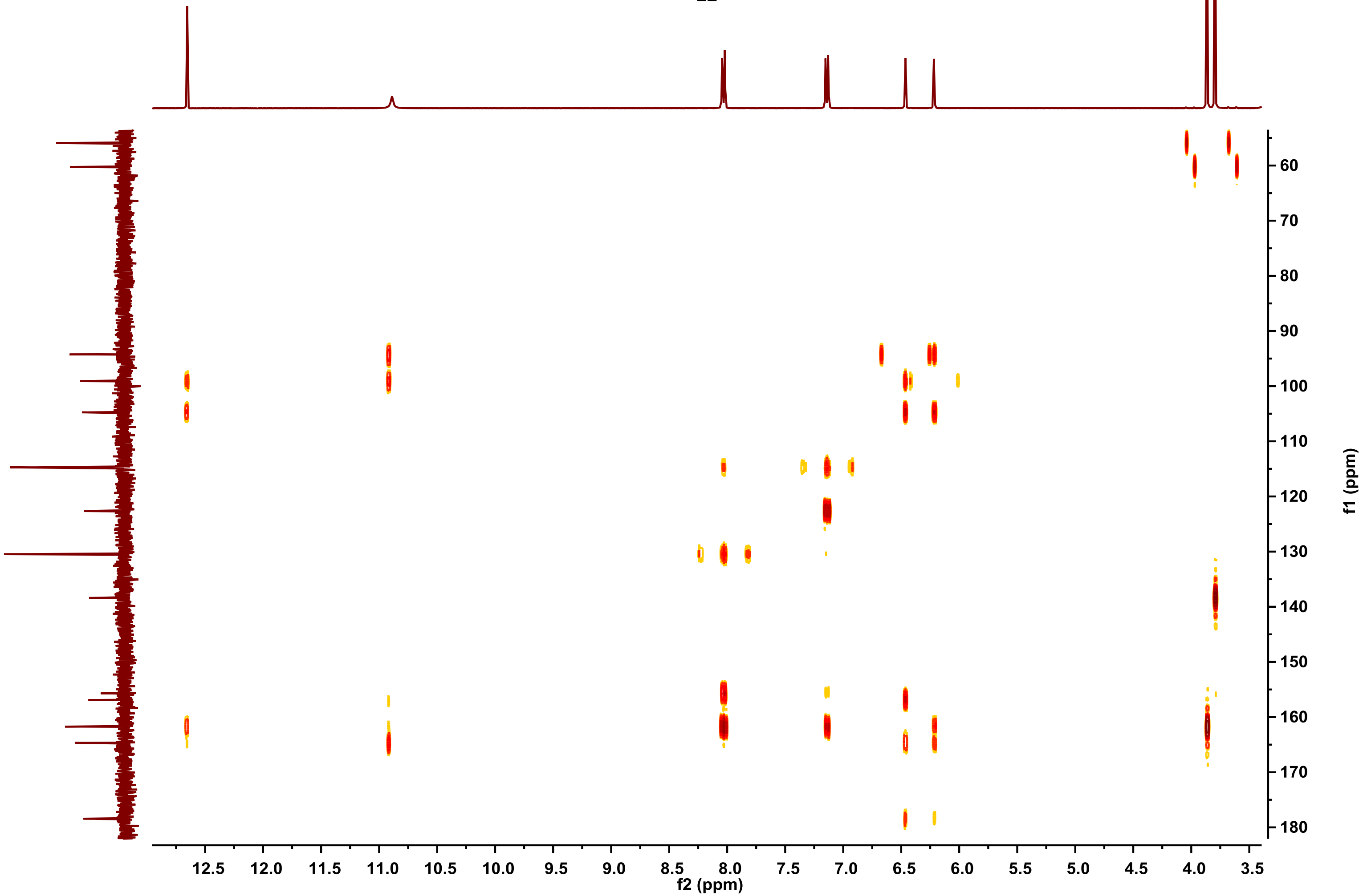

# HMBC Spectrum of Compound **23**

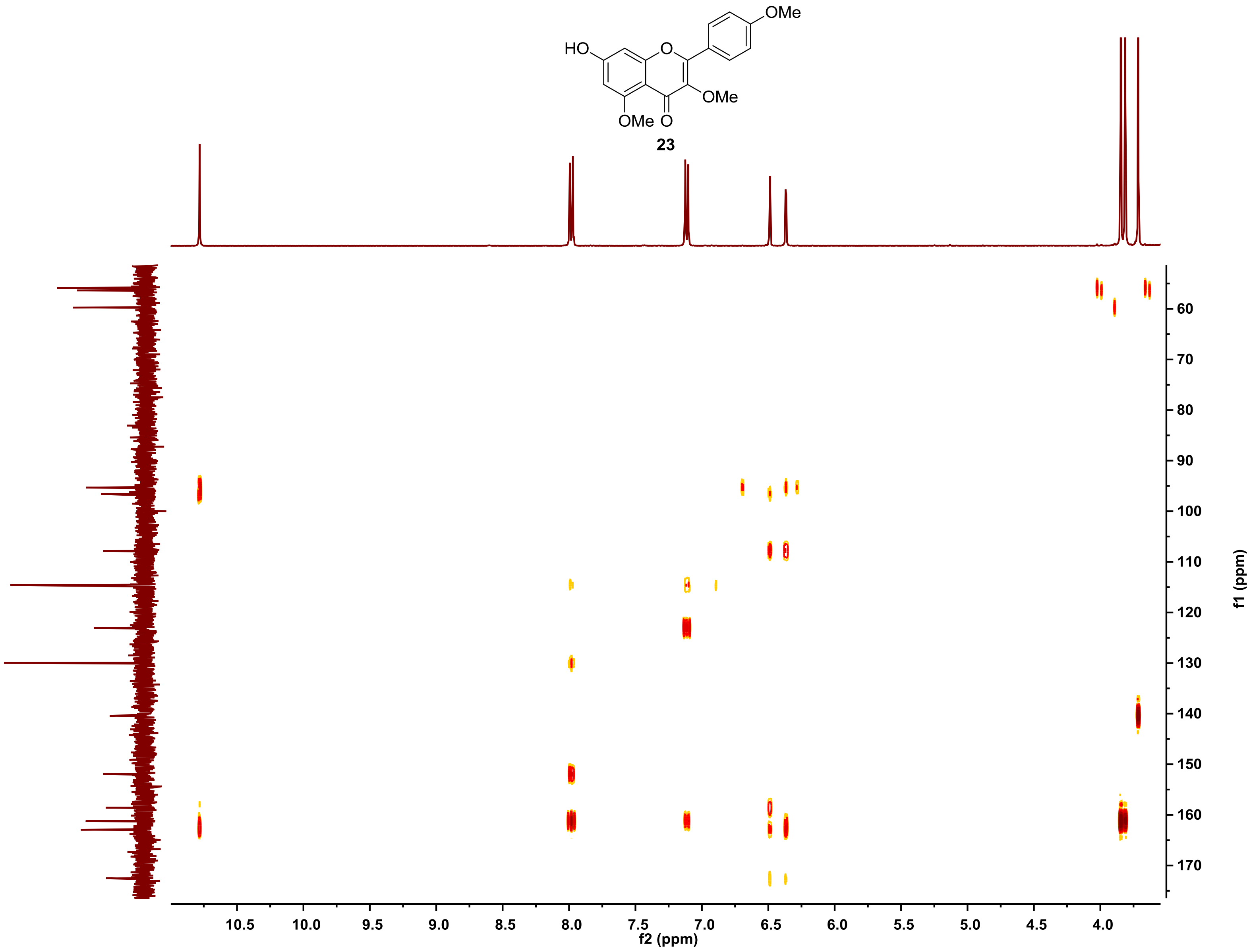

Supplement: File 1 — Experimental section; NMR and ESI-HRMS spectra of 1–23; NOESY spectra of 4 and 13; HMBC spectra of 4, 5, 7, 9, 12, 13, 16, 18, 22, 23. [file Beilstein_J_Org_Chem-11-288-s001.pdf]
